# Searches for electroweak production of charginos, neutralinos, and sleptons decaying to leptons and W, Z, and Higgs bosons in pp collisions at 8 TeV

**DOI:** 10.1140/epjc/s10052-014-3036-7

**Published:** 2014-09-26

**Authors:** The CMS Collaboration, V. Khachatryan, A. M. Sirunyan, A. Tumasyan, W. Adam, T. Bergauer, M. Dragicevic, J. Erö, C. Fabjan, M. Friedl, R. Frühwirth, V. M. Ghete, C. Hartl, N. Hörmann, J. Hrubec, M. Jeitler, W. Kiesenhofer, V. Knünz, M. Krammer, I. Krätschmer, D. Liko, I. Mikulec, D. Rabady, B. Rahbaran, H. Rohringer, R. Schöfbeck, J. Strauss, A. Taurok, W. Treberer-Treberspurg, W. Waltenberger, C.-E. Wulz, V. Mossolov, N. Shumeiko, J. Suarez Gonzalez, S. Alderweireldt, M. Bansal, S. Bansal, T. Cornelis, E. A. De Wolf, X. Janssen, A. Knutsson, S. Luyckx, S. Ochesanu, B. Roland, R. Rougny, M. Van De Klundert, H. Van Haevermaet, P. Van Mechelen, N. Van Remortel, A. Van Spilbeeck, F. Blekman, S. Blyweert, J. D’Hondt, N. Daci, N. Heracleous, A. Kalogeropoulos, J. Keaveney, T. J. Kim, S. Lowette, M. Maes, A. Olbrechts, Q. Python, D. Strom, S. Tavernier, W. Van Doninck, P. Van Mulders, G. P. Van Onsem, I. Villella, C. Caillol, B. Clerbaux, G. De Lentdecker, D. Dobur, L. Favart, A. P. R. Gay, A. Grebenyuk, A. Léonard, A. Mohammadi, L. Perniè, T. Reis, T. Seva, L. Thomas, C. Vander Velde, P. Vanlaer, J. Wang, V. Adler, K. Beernaert, L. Benucci, A. Cimmino, S. Costantini, S. Crucy, S. Dildick, A. Fagot, G. Garcia, J. Mccartin, A. A. Ocampo Rios, D. Ryckbosch, S. Salva Diblen, M. Sigamani, N. Strobbe, F. Thyssen, M. Tytgat, E. Yazgan, N. Zaganidis, S. Basegmez, C. Beluffi, G. Bruno, R. Castello, A. Caudron, L. Ceard, G. G. Da Silveira, C. Delaere, T. du Pree, D. Favart, L. Forthomme, A. Giammanco, J. Hollar, P. Jez, M. Komm, V. Lemaitre, J. Liao, C. Nuttens, D. Pagano, L. Perrini, A. Pin, K. Piotrzkowski, A. Popov, L. Quertenmont, M. Selvaggi, M. Vidal Marono, J. M. Vizan Garcia, N. Beliy, T. Caebergs, E. Daubie, G. H. Hammad, W. L. Aldá Júnior, G. A. Alves, M. Correa Martins Junior, T. Dos Reis Martins, M. E. Pol, W. Carvalho, J. Chinellato, A. Custódio, E. M. Da Costa, D. De Jesus Damiao, C. De Oliveira Martins, S. Fonseca De Souza, H. Malbouisson, M. Malek, D. Matos Figueiredo, L. Mundim, H. Nogima, W. L. Prado Da Silva, J. Santaolalla, A. Santoro, A. Sznajder, E. J. Tonelli Manganote, A. Vilela Pereira, C. A. Bernardes, F. A. Dias, T. R. Fernandez Perez Tomei, E. M. Gregores, P. G. Mercadante, S. F. Novaes, Sandra S. Padula, A. Aleksandrov, V. Genchev, P. Iaydjiev, A. Marinov, S. Piperov, M. Rodozov, G. Sultanov, M. Vutova, A. Dimitrov, I. Glushkov, R. Hadjiiska, V. Kozhuharov, L. Litov, B. Pavlov, P. Petkov, J. G. Bian, G. M. Chen, H. S. Chen, M. Chen, R. Du, C. H. Jiang, D. Liang, S. Liang, R. Plestina, J. Tao, X. Wang, Z. Wang, C. Asawatangtrakuldee, Y. Ban, Y. Guo, Q. Li, W. Li, S. Liu, Y. Mao, S. J. Qian, D. Wang, L. Zhang, W. Zou, C. Avila, L. F. Chaparro Sierra, C. Florez, J. P. Gomez, B. Gomez Moreno, J. C. Sanabria, N. Godinovic, D. Lelas, D. Polic, I. Puljak, Z. Antunovic, M. Kovac, V. Brigljevic, K. Kadija, J. Luetic, D. Mekterovic, L. Sudic, A. Attikis, G. Mavromanolakis, J. Mousa, C. Nicolaou, F. Ptochos, P. A. Razis, M. Bodlak, M. Finger, M. Finger, Y. Assran, A. Ellithi Kamel, M. A. Mahmoud, A. Radi, M. Kadastik, M. Murumaa, M. Raidal, A. Tiko, P. Eerola, G. Fedi, M. Voutilainen, J. Härkönen, V. Karimäki, R. Kinnunen, M. J. Kortelainen, T. Lampén, K. Lassila-Perini, S. Lehti, T. Lindén, P. Luukka, T. Mäenpää, T. Peltola, E. Tuominen, J. Tuominiemi, E. Tuovinen, L. Wendland, T. Tuuva, M. Besancon, F. Couderc, M. Dejardin, D. Denegri, B. Fabbro, J. L. Faure, C. Favaro, F. Ferri, S. Ganjour, A. Givernaud, P. Gras, G. Hamel de Monchenault, P. Jarry, E. Locci, J. Malcles, J. Rander, A. Rosowsky, M. Titov, S. Baffioni, F. Beaudette, P. Busson, C. Charlot, T. Dahms, M. Dalchenko, L. Dobrzynski, N. Filipovic, A. Florent, R. Granier de Cassagnac, L. Mastrolorenzo, P. Miné, C. Mironov, I. N. Naranjo, M. Nguyen, C. Ochando, P. Paganini, R. Salerno, J. B. Sauvan, Y. Sirois, C. Veelken, Y. Yilmaz, A. Zabi, J.-L. Agram, J. Andrea, A. Aubin, D. Bloch, J.-M. Brom, E. C. Chabert, C. Collard, E. Conte, J.-C. Fontaine, D. Gelé, U. Goerlach, C. Goetzmann, A.-C. Le Bihan, P. Van Hove, S. Gadrat, S. Beauceron, N. Beaupere, G. Boudoul, S. Brochet, C. A. Carrillo Montoya, J. Chasserat, R. Chierici, D. Contardo, P. Depasse, H. El Mamouni, J. Fan, J. Fay, S. Gascon, M. Gouzevitch, B. Ille, T. Kurca, M. Lethuillier, L. Mirabito, S. Perries, J. D. Ruiz Alvarez, D. Sabes, L. Sgandurra, V. Sordini, M. Vander Donckt, P. Verdier, S. Viret, H. Xiao, Z. Tsamalaidze, C. Autermann, S. Beranek, M. Bontenackels, M. Edelhoff, L. Feld, O. Hindrichs, K. Klein, A. Ostapchuk, A. Perieanu, F. Raupach, J. Sammet, S. Schael, H. Weber, B. Wittmer, V. Zhukov, M. Ata, E. Dietz-Laursonn, D. Duchardt, M. Erdmann, R. Fischer, A. Güth, T. Hebbeker, C. Heidemann, K. Hoepfner, D. Klingebiel, S. Knutzen, P. Kreuzer, M. Merschmeyer, A. Meyer, M. Olschewski, K. Padeken, P. Papacz, H. Reithler, S. A. Schmitz, L. Sonnenschein, D. Teyssier, S. Thüer, M. Weber, V. Cherepanov, Y. Erdogan, G. Flügge, H. Geenen, M. Geisler, W. Haj Ahmad, F. Hoehle, B. Kargoll, T. Kress, Y. Kuessel, J. Lingemann, A. Nowack, I. M. Nugent, L. Perchalla, O. Pooth, A. Stahl, I. Asin, N. Bartosik, J. Behr, W. Behrenhoff, U. Behrens, A. J. Bell, M. Bergholz, A. Bethani, K. Borras, A. Burgmeier, A. Cakir, L. Calligaris, A. Campbell, S. Choudhury, F. Costanza, C. Diez Pardos, S. Dooling, T. Dorland, G. Eckerlin, D. Eckstein, T. Eichhorn, G. Flucke, J. Garay Garcia, A. Geiser, P. Gunnellini, J. Hauk, G. Hellwig, M. Hempel, D. Horton, H. Jung, M. Kasemann, P. Katsas, J. Kieseler, C. Kleinwort, D. Krücker, W. Lange, J. Leonard, K. Lipka, A. Lobanov, W. Lohmann, B. Lutz, R. Mankel, I. Marfin, I.-A. Melzer-Pellmann, A. B. Meyer, J. Mnich, A. Mussgiller, S. Naumann-Emme, A. Nayak, O. Novgorodova, F. Nowak, E. Ntomari, H. Perrey, D. Pitzl, R. Placakyte, A. Raspereza, P. M. Ribeiro Cipriano, E. Ron, M. Ö. Sahin, J. Salfeld-Nebgen, P. Saxena, R. Schmidt, T. Schoerner-Sadenius, M. Schröder, S. Spannagel, A. D. R. Vargas Trevino, R. Walsh, C. Wissing, M. Aldaya Martin, V. Blobel, M. Centis Vignali, J. Erfle, E. Garutti, K. Goebel, M. Görner, J. Haller, M. Hoffmann, R. S. Höing, H. Kirschenmann, R. Klanner, R. Kogler, J. Lange, T. Lapsien, T. Lenz, I. Marchesini, J. Ott, T. Peiffer, N. Pietsch, D. Rathjens, C. Sander, H. Schettler, P. Schleper, E. Schlieckau, A. Schmidt, M. Seidel, J. Sibille, V. Sola, H. Stadie, G. Steinbrück, D. Troendle, E. Usai, L. Vanelderen, C. Barth, C. Baus, J. Berger, C. Böser, E. Butz, T. Chwalek, W. De Boer, A. Descroix, A. Dierlamm, M. Feindt, F. Frensch, M. Giffels, F. Hartmann, T. Hauth, U. Husemann, I. Katkov, A. Kornmayer, E. Kuznetsova, P. Lobelle Pardo, M. U. Mozer, Th. Müller, A. Nürnberg, G. Quast, K. Rabbertz, F. Ratnikov, S. Röcker, H. J. Simonis, F. M. Stober, R. Ulrich, J. Wagner-Kuhr, S. Wayand, T. Weiler, R. Wolf, G. Anagnostou, G. Daskalakis, T. Geralis, V. A. Giakoumopoulou, A. Kyriakis, D. Loukas, A. Markou, C. Markou, A. Psallidas, I. Topsis-Giotis, A. Panagiotou, N. Saoulidou, E. Stiliaris, X. Aslanoglou, I. Evangelou, G. Flouris, C. Foudas, P. Kokkas, N. Manthos, I. Papadopoulos, E. Paradas, G. Bencze, C. Hajdu, P. Hidas, D. Horvath, F. Sikler, V. Veszpremi, G. Vesztergombi, A. J. Zsigmond, N. Beni, S. Czellar, J. Karancsi, J. Molnar, J. Palinkas, Z. Szillasi, P. Raics, Z. L. Trocsanyi, B. Ujvari, S. K. Swain, S. B. Beri, V. Bhatnagar, N. Dhingra, R. Gupta, U. Bhawandeep, A. K. Kalsi, M. Kaur, M. Mittal, N. Nishu, J. B. Singh, Ashok Kumar, Arun Kumar, S. Ahuja, A. Bhardwaj, B. C. Choudhary, A. Kumar, S. Malhotra, M. Naimuddin, K. Ranjan, V. Sharma, S. Banerjee, S. Bhattacharya, K. Chatterjee, S. Dutta, B. Gomber, Sa. Jain, Sh. Jain, R. Khurana, A. Modak, S. Mukherjee, D. Roy, S. Sarkar, M. Sharan, A. Abdulsalam, D. Dutta, S. Kailas, V. Kumar, A. K. Mohanty, L. M. Pant, P. Shukla, A. Topkar, T. Aziz, S. Banerjee, R. M. Chatterjee, R. K. Dewanjee, S. Dugad, S. Ganguly, S. Ghosh, M. Guchait, A. Gurtu, G. Kole, S. Kumar, M. Maity, G. Majumder, K. Mazumdar, G. B. Mohanty, B. Parida, K. Sudhakar, N. Wickramage, H. Bakhshiansohi, H. Behnamian, S. M. Etesami, A. Fahim, R. Goldouzian, A. Jafari, M. Khakzad, M. Mohammadi Najafabadi, M. Naseri, S. Paktinat Mehdiabadi, B. Safarzadeh, M. Zeinali, M. Felcini, M. Grunewald, M. Abbrescia, L. Barbone, C. Calabria, S. S. Chhibra, A. Colaleo, D. Creanza, N. De Filippis, M. De Palma, L. Fiore, G. Iaselli, G. Maggi, M. Maggi, S. My, S. Nuzzo, A. Pompili, G. Pugliese, R. Radogna, G. Selvaggi, L. Silvestris, G. Singh, R. Venditti, P. Verwilligen, G. Zito, G. Abbiendi, A. C. Benvenuti, D. Bonacorsi, S. Braibant-Giacomelli, L. Brigliadori, R. Campanini, P. Capiluppi, A. Castro, F. R. Cavallo, G. Codispoti, M. Cuffiani, G. M. Dallavalle, F. Fabbri, A. Fanfani, D. Fasanella, P. Giacomelli, C. Grandi, L. Guiducci, S. Marcellini, G. Masetti, A. Montanari, F. L. Navarria, A. Perrotta, F. Primavera, A. M. Rossi, T. Rovelli, G. P. Siroli, N. Tosi, R. Travaglini, S. Albergo, G. Cappello, M. Chiorboli, S. Costa, F. Giordano, R. Potenza, A. Tricomi, C. Tuve, G. Barbagli, V. Ciulli, C. Civinini, R. D’Alessandro, E. Focardi, E. Gallo, S. Gonzi, V. Gori, P. Lenzi, M. Meschini, S. Paoletti, G. Sguazzoni, A. Tropiano, L. Benussi, S. Bianco, F. Fabbri, D. Piccolo, F. Ferro, M. Lo Vetere, E. Robutti, S. Tosi, M. E. Dinardo, S. Fiorendi, S. Gennai, R. Gerosa, A. Ghezzi, P. Govoni, M. T. Lucchini, S. Malvezzi, R. A. Manzoni, A. Martelli, B. Marzocchi, D. Menasce, L. Moroni, M. Paganoni, D. Pedrini, S. Ragazzi, N. Redaelli, T. Tabarelli de Fatis, S. Buontempo, N. Cavallo, S. Di Guida, F. Fabozzi, A. O. M. Iorio, L. Lista, S. Meola, M. Merola, P. Paolucci, P. Azzi, N. Bacchetta, M. Biasotto, D. Bisello, A. Branca, R. Carlin, P. Checchia, M. Dall’Osso, T. Dorigo, U. Dosselli, F. Fanzago, M. Galanti, F. Gasparini, U. Giubilato, A. Gozzelino, K. Kanishchev, S. Lacaprara, M. Margoni, A. T. Meneguzzo, J. Pazzini, N. Pozzobon, P. Ronchese, F. Simonetto, E. Torassa, M. Tosi, P. Zotto, A. Zucchetta, M. Gabusi, S. P. Ratti, C. Riccardi, P. Salvini, P. Vitulo, M. Biasini, G. M. Bilei, D. Ciangottini, L. Fanò, P. Lariccia, G. Mantovani, M. Menichelli, F. Romeo, A. Saha, A. Santocchia, A. Spiezia, K. Androsov, P. Azzurri, G. Bagliesi, J. Bernardini, T. Boccali, G. Broccolo, R. Castaldi, M. A. Ciocci, R. Dell’Orso, S. Donato, F. Fiori, L. Foà, A. Giassi, M. T. Grippo, F. Ligabue, T. Lomtadze, L. Martini, A. Messineo, C. S. Moon, F. Palla, A. Rizzi, A. Savoy-Navarro, A. T. Serban, P. Spagnolo, P. Squillacioti, R. Tenchini, G. Tonelli, A. Venturi, P. G. Verdini, C. Vernieri, L. Barone, F. Cavallari, D. Del Re, M. Diemoz, M. Grassi, C. Jorda, E. Longo, F. Margaroli, P. Meridiani, F. Micheli, S. Nourbakhsh, G. Organtini, R. Paramatti, S. Rahatlou, C. Rovelli, F. Santanastasio, L. Soffi, P. Traczyk, N. Amapane, R. Arcidiacono, S. Argiro, M. Arneodo, R. Bellan, C. Biino, N. Cartiglia, S. Casasso, M. Costa, A. Degano, N. Demaria, L. Finco, C. Mariotti, S. Maselli, E. Migliore, V. Monaco, M. Musich, M. M. Obertino, G. Ortona, L. Pacher, N. Pastrone, M. Pelliccioni, G. L. Pinna Angioni, A. Potenza, A. Romero, M. Ruspa, R. Sacchi, A. Solano, A. Staiano, U. Tamponi, S. Belforte, V. Candelise, M. Casarsa, F. Cossutti, G. Della Ricca, B. Gobbo, C. La Licata, M. Marone, D. Montanino, A. Schizzi, T. Umer, A. Zanetti, S. Chang, A. Kropivnitskaya, S. K. Nam, D. H. Kim, G. N. Kim, M. S. Kim, M. S. Kim, D. J. Kong, S. Lee, Y. D. Oh, H. Park, A. Sakharov, D. C. Son, J. Y. Kim, S. Song, S. Choi, D. Gyun, B. Hong, M. Jo, H. Kim, Y. Kim, B. Lee, K. S. Lee, S. K. Park, Y. Roh, M. Choi, J. H. Kim, I. C. Park, S. Park, G. Ryu, M. S. Ryu, Y. Choi, Y. K. Choi, J. Goh, E. Kwon, J. Lee, H. Seo, I. Yu, A. Juodagalvis, J. R. Komaragiri, H. Castilla-Valdez, E. De La Cruz-Burelo, I. Heredia-de La Cruz, R. Lopez-Fernandez, A. Sanchez-Hernandez, S. Carrillo Moreno, F. Vazquez Valencia, I. Pedraza, H. A. Salazar Ibarguen, E. Casimiro Linares, A. Morelos Pineda, D. Krofcheck, P. H. Butler, S. Reucroft, A. Ahmad, M. Ahmad, Q. Hassan, H. R. Hoorani, S. Khalid, W. A. Khan, T. Khurshid, M. A. Shah, M. Shoaib, H. Bialkowska, M. Bluj, B. Boimska, T. Frueboes, M. Górski, M. Kazana, K. Nawrocki, K. Romanowska-Rybinska, M. Szleper, P. Zalewski, G. Brona, K. Bunkowski, M. Cwiok, W. Dominik, K. Doroba, A. Kalinowski, M. Konecki, J. Krolikowski, M. Misiura, M. Olszewski, W. Wolszczak, P. Bargassa, C. Beirão Da Cruz E Silva, P. Faccioli, P. G. Ferreira Parracho, M. Gallinaro, F. Nguyen, J. Rodrigues Antunes, J. Seixas, J. Varela, P. Vischia, I. Golutvin, I. Gorbunov, V. Karjavin, V. Konoplyanikov, G. Kozlov, A. Lanev, A. Malakhov, V. Matveev, P. Moisenz, V. Palichik, V. Perelygin, M. Savina, S. Shmatov, S. Shulha, N. Skatchkov, V. Smirnov, B. S. Yuldashev, A. Zarubin, V. Golovtsov, Y. Ivanov, V. Kim, P. Levchenko, V. Murzin, V. Oreshkin, I. Smirnov, V. Sulimov, L. Uvarov, S. Vavilov, A. Vorobyev, An. Vorobyev, Yu. Andreev, A. Dermenev, S. Gninenko, N. Golubev, M. Kirsanov, N. Krasnikov, A. Pashenkov, D. Tlisov, A. Toropin, V. Epshteyn, V. Gavrilov, N. Lychkovskaya, V. Popov, G. Safronov, S. Semenov, A. Spiridonov, V. Stolin, E. Vlasov, A. Zhokin, V. Andreev, M. Azarkin, I. Dremin, M. Kirakosyan, A. Leonidov, G. Mesyats, S. V. Rusakov, A. Vinogradov, A. Belyaev, E. Boos, V. Bunichev, M. Dubinin, L. Dudko, A. Gribushin, V. Klyukhin, O. Kodolova, I. Lokhtin, S. Obraztsov, S. Petrushanko, V. Savrin, A. Snigirev, I. Azhgirey, I. Bayshev, S. Bitioukov, V. Kachanov, A. Kalinin, D. Konstantinov, V. Krychkine, V. Petrov, R. Ryutin, A. Sobol, L. Tourtchanovitch, S. Troshin, N. Tyurin, A. Uzunian, A. Volkov, P. Adzic, M. Dordevic, M. Ekmedzic, J. Milosevic, J. Alcaraz Maestre, C. Battilana, E. Calvo, M. Cerrada, M. Chamizo Llatas, N. Colino, B. De La Cruz, A. Delgado Peris, D. Domínguez Vázquez, A. Escalante Del Valle, C. Fernandez Bedoya, J. P. Fernández Ramos, J. Flix, M. C. Fouz, P. Garcia-Abia, O. Gonzalez Lopez, S. Goy Lopez, J. M. Hernandez, M. I. Josa, G. Merino, E. Navarro De Martino, A. Pérez-Calero Yzquierdo, J. Puerta Pelayo, A. Quintario Olmeda, I. Redondo, L. Romero, M. S. Soares, C. Albajar, J. F. de Trocóniz, M. Missiroli, H. Brun, J. Cuevas, J. Fernandez Menendez, S. Folgueras, I. Gonzalez Caballero, L. Lloret Iglesias, J. A. Brochero Cifuentes, I. J. Cabrillo, A. Calderon, J. Duarte Campderros, M. Fernandez, G. Gomez, A. Graziano, A. Lopez Virto, J. Marco, R. Marco, C. Martinez Rivero, F. Matorras, F. J. Munoz Sanchez, J. Piedra Gomez, T. Rodrigo, A. Y. Rodríguez-Marrero, A. Ruiz-Jimeno, L. Scodellaro, I. Vila, R. Vilar Cortabitarte, D. Abbaneo, E. Auffray, G. Auzinger, M. Bachtis, P. Baillon, A. H. Ball, D. Barney, A. Benaglia, J. Bendavid, L. Benhabib, J. F. Benitez, C. Bernet, G. Bianchi, P. Bloch, A. Bocci, A. Bonato, O. Bondu, C. Botta, H. Breuker, T. Camporesi, G. Cerminara, S. Colafranceschi, M. D’Alfonso, D. d’Enterria, A. Dabrowski, A. David, F. De Guio, A. De Roeck, S. De Visscher, M. Dobson, N. Dupont-Sagorin, A. Elliott-Peisert, J. Eugster, G. Franzoni, W. Funk, D. Gigi, K. Gill, D. Giordano, M. Girone, F. Glege, R. Guida, S. Gundacker, M. Guthoff, R. Guida, J. Hammer, M. Hansen, P. Harris, J. Hegeman, V. Innocente, P. Janot, K. Kousouris, K. Krajczar, P. Lecoq, C. Lourenço, N. Magini, L. Malgeri, M. Mannelli, J. Marrouche, L. Masetti, F. Meijers, S. Mersi, E. Meschi, F. Moortgat, S. Morovic, M. Mulders, P. Musella, L. Orsini, L. Pape, E. Perez, L. Perrozzi, A. Petrilli, G. Petrucciani, A. Pfeiffer, M. Pierini, M. Pimiä, D. Piparo, M. Plagge, A. Racz, G. Rolandi, M. Rovere, H. Sakulin, C. Schäfer, C. Schwick, S. Sekmen, A. Sharma, P. Siegrist, P. Silva, M. Simon, P. Sphicas, D. Spiga, J. Steggemann, B. Stieger, M. Stoye, D. Treille, A. Tsirou, G. I. Veres, J. R. Vlimant, N. Wardle, H. K. Wöhri, W. D. Zeuner, W. Bertl, K. Deiters, W. Erdmann, R. Horisberger, Q. Ingram, H. C. Kaestli, S. König, D. Kotlinski, U. Langenegger, D. Renker, T. Rohe, F. Bachmair, L. Bäni, L. Bianchini, P. Bortignon, M. A. Buchmann, B. Casal, N. Chanon, A. Deisher, G. Dissertori, M. Dittmar, M. Donegà, M. Dünser, P. Eller, C. Grab, D. Hits, W. Lustermann, B. Mangano, A. C. Marini, P. Martinez Ruiz del Arbol, D. Meister, N. Mohr, C. Nägeli, P. Nef, F. Nessi-Tedaldi, F. Pandolfi, F. Pauss, M. Peruzzi, M. Quittnat, L. Rebane, M. Rossini, A. Starodumov, M. Takahashi, K. Theofilatos, R. Wallny, H. A. Weber, C. Amsler, M. F. Canelli, V. Chiochia, A. De Cosa, A. Hinzmann, T. Hreus, B. Kilminster, B. Millan Mejias, J. Ngadiuba, P. Robmann, F. J. Ronga, H. Snoek, S. Taroni, M. Verzetti, Y. Yang, M. Cardaci, K. H. Chen, C. Ferro, C. M. Kuo, W. Lin, Y. J. Lu, R. Volpe, S. S. Yu, P. Chang, Y. H. Chang, Y. W. Chang, Y. Chao, K. F. Chen, P. H. Chen, C. Dietz, U. Grundler, W.-S. Hou, K. Y. Kao, Y. J. Lei, Y. F. Liu, R.-S. Lu, D. Majumder, E. Petrakou, Y. M. Tzeng, R. Wilken, B. Asavapibhop, N. Srimanobhas, N. Suwonjandee, A. Adiguzel, M. N. Bakirci, S. Cerci, C. Dozen, I. Dumanoglu, E. Eskut, S. Girgis, G. Gokbulut, E. Gurpinar, I. Hos, E. E. Kangal, A. Kayis Topaksu, G. Onengut, K. Ozdemir, S. Ozturk, A. Polatoz, K. Sogut, D. Sunar Cerci, B. Tali, H. Topakli, M. Vergili, I. V. Akin, B. Bilin, S. Bilmis, H. Gamsizkan, G. Karapinar, K. Ocalan, U. E. Surat, M. Yalvac, M. Zeyrek, E. Gülmez, B. Isildak, M. Kaya, O. Kaya, H. Bahtiyar, E. Barlas, K. Cankocak, F. I. Vardarlı, M. Yücel, L. Levchuk, P. Sorokin, J. J. Brooke, E. Clement, D. Cussans, H. Flacher, R. Frazier, J. Goldstein, M. Grimes, G. P. Heath, H. F. Heath, J. Jacob, L. Kreczko, C. Lucas, Z. Meng, D. M. Newbold, S. Paramesvaran, A. Poll, S. Senkin, V. J. Smith, T. Williams, K. W. Bell, A. Belyaev, C. Brew, R. M. Brown, D. J. A. Cockerill, J. A. Coughlan, K. Harder, S. Harper, E. Olaiya, D. Petyt, C. H. Shepherd-Themistocleous, A. Thea, I. R. Tomalin, W. J. Womersley, S. D. Worm, M. Baber, R. Bainbridge, O. Buchmuller, D. Burton, D. Colling, N. Cripps, M. Cutajar, P. Dauncey, G. Davies, M. Della Negra, P. Dunne, W. Ferguson, J. Fulcher, D. Futyan, A. Gilbert, G. Hall, G. Iles, M. Jarvis, G. Karapostoli, M. Kenzie, R. Lane, R. Lucas, L. Lyons, A.-M. Magnan, S. Malik, B. Mathias, J. Nash, A. Nikitenko, J. Pela, M. Pesaresi, K. Petridis, D. M. Raymond, S. Rogerson, A. Rose, C. Seez, P. Sharp, A. Tapper, M. Vazquez Acosta, T. Virdee, J. E. Cole, P. R. Hobson, A. Khan, P. Kyberd, D. Leggat, D. Leslie, W. Martin, I. D. Reid, P. Symonds, L. Teodorescu, M. Turner, J. Dittmann, K. Hatakeyama, A. Kasmi, H. Liu, T. Scarborough, O. Charaf, S. I. Cooper, C. Henderson, P. Rumerio, A. Avetisyan, T. Bose, C. Fantasia, A. Heister, P. Lawson, C. Richardson, J. Rohlf, D. Sperka, J. St. John, L. Sulak, J. Alimena, S. Bhattacharya, G. Christopher, D. Cutts, Z. Demiragli, A. Ferapontov, A. Garabedian, U. Heintz, S. Jabeen, G. Kukartsev, E. Laird, G. Landsberg, M. Luk, M. Narain, M. Segala, T. Sinthuprasith, T. Speer, J. Swanson, R. Breedon, G. Breto, M. Calderon De La Barca Sanchez, S. Chauhan, M. Chertok, J. Conway, R. Conway, P. T. Cox, R. Erbacher, M. Gardner, W. Ko, R. Lander, T. Miceli, M. Mulhearn, D. Pellett, J. Pilot, F. Ricci-Tam, M. Searle, S. Shalhout, J. Smith, M. Squires, D. Stolp, M. Tripathi, S. Wilbur, R. Yohay, R. Cousins, P. Everaerts, C. Farrell, J. Hauser, M. Ignatenko, G. Rakness, E. Takasugi, V. Valuev, M. Weber, J. Babb, R. Clare, J. Ellison, J. W. Gary, G. Hanson, J. Heilman, M. Ivova Rikova, P. Jandir, E. Kennedy, F. Lacroix, H. Liu, O. R. Long, A. Luthra, M. Malberti, H. Nguyen, A. Shrinivas, S. Sumowidagdo, S. Wimpenny, W. Andrews, J. G. Branson, G. B. Cerati, S. Cittolin, R. T. D’Agnolo, D. Evans, A. Holzner, R. Kelley, D. Klein, M. Lebourgeois, J. Letts, I. Macneill, D. Olivito, S. Padhi, C. Palmer, M. Pieri, M. Sani, V. Sharma, S. Simon, E. Sudano, M. Tadel, Y. Tu, A. Vartak, C. Welke, F. Würthwein, A. Yagil, J. Yoo, D. Barge, J. Bradmiller-Feld, C. Campagnari, T. Danielson, A. Dishaw, K. Flowers, M. Franco Sevilla, P. Geffert, C. George, F. Golf, L. Gouskos, J. Gran, J. Incandela, C. Justus, N. Mccoll, J. Richman, D. Stuart, W. To, C. West, A. Apresyan, A. Bornheim, J. Bunn, Y. Chen, E. Di Marco, J. Duarte, A. Mott, H. B. Newman, C. Pena, C. Rogan, M. Spiropulu, V. Timciuc, R. Wilkinson, S. Xie, R. Y. Zhu, V. Azzolini, A. Calamba, T. Ferguson, Y. Iiyama, M. Paulini, J. Russ, H. Vogel, I. Vorobiev, J. P. Cumalat, B. R. Drell, W. T. Ford, A. Gaz, E. Luiggi Lopez, U. Nauenberg, J. G. Smith, K. Stenson, K. A. Ulmer, S. R. Wagner, J. Alexander, A. Chatterjee, J. Chu, S. Dittmer, N. Eggert, W. Hopkins, N. Mirman, G. Nicolas Kaufman, J. R. Patterson, A. Ryd, E. Salvati, L. Skinnari, W. Sun, W. D. Teo, J. Thom, J. Thompson, J. Tucker, Y. Weng, L. Winstrom, P. Wittich, D. Winn, S. Abdullin, M. Albrow, J. Anderson, G. Apollinari, L. A. T. Bauerdick, A. Beretvas, J. Berryhill, P. C. Bhat, K. Burkett, J. N. Butler, H. W. K. Cheung, F. Chlebana, S. Cihangir, V. D. Elvira, I. Fisk, J. Freeman, E. Gottschalk, L. Gray, D. Green, S. Grünendahl, O. Gutsche, J. Hanlon, D. Hare, R. M. Harris, J. Hirschauer, B. Hooberman, S. Jindariani, M. Johnson, U. Joshi, K. Kaadze, B. Klima, B. Kreis, S. Kwan, J. Linacre, D. Lincoln, R. Lipton, T. Liu, J. Lykken, K. Maeshima, J. M. Marraffino, V. I. Martinez Outschoorn, S. Maruyama, D. Mason, P. McBride, K. Mishra, S. Mrenna, Y. Musienko, S. Nahn, C. Newman-Holmes, V. O’Dell, O. Prokofyev, E. Sexton-Kennedy, S. Sharma, A. Soha, W. J. Spalding, L. Spiegel, L. Taylor, S. Tkaczyk, N. V. Tran, L. Uplegger, E. W. Vaandering, R. Vidal, A. Whitbeck, J. Whitmore, F. Yang, D. Acosta, P. Avery, D. Bourilkov, M. Carver, T. Cheng, D. Curry, S. Das, M. De Gruttola, G. P. Di Giovanni, R. D. Field, M. Fisher, I. K. Furic, J. Hugon, J. Konigsberg, A. Korytov, T. Kypreos, J. F. Low, K. Matchev, P. Milenovic, G. Mitselmakher, L. Muniz, A. Rinkevicius, L. Shchutska, N. Skhirtladze, M. Snowball, J. Yelton, M. Zakaria, S. Hewamanage, S. Linn, P. Markowitz, G. Martinez, J. L. Rodriguez, T. Adams, A. Askew, J. Bochenek, B. Diamond, J. Haas, S. Hagopian, V. Hagopian, K. F. Johnson, H. Prosper, V. Veeraraghavan, M. Weinberg, M. M. Baarmand, M. Hohlmann, H. Kalakhety, F. Yumiceva, M. R. Adams, L. Apanasevich, V. E. Bazterra, D. Berry, R. R. Betts, I. Bucinskaite, R. Cavanaugh, O. Evdokimov, L. Gauthier, C. E. Gerber, D. J. Hofman, S. Khalatyan, P. Kurt, D. H. Moon, C. O’Brien, C. Silkworth, P. Turner, N. Varelas, E. A. Albayrak, B. Bilki, W. Clarida, K. Dilsiz, F. Duru, M. Haytmyradov, J.-P. Merlo, H. Mermerkaya, A. Mestvirishvili, A. Moeller, J. Nachtman, H. Ogul, Y. Onel, F. Ozok, A. Penzo, R. Rahmat, S. Sen, P. Tan, E. Tiras, J. Wetzel, T. Yetkin, K. Yi, B. A. Barnett, B. Blumenfeld, S. Bolognesi, D. Fehling, A. V. Gritsan, P. Maksimovic, C. Martin, M. Swartz, P. Baringer, A. Bean, G. Benelli, C. Bruner, J. Gray, R. P. Kenny, M. Murray, D. Noonan, S. Sanders, J. Sekaric, R. Stringer, Q. Wang, J. S. Wood, A. F. Barfuss, I. Chakaberia, A. Ivanov, S. Khalil, M. Makouski, Y. Maravin, L. K. Saini, S. Shrestha, I. Svintradze, J. Gronberg, D. Lange, F. Rebassoo, D. Wright, A. Baden, B. Calvert, S. C. Eno, J. A. Gomez, N. J. Hadley, R. G. Kellogg, T. Kolberg, Y. Lu, M. Marionneau, A. C. Mignerey, K. Pedro, A. Skuja, M. B. Tonjes, S. C. Tonwar, A. Apyan, R. Barbieri, G. Bauer, W. Busza, I. A. Cali, M. Chan, L. Di Matteo, V. Dutta, G. Gomez Ceballos, M. Goncharov, D. Gulhan, M. Klute, Y. S. Lai, Y.-J. Lee, A. Levin, P. D. Luckey, T. Ma, C. Paus, D. Ralph, C. Roland, G. Roland, G. S. F. Stephans, F. Stöckli, K. Sumorok, D. Velicanu, J. Veverka, B. Wyslouch, M. Yang, A. S. Yoon, M. Zanetti, V. Zhukova, B. Dahmes, A. De Benedetti, A. Gude, S. C. Kao, K. Klapoetke, Y. Kubota, J. Mans, N. Pastika, R. Rusack, A. Singovsky, N. Tambe, J. Turkewitz, J. G. Acosta, L. M. Cremaldi, R. Kroeger, S. Oliveros, L. Perera, D. A. Sanders, D. Summers, E. Avdeeva, K. Bloom, S. Bose, D. R. Claes, A. Dominguez, R. Gonzalez Suarez, J. Keller, D. Knowlton, I. Kravchenko, J. Lazo-Flores, S. Malik, F. Meier, G. R. Snow, J. Dolen, A. Godshalk, I. Iashvili, S. Jain, A. Kharchilava, A. Kumar, S. Rappoccio, G. Alverson, E. Barberis, D. Baumgartel, M. Chasco, J. Haley, A. Massironi, D. Nash, T. Orimoto, D. Trocino, D. Wood, J. Zhang, A. Anastassov, K. A. Hahn, A. Kubik, L. Lusito, N. Mucia, N. Odell, B. Pollack, A. Pozdnyakov, M. Schmitt, S. Stoynev, K. Sung, M. Velasco, S. Won, A. Brinkerhoff, K. M. Chan, A. Drozdetskiy, M. Hildreth, C. Jessop, D. J. Karmgard, N. Kellams, K. Lannon, W. Luo, S. Lynch, N. Marinelli, T. Pearson, M. Planer, R. Ruchti, N. Valls, M. Wayne, M. Wolf, A. Woodard, L. Antonelli, J. Brinson, B. Bylsma, L. S. Durkin, S. Flowers, C. Hill, R. Hughes, K. Kotov, T. Y. Ling, D. Puigh, M. Rodenburg, G. Smith, C. Vuosalo, B. L. Winer, H. Wolfe, H. W. Wulsin, E. Berry, O. Driga, P. Elmer, P. Hebda, A. Hunt, S. A. Koay, P. Lujan, D. Marlow, T. Medvedeva, M. Mooney, J. Olsen, P. Piroué, X. Quan, H. Saka, D. Stickland, C. Tully, J. S. Werner, S. C. Zenz, A. Zuranski, E. Brownson, H. Mendez, J. E. Ramirez Vargas, E. Alagoz, V. E. Barnes, D. Benedetti, G. Bolla, D. Bortoletto, M. De Mattia, Z. Hu, M. K. Jha, M. Jones, K. Jung, M. Kress, N. Leonardo, D. Lopes Pegna, V. Maroussov, P. Merkel, D. H. Miller, N. Neumeister, B. C. Radburn-Smith, X. Shi, I. Shipsey, D. Silvers, A. Svyatkovskiy, F. Wang, W. Xie, L. Xu, H. D. Yoo, J. Zablocki, Y. Zheng, N. Parashar, J. Stupak, A. Adair, B. Akgun, K. M. Ecklund, F. J. M. Geurts, W. Li, B. Michlin, B. P. Padley, R. Redjimi, J. Roberts, J. Zabel, B. Betchart, A. Bodek, R. Covarelli, P. de Barbaro, R. Demina, Y. Eshaq, T. Ferbel, A. Garcia-Bellido, P. Goldenzweig, J. Han, A. Harel, A. Khukhunaishvili, D. C. Miner, G. Petrillo, D. Vishnevskiy, R. Ciesielski, L. Demortier, K. Goulianos, G. Lungu, C. Mesropian, S. Arora, A. Barker, J. P. Chou, C. Contreras-Campana, E. Contreras-Campana, D. Duggan, D. Ferencek, Y. Gershtein, R. Gray, E. Halkiadakis, D. Hidas, A. Lath, S. Panwalkar, M. Park, R. Patel, V. Rekovic, S. Salur, S. Schnetzer, C. Seitz, S. Somalwar, R. Stone, S. Thomas, P. Thomassen, M. Walker, K. Rose, S. Spanier, A. York, O. Bouhali, R. Eusebi, W. Flanagan, J. Gilmore, T. Kamon, V. Khotilovich, V. Krutelyov, R. Montalvo, I. Osipenkov, Y. Pakhotin, A. Perloff, J. Roe, A. Rose, A. Safonov, T. Sakuma, I. Suarez, A. Tatarinov, N. Akchurin, C. Cowden, J. Damgov, C. Dragoiu, P. R. Dudero, J. Faulkner, K. Kovitanggoon, S. Kunori, S. W. Lee, T. Libeiro, I. Volobouev, E. Appelt, A. G. Delannoy, S. Greene, A. Gurrola, W. Johns, C. Maguire, Y. Mao, A. Melo, M. Sharma, P. Sheldon, B. Snook, S. Tuo, J. Velkovska, M. W. Arenton, S. Boutle, B. Cox, B. Francis, J. Goodell, R. Hirosky, A. Ledovskoy, H. Li, C. Lin, C. Neu, J. Wood, S. Gollapinni, R. Harr, P. E. Karchin, C. Kottachchi Kankanamge Don, P. Lamichhane, J. Sturdy, D. A. Belknap, D. Carlsmith, M. Cepeda, S. Dasu, S. Duric, E. Friis, R. Hall-Wilton, M. Herndon, A. Hervé, P. Klabbers, A. Lanaro, C. Lazaridis, A. Levine, R. Loveless, A. Mohapatra, I. Ojalvo, T. Perry, G. A. Pierro, G. Polese, I. Ross, T. Sarangi, A. Savin, W. H. Smith, N. Woods

**Affiliations:** 1 Yerevan Physics Institute, Yerevan, Armenia; 2 Institut für Hochenergiephysik der OeAW, Wien, Austria; 3 National Centre for Particle and High Energy Physics, Minsk, Belarus; 4 Universiteit Antwerpen, Antwerpen, Belgium; 5 Vrije Universiteit Brussel, Brussel, Belgium; 6 Université Libre de Bruxelles, Bruxelles, Belgium; 7 Ghent University, Ghent, Belgium; 8 Université Catholique de Louvain, Louvain-la-Neuve, Belgium; 9 Université de Mons, Mons, Belgium; 10 Centro Brasileiro de Pesquisas Fisicas, Rio de Janeiro, Brazil; 11 Universidade do Estado do Rio de Janeiro, Rio de Janeiro, Brazil; 12 Universidade Estadual Paulista , Universidade Federal do ABC, São Paulo, Brazil; 13 Institute for Nuclear Research and Nuclear Energy, Sofia, Bulgaria; 14 University of Sofia, Sofia, Bulgaria; 15 Institute of High Energy Physics, Beijing, China; 16 State Key Laboratory of Nuclear Physics and Technology, Peking University, Beijing, China; 17 Universidad de Los Andes, Bogota, Colombia; 18 Technical University of Split, Split, Croatia; 19 University of Split, Split, Croatia; 20 Institute Rudjer Boskovic, Zagreb, Croatia; 21 University of Cyprus, Nicosia, Cyprus; 22 Charles University, Prague, Czech Republic; 23 Academy of Scientific Research and Technology of the Arab Republic of Egypt, Egyptian Network of High Energy Physics, Cairo, Egypt; 24 National Institute of Chemical Physics and Biophysics, Tallinn, Estonia; 25 Department of Physics, University of Helsinki, Helsinki, Finland; 26 Helsinki Institute of Physics, Helsinki, Finland; 27 Lappeenranta University of Technology, Lappeenranta, Finland; 28 DSM/IRFU, CEA/Saclay, Gif-sur-Yvette, France; 29 Laboratoire Leprince-Ringuet, Ecole Polytechnique, IN2P3-CNRS, Palaiseau, France; 30 Institut Pluridisciplinaire Hubert Curien, Université de Strasbourg, Université de Haute Alsace Mulhouse, CNRS/IN2P3, Strasbourg, France; 31 Centre de Calcul de l’Institut National de Physique Nucleaire et de Physique des Particules, CNRS/IN2P3, Villeurbanne, France; 32 Institut de Physique Nucléaire de Lyon, Université de Lyon, Université Claude Bernard Lyon 1, CNRS-IN2P3, Villeurbanne, France; 33 Institute of High Energy Physics and Informatization, Tbilisi State University, Tbilisi, Georgia; 34 RWTH Aachen University, I. Physikalisches Institut, Aachen, Germany; 35 RWTH Aachen University, III. Physikalisches Institut A, Aachen, Germany; 36 RWTH Aachen University, III. Physikalisches Institut B, Aachen, Germany; 37 Deutsches Elektronen-Synchrotron, Hamburg, Germany; 38 University of Hamburg, Hamburg, Germany; 39 Institut für Experimentelle Kernphysik, Karlsruhe, Germany; 40 Institute of Nuclear and Particle Physics (INPP), NCSR Demokritos, Aghia Paraskevi, Greece; 41 University of Athens, Athens, Greece; 42 University of Ioánnina, Ioánnina, Greece; 43 Wigner Research Centre for Physics, Budapest, Hungary; 44 Institute of Nuclear Research ATOMKI, Debrecen, Hungary; 45 University of Debrecen, Debrecen, Hungary; 46 National Institute of Science Education and Research, Bhubaneswar, India; 47 Panjab University, Chandigarh, India; 48 University of Delhi, Delhi, India; 49 Saha Institute of Nuclear Physics, Kolkata, India; 50 Bhabha Atomic Research Centre, Mumbai, India; 51 Tata Institute of Fundamental Research, Mumbai, India; 52 Institute for Research in Fundamental Sciences (IPM), Tehran, Iran; 53 University College Dublin, Dublin, Ireland; 54 INFN Sezione di Bari , Università di Bari , Politecnico di Bari, Bari, Italy; 55 INFN Sezione di Bologna , Università di Bologna, Bologna, Italy; 56 INFN Sezione di Catania , Università di Catania , CSFNSM, Catania, Italy; 57 INFN Sezione di Firenze , Università di Firenze, Firenze, Italy; 58 INFN Laboratori Nazionali di Frascati, Frascati, Italy; 59 INFN Sezione di Genova , Università di Genova , Genova, Italy; 60 INFN Sezione di Milano-Bicocca , Università di Milano-Bicocca , Milano, Italy; 61 INFN Sezione di Napoli , Università di Napoli ’Federico II’ , Università della Basilicata (Potenza) , Università G. Marconi (Roma), Napoli, Italy; 62 INFN Sezione di Padova , Università di Padova , Università di Trento (Trento), Padova, Italy; 63 INFN Sezione di Pavia , Università di Pavia , Pavia, Italy; 64 INFN Sezione di Perugia , Università di Perugia , Perugia, Italy; 65 INFN Sezione di Pisa , Università di Pisa , Scuola Normale Superiore di Pisa , Pisa, Italy; 66 INFN Sezione di Roma , Università di Roma , Roma, Italy; 67 INFN Sezione di Torino , Università di Torino , Università del Piemonte Orientale (Novara) , Torino, Italy; 68 INFN Sezione di Trieste , Università di Trieste , Trieste, Italy; 69 Kangwon National University, Chunchon, Korea; 70 Kyungpook National University, Daegu, Korea; 71 Chonnam National University, Institute for Universe and Elementary Particles, Kwangju, Korea; 72 Korea University, Seoul, Korea; 73 University of Seoul, Seoul, Korea; 74 Sungkyunkwan University, Suwon, Korea; 75 Vilnius University, Vilnius, Lithuania; 76 National Centre for Particle Physics, Universiti Malaya, Kuala Lumpur, Malaysia; 77 Centro de Investigacion y de Estudios Avanzados del IPN, Mexico City, Mexico; 78 Universidad Iberoamericana, Mexico City, Mexico; 79 Benemerita Universidad Autonoma de Puebla, Puebla, Mexico; 80 Universidad Autónoma de San Luis Potosí, San Luis Potosí, Mexico; 81 University of Auckland, Auckland, New Zealand; 82 University of Canterbury, Christchurch, New Zealand; 83 National Centre for Physics, Quaid-I-Azam University, Islamabad, Pakistan; 84 National Centre for Nuclear Research, Swierk, Poland; 85 Institute of Experimental Physics, Faculty of Physics, University of Warsaw, Warsaw, Poland; 86 Laboratório de Instrumentação e Física Experimental de Partículas, Lisboa, Portugal; 87 Joint Institute for Nuclear Research, Dubna, Russia; 88 Petersburg Nuclear Physics Institute, Gatchina (St. Petersburg), Russia; 89 Institute for Nuclear Research, Moscow, Russia; 90 Institute for Theoretical and Experimental Physics, Moscow, Russia; 91 P.N. Lebedev Physical Institute, Moscow, Russia; 92 Skobeltsyn Institute of Nuclear Physics, Lomonosov Moscow State University, Moscow, Russia; 93 State Research Center of Russian Federation, Institute for High Energy Physics, Protvino, Russia; 94 University of Belgrade, Faculty of Physics and Vinca Institute of Nuclear Sciences, Belgrade, Serbia; 95 Centro de Investigaciones Energéticas Medioambientales y Tecnológicas (CIEMAT), Madrid, Spain; 96 Universidad Autónoma de Madrid, Madrid, Spain; 97 Universidad de Oviedo, Oviedo, Spain; 98 Instituto de Física de Cantabria (IFCA), CSIC-Universidad de Cantabria, Santander, Spain; 99 CERN, European Organization for Nuclear Research, Geneva, Switzerland; 100 Paul Scherrer Institut, Villigen, Switzerland; 101 Institute for Particle Physics, ETH Zurich, Zurich, Switzerland; 102 Universität Zürich, Zurich, Switzerland; 103 National Central University, Chung-Li, Taiwan; 104 National Taiwan University (NTU), Taipei, Taiwan; 105 Chulalongkorn University, Bangkok, Thailand; 106 Cukurova University, Adana, Turkey; 107 Physics Department, Middle East Technical University, Ankara, Turkey; 108 Bogazici University, Istanbul, Turkey; 109 Istanbul Technical University, Istanbul, Turkey; 110 National Scientific Center, Kharkov Institute of Physics and Technology, Kharkov, Ukraine; 111 University of Bristol, Bristol, UK; 112 Rutherford Appleton Laboratory, Didcot, UK; 113 Imperial College, London, UK; 114 Brunel University, Uxbridge, UK; 115 Baylor University, Waco, USA; 116 The University of Alabama, Tuscaloosa, USA; 117 Boston University, Boston, USA; 118 Brown University, Providence, USA; 119 University of California, Davis, USA; 120 University of California, Los Angeles, USA; 121 University of California, Riverside, Riverside, USA; 122 University of California, San Diego, La Jolla, USA; 123 University of California, Santa Barbara, Santa Barbara, USA; 124 California Institute of Technology, Pasadena, USA; 125 Carnegie Mellon University, Pittsburgh, USA; 126 University of Colorado at Boulder, Boulder, USA; 127 Cornell University, Ithaca, USA; 128 Fairfield University, Fairfield, USA; 129 Fermi National Accelerator Laboratory, Batavia, USA; 130 University of Florida, Gainesville, USA; 131 Florida International University, Miami, USA; 132 Florida State University, Tallahassee, USA; 133 Florida Institute of Technology, Melbourne, USA; 134 University of Illinois at Chicago (UIC), Chicago, USA; 135 The University of Iowa, Iowa City, USA; 136 Johns Hopkins University, Baltimore, USA; 137 The University of Kansas, Lawrence, USA; 138 Kansas State University, Manhattan, USA; 139 Lawrence Livermore National Laboratory, Livermore, USA; 140 University of Maryland, College Park, USA; 141 Massachusetts Institute of Technology, Cambridge, USA; 142 University of Minnesota, Minneapolis, USA; 143 University of Mississippi, Oxford, USA; 144 University of Nebraska-Lincoln, Lincoln, USA; 145 State University of New York at Buffalo, Buffalo, USA; 146 Northeastern University, Boston, USA; 147 Northwestern University, Evanston, USA; 148 University of Notre Dame, Notre Dame, USA; 149 The Ohio State University, Columbus, USA; 150 Princeton University, Princeton, USA; 151 University of Puerto Rico, Mayaguez, USA; 152 Purdue University, West Lafayette, USA; 153 Purdue University Calumet, Hammond, USA; 154 Rice University, Houston, USA; 155 University of Rochester, Rochester, USA; 156 The Rockefeller University, New York, USA; 157 Rutgers, The State University of New Jersey, Piscataway, USA; 158 University of Tennessee, Knoxville, USA; 159 Texas A&M University, College Station, USA; 160 Texas Tech University, Lubbock, USA; 161 Vanderbilt University, Nashville, USA; 162 University of Virginia, Charlottesville, USA; 163 Wayne State University, Detroit, USA; 164 University of Wisconsin, Madison, USA; 165 CERN, Geneva, Switzerland

## Abstract

Searches for the direct electroweak production of supersymmetric charginos, neutralinos, and sleptons in a variety of signatures with leptons and $\mathrm{W}$, $\mathrm{Z}$, and Higgs bosons are presented. Results are based on a sample of proton-proton collision data collected at center-of-mass energy $\sqrt{s}=8\,\mathrm{TeV}$ with the CMS detector in 2012, corresponding to an integrated luminosity of 19.5 $\,\text {fb}^\text {-1}$. The observed event rates are in agreement with expectations from the standard model. These results probe charginos and neutralinos with masses up to 720 $\,\text {GeV}$, and sleptons up to 260 $\,\text {GeV}$, depending on the model details.

## Introduction

Many searches for supersymmetry (SUSY) [1–5] carried out at the CERN Large Hadron Collider (LHC) have focused on models with cross sections dominated by the production of strongly interacting new particles in final states with high levels of hadronic activity [6–17]. Null results from these searches constrain the squarks and gluinos to be heavier than several hundred GeV. In contrast, in this paper, we describe searches motivated by the direct electroweak production of charginos $\widetilde{\chi }^{\pm }$ and neutralinos $\widetilde{\chi }^{0}$, mixtures of the SUSY partners of the gauge and Higgs bosons, and of sleptons $\widetilde{\ell }$, the SUSY partners of leptons. These production modes may dominate at the LHC if the strongly interacting SUSY particles are heavy. The corresponding final states do not necessarily contain much hadronic activity and thus may have eluded detection.

The smaller cross sections typical of direct electroweak SUSY production require dedicated searches targeting the wide variety of possible signal topologies. Depending on the mass spectrum, the charginos and neutralinos can have significant decay branching fractions to leptons or $\mathrm{W}$, $\mathrm{Z}$, and Higgs bosons (H), yielding final states with at least one isolated lepton. Similarly, slepton pair production gives rise to final states with two leptons. In all these cases, and under the assumption of R-parity conservation [5], two stable, lightest SUSY particles (LSP) are produced, which are presumed to escape without detection, leading to significant missing transverse energy $E_{\mathrm {T}}^{\text {miss}}$. We thus search for SUSY in a variety of final states with one or more leptons and $E_{\mathrm {T}}^{\text {miss}}$.

**Fig. 1:**
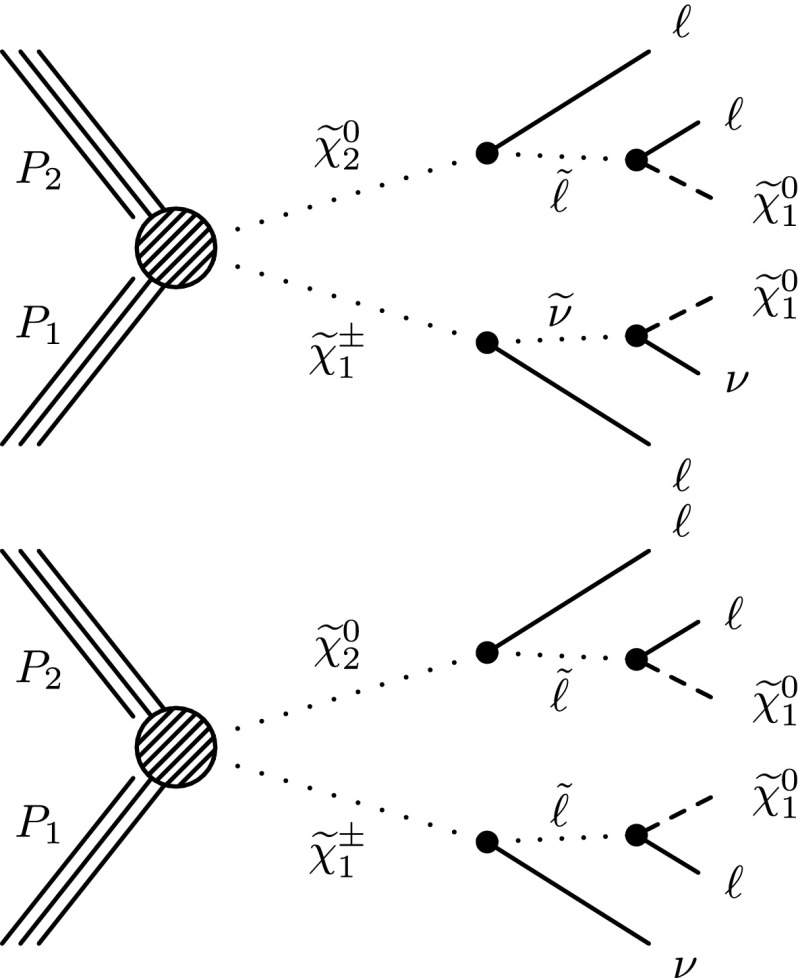
Chargino–neutralino pair production with decays mediated by sleptons and sneutrinos, leading to a three-lepton final state with missing transverse energy $E_{\mathrm {T}}^{\text {miss}}$

**Fig. 2:**
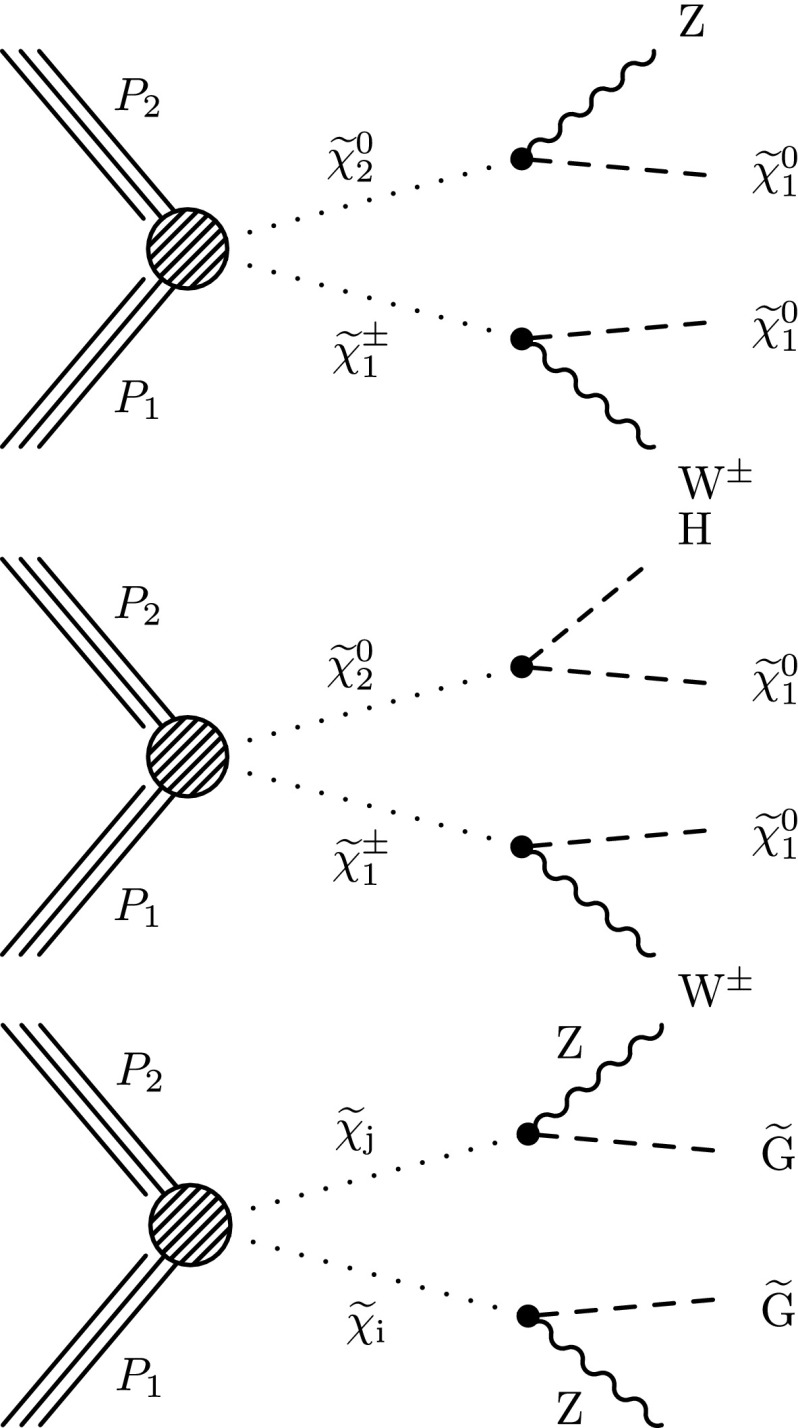
Chargino–neutralino production, with the chargino decaying to a $\mathrm{W}$ boson and the LSP, and with the neutralino decaying to (*top*) a $\mathrm{Z}$ boson and the LSP or (*center*) a Higgs boson and the LSP (*bottom*) a GMSB model with higgsino pair production, with $\tilde{\chi }_i$ and $\tilde{\chi }_j$ indicating nearly mass-degenerate charginos and neutralinos, leading to the $\mathrm{Z}\mathrm{Z}+E_{\mathrm {T}}^{\text {miss}}$ final state

**Fig. 3:**
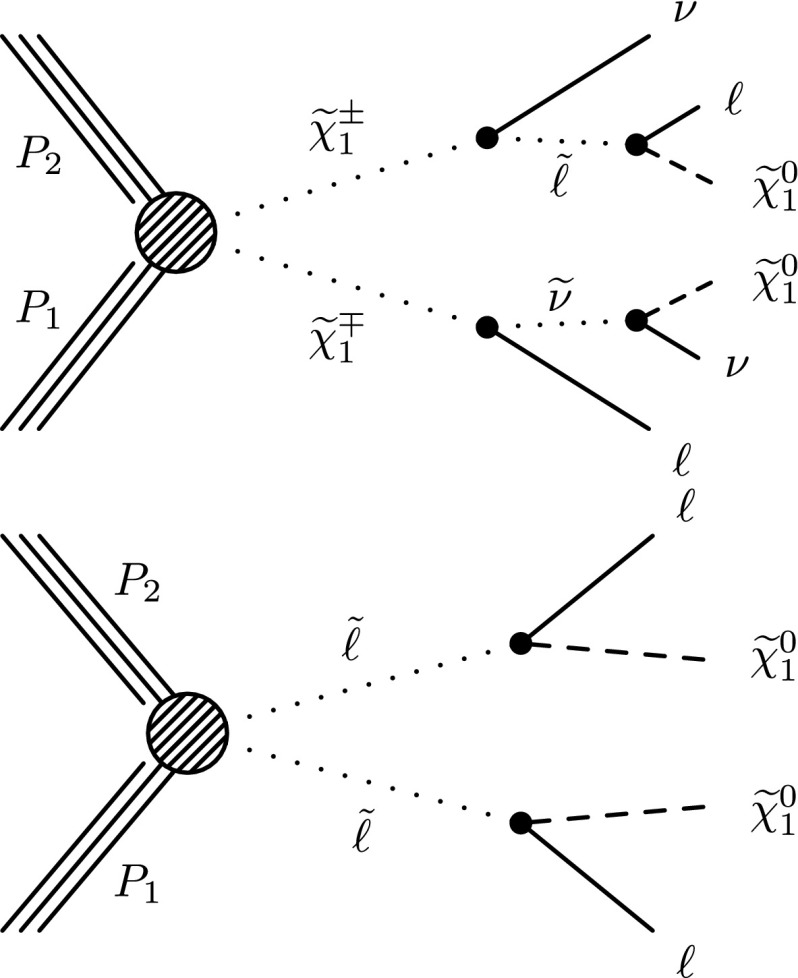
Chargino(*top*) , and slepton (*bottom*) pair production leading to opposite-sign lepton pairs with $E_{\mathrm {T}}^{\text {miss}}$

The searches are based on a sample of proton–proton (pp) collision data collected at $\sqrt{s}=8$ TeV with the Compact Muon Solenoid (CMS) detector at the LHC in 2012, corresponding to an integrated luminosity of 19.5 $\,\text {fb}^\text {-1}$. The study is an update of Reference [18], with improvements to the analysis techniques and the addition of new signal scenarios and search channels. Similar studies in the two-lepton, three-lepton, and four-lepton final states have been performed by the ATLAS Collaboration [19–21]. The new-physics scenarios we consider are shown in Figs. 1, 2 and 3. These figures are labeled using SUSY nomenclature, but the interpretation of our results can be extended to other new-physics models. In SUSY nomenclature, $\widetilde{\chi }^{0} _1$ is the lightest neutralino, presumed to be the LSP, $\widetilde{\chi }^{0} _2$ is a heavier neutralino, $\widetilde{\chi }^{\pm } _1$ is the lightest chargino, and $\widetilde{\ell }$ is a slepton. We also consider a model in which the gravitino (${\widetilde{\mathrm{G}}}$) is the LSP.

The results are interpreted considering each diagram in Figs. 1, 2 and 3 individually. The masses of the new-physics particles are treated as independent parameters. SUSY models with a bino-like $\widetilde{\chi }^{0} _1$ and wino-like $\widetilde{\chi }^{0} _2$ and $\widetilde{\chi }^{\pm } _1$ motivate the simplifying assumption $m_{\widetilde{\chi }}\equiv m_{\widetilde{\chi }^{\pm } _{1}}= m_{\widetilde{\chi }^{0} _{2}}$ since these two gauginos belong to the same gauge group multiplet. We thus present results as a function of the common mass $m_{\widetilde{\chi }}$ and the LSP mass $m_{\widetilde{\chi }^{0} _1}$.

In the models shown in Figs. 1 and 3 (top), the slepton mass $m_{\widetilde{\ell }}$ is less than the common mass $m_{\widetilde{\chi }}$, and the sleptons are produced in the decay chains of the charginos and neutralinos. The results in these scenarios also depend on the mass $m_{\widetilde{\ell }}$ of the intermediate slepton (if left-handed, taken to be the same for its sneutrino $\widetilde{\nu }$), parametrized in terms of a variable $x_{\widetilde{\ell }}$ as:

1 $$\begin{aligned} m_{\widetilde{\ell }} = m_{\widetilde{\nu }} = m_{\widetilde{\chi }^{0} _1} + x_{\widetilde{\ell }} \, ( m_{\widetilde{\chi }}- m_{\widetilde{\chi }^{0} _1}\,), \end{aligned}$$

where $0<x_{\widetilde{\ell }} <1$. We present results for $x_{\widetilde{\ell }} =0.50$, i.e., the slepton mass equal to the mean of the LSP and the $\widetilde{\chi }$ masses, and in some cases for more compressed spectra with $x_{\widetilde{\ell }} =0.05$ or 0.95, i.e., the slepton mass close to either the LSP or the $\widetilde{\chi }$ mass, respectively.

For the models in Fig. 2, we assume that sleptons are so massive that diagrams containing virtual or real sleptons in the chargino or neutralino decay process can be ignored. In Fig. 2 (top and center), the chargino decays to a $\mathrm{W}$ boson and the LSP, while the neutralino may decay either to a $\mathrm{Z}$ or $\mathrm{H}$ boson and the LSP, with branching fractions that depend on model details. The $\mathrm{H}$ boson is identified with the lightest neutral CP-even state of extended Higgs sectors. The $\mathrm{H}$ boson is expected to have SM Higgs boson properties if all other Higgs bosons are much heavier [22]. We thus search in both the $\mathrm{W}\mathrm{Z}+E_{\mathrm {T}}^{\text {miss}}$ and $\mathrm{W}\mathrm{H}+E_{\mathrm {T}}^{\text {miss}}$ signatures. There is little sensitivity to the ${ZZ}$ channel of Fig. 2 (bottom) if the $\widetilde{\chi }^{0} _2$ and $\widetilde{\chi }^{\pm } _1$ are wino-like, in which case neutralino pair production is suppressed relative to neutralino–chargino production. Therefore, for the ${ZZ}$ signature, we consider a specific gauge-mediated supersymmetry breaking (GMSB) model with higgsino next-to-lightest SUSY particles (NLSP) and a gravitino LSP [23–25], which enhances the ${ZZ}+ E_{\mathrm {T}}^{\text {miss}}$ production rate. In this model, the $\widetilde{\chi }^{0} _2$ and $\widetilde{\chi }^{\pm } _1$ particles are nearly mass degenerate with the $\widetilde{\chi }^{0} _1$ NLSP, and each decay to the $\widetilde{\chi }^{0} _1$ through the emission of low-$p_{\mathrm {T}}$, undetected SM particles. The $\widetilde{\chi }^{0} _1$ then decays to a $\mathrm{Z}$ boson and the gravitino LSP. The production of the $\mathrm{H}\mathrm{H}+E_{\mathrm {T}}^{\text {miss}}$ and $\mathrm{Z}\mathrm{H}+E_{\mathrm {T}}^{\text {miss}}$ final states is also possible in the GMSB model, depending on the character of the NLSP. These latter two final states are not considered in the current study.

Figure 3 (top) depicts chargino pair production. For this process, each chargino can decay via either of the two modes shown. Thus, there are four different decay pairs, but all yield a similar final state, with two opposite-sign leptons and $E_{\mathrm {T}}^{\text {miss}}$. For this model, we consider $x_{\widetilde{\ell }} =0.5$ only. Figure 3(bottom) illustrates slepton pair production, where each slepton decays to a lepton of the same flavor and to the LSP. We consider left- and right-handed slepton production separately, and assume a universal mass for both the selectron and smuon. The results of this analysis are not sensitive to the direct production of $\tau$-slepton pairs.

This paper is organized as follows. In Sect. 2, we describe the detector, data and simulated samples, and event reconstruction procedures. Section 3 presents a search based on the three-lepton final states of Figs. 1 and 2 (top). A search based on the four-lepton final state, which is sensitive to the diagram of Fig. 2 (bottom), is presented in Sect. 4. Section 5 describes a search in a channel with exactly two same-sign dileptons, which enhances sensitivity to the diagrams of Fig. 1 in cases where one of the three leptons is not identified. In Sect. 6 we present a search based on the $\mathrm{W}\mathrm{Z}/\mathrm{Z}\mathrm{Z}+E_{\mathrm {T}}^{\text {miss}}$ signature, which is sensitive to the diagrams shown in Fig. 2 (top and bottom). Section 7 presents a set of searches targeting $\mathrm{W}\mathrm{H}+E_{\mathrm {T}}^{\text {miss}}$ production in the single-lepton, same-sign dilepton, and three-or-more-lepton channels, probing the diagram of Fig. 2 (center). In Sect. 8, we present a search based on an opposite-sign, non-resonant dilepton pair (electrons and muons), which is sensitive to the processes of Fig. 3. Section 9 presents interpretations of these searches and Sect. 10 a summary.

## Detector, trigger, and physics object selection

The central feature of the CMS apparatus is a superconducting solenoid, of 6 $\text {m}$ internal diameter, providing a magnetic field of 3.8 $\text {T}$. Within the field volume are a silicon pixel and strip tracker, a crystal electromagnetic calorimeter, and a brass-scintillator hadron calorimeter. Muons are measured with gas-ionization detectors embedded in the steel flux-return yoke of the solenoid. A detailed description can be found in Reference [26].

The origin of the coordinate system is the nominal interaction point. The $x$ axis points to the center of the LHC ring and the $y$ axis vertically upwards. The $z$ axis lies in the direction of the counterclockwise proton beam. The polar angle $\theta$ is measured from the positive $z$ axis, and the azimuthal angle $\phi$ (in radians) is measured in the $x$–$y$ plane. The pseudorapidity $\eta$ is defined by $\eta = -\ln [\tan (\theta /2)]$.

Events from pp interactions must satisfy the requirements of a two-level trigger system. The first level performs a fast selection of physics objects (jets, muons, electrons, and photons) above certain thresholds. The second level performs a full event reconstruction. The principal trigger used for the searches with two or more leptons is a dilepton trigger. It requires at least one electron or muon with transverse momentum $p_{\mathrm {T}} >17$ GeV and another with $p_{\mathrm {T}} >8$ GeV. The trigger used for the single-lepton final state requires a single electron (muon) with $p_{\mathrm {T}} >27\,(24)$ GeV. All leptons must satisfy $|\eta |<2.4$.

Simulated event samples are used to study the characteristics of signal and standard model (SM) background processes, using the CTEQ6L1 [27] parton distribution functions. The main backgrounds are from top-quark pair (${\mathrm{t}}\overline{{\mathrm{t}}}$), diboson, $\mathrm{Z}+\text {jets}$, and $\mathrm{W}+\text {jets}$ processes, depending on the channel considered. Most of the simulated SM background samples are produced with the MadGraph 5.1.5.4 [28] event generator, with parton showering and hadronization performed with the pythia 6.4.26 [29] program. We use the most accurate calculations of the cross sections available, generally with next-to-leading-order (NLO) accuracy [30–32]. The detector response is modeled with the Geant4  [33] library, followed by the same event reconstruction as used for data.

Signal samples are generated with the MadGraph 5.1.5.4 generator including up to two additional partons at the matrix element level. Parton showering, hadronization, and the decay of particles, including SUSY particles, are described with the pythia 6.4.26 [29] program. Signal cross sections are calculated at NLO+NLL using the Resummino [34–36] calculation, where NLL refers to the next-to-leading-logarithmic precision. For the SUSY samples with a Higgs boson ($\mathrm{H}$) in the final state, a mass of $m_\mathrm{H}= 126$ GeV [37] is assumed, along with SM branching fractions. Here the $\mathrm{H}$ particle indicates the lightest neutral CP-even SUSY Higgs boson, which is expected to have SM-like properties if the other SUSY Higgs bosons are much heavier [22]. To reduce computational requirements, the simulation of detector response for signal samples is based on the CMS fast simulation program [38] in place of Geant4.

Events are reconstructed using the particle-flow (PF) algorithm [39, 40], which provides a self-consistent global assignment of momenta and energies to the physics objects. Details of the reconstruction and identification procedures for electrons, muons, and photons are given in References [41–43]. Lepton ($\mathrm {e}, \mu$) candidates are required to be consistent with the primary event vertex, defined as the vertex with the largest value of $\Sigma (p_{\mathrm {T}} ^{\text {track}})^2$, where the summation includes all tracks associated to a given vertex. In the searches with two or more leptons, events with an opposite-sign $\mathrm {e}\mathrm {e}$, $\mu \mu$, or $\mathrm {e}\mu$ pair with an invariant mass below 12 GeV are rejected in order to exclude quarkonia resonances, photon conversions, and low-mass continuum events. To reduce contamination due to leptons from heavy-flavor decay or misidentified hadrons in jets, leptons are required to be isolated and to have a transverse impact parameter with respect to the primary vertex satisfying $d_0 < 0.2$ $\text {mm}$. Electron and muon candidates are considered isolated if the ratio $I_\text {rel}$ of the scalar sum of the transverse momenta of charged hadrons, photons, and neutral hadrons in a cone of $\Delta R = \sqrt{{(\Delta \eta )^2+(\Delta \phi )^2}}=0.3$ around the candidate, divided by the lepton $p_{\mathrm {T}}$ value, is less than 0.15. The requirements on the $d_0$ and $I_\text {rel}$ variables are more stringent in the searches utilizing same-sign dileptons and are described in Sect. 5.

The “hadrons-plus-strips” algorithm [44], which combines PF photon and electron candidates to form neutral pions, and then the neutral pions with charged hadrons, is used to identify hadronically decaying $\tau$-lepton candidates ($\tau _\mathrm {h}$).

Jets are reconstructed with the anti-$k_{\mathrm {T}}$ clustering algorithm [45] with a distance parameter of 0.5. We apply $p_{\mathrm {T}}$- and $\eta$-dependent corrections to account for residual effects of non-uniform detector response [46]. A correction to account for multiple pp collisions within the same or a nearby bunch crossing (pileup interactions) is estimated on an event-by-event basis using the jet-area method described in Reference [47], and is subtracted from the reconstructed jet $p_{\mathrm {T}}$. We reject jets that are consistent with anomalous noise in the calorimeters [48]. Jets must satisfy $|\eta | < 2.5$ and $p_{\mathrm {T}} > 30$ GeV and be separated by $\Delta R > 0.4$ from lepton candidates. The searches presented below make use of the missing transverse energy $E_{\mathrm {T}}^{\text {miss}}$, where $E_{\mathrm {T}}^{\text {miss}}$ is defined as the modulus of the vector sum of the transverse momenta of all PF objects. The $E_{\mathrm {T}}^{\text {miss}}$ vector is the negative of that same vector sum. Similarly, some of the searches use the quantity $H_{\mathrm {T}}$, defined as the scalar sum of jet $p_{\mathrm {T}}$ values.

Most signal topologies considered do not have jets from bottom quarks (“$\mathrm{b}\ \text {jets}$”); for these topologies, events containing $\mathrm{b}\ \text {jets}$ are rejected to reduce the background from ${\mathrm{t}}\overline{{\mathrm{t}}}$ production. Jets originating from b quarks are identified using the combined secondary vertex algorithm (CSV) [49]. Unless otherwise stated, we use the “medium” working point, denoted CSVM, which has an average $\mathrm{b}\text {-jet}$ tagging efficiency of 70 %, light-quark jet misidentification rate of 1.5 %, and $\text {c}$-quark jet misidentification rate of 20 % for jets with a $p_{\mathrm {T}}$ value greater than 60 GeV. Corrections are applied to simulated samples to match the expected efficiencies and misidentification rates measured in data. With the exception of the searches described in Sects. 5 and 7, the searches reject events containing CSVM-identified $\mathrm{b}\ \text {jets}$ with $p_{\mathrm {T}} >30$ GeV.

## Search in the three-lepton final state

Three-lepton channels have sensitivity to models with signatures like those shown in Figs. 1 and 2. For the three-lepton search, we use reconstructed electrons, muons, and $\tau _\mathrm {h}$ leptons, all within $|\eta |<2.4$, requiring that there be exactly three leptons in an event. There must be at least one electron or muon with $p_{\mathrm {T}} > 20$ GeV. Other electrons or muons must have $p_{\mathrm {T}} > 10$ GeV. At most one $\tau _\mathrm {h}$ candidate is allowed and it must have $p_{\mathrm {T}} > 20$ GeV. Events with multiple $\tau _\mathrm {h}$ leptons have large backgrounds and are not considered in the present analysis. The principal backgrounds are from $\mathrm{W}\mathrm{Z}$ diboson production with three genuine isolated leptons that are “prompt” (created at the primary vertex), and from ${\mathrm{t}}\overline{{\mathrm{t}}}$ production with two genuine prompt leptons and a third non-prompt lepton that is misclassified as prompt.

Events are required to have $E_{\mathrm {T}}^{\text {miss}} > 50$ GeV. We consider events both with and without an opposite-sign-same-flavor (OSSF) electron or muon pair. Events with an OSSF pair are characterized by the invariant mass $M_{\ell \ell }$ of the pair and by the transverse mass $M_\mathrm {T} \equiv \sqrt{{2E_{\mathrm {T}}^{\text {miss}} p_{\mathrm {T}} ^{\ell }[1-\cos (\Delta \phi )]}}$ formed from the $E_{\mathrm {T}}^{\text {miss}}$ vector, the transverse momentum $p_{\mathrm {T}} ^{\ell }$ of the remaining lepton, and the corresponding difference $\Delta \phi$ in azimuthal angle. For the three-muon and three-electron events, the OSSF pair with $M_{\ell \ell }$ closer to the $\mathrm{Z}$ mass is used. For events without an OSSF pair, which might arise from events with a $\mathrm{Z}\rightarrow \tau \tau$ decay, $M_{\ell \ell }$ is calculated by combining opposite-sign leptons and choosing the pair closest to the corresponding mean dilepton mass determined from $\mathrm{Z}\rightarrow \tau \tau$ simulation (50 GeV for an $\mathrm {e}\mu$ pair, and 60 GeV for a $\tau _\mathrm {h} \mu$ or $\tau _\mathrm {h} \mathrm {e}$ pair).

Events are examined in exclusive search regions (“bins”) based on their values of $M_{\ell \ell }$, $E_{\mathrm {T}}^{\text {miss}}$, and $M_\mathrm {T}$, as presented below. The $M_{\ell \ell }$ regions for OSSF dilepton pairs are $M_{\ell \ell } <75$ GeV (“below-Z”), $75<M_{\ell \ell } <105$ GeV (“on-Z”), and $M_{\ell \ell } >105$ GeV (“above-Z”). Further event classification is in $E_{\mathrm {T}}^{\text {miss}}$ bins of 50–100, 100–150, 150–200, and $>$200 GeV. Finally, the $M_\mathrm {T}$ regions are $<$120, 120–160, and $>$160 GeV.

### Background estimation

The main backgrounds in this search are due to $\mathrm{W}\mathrm{Z}$ and ${\mathrm{t}}\overline{{\mathrm{t}}}$ production, while the background from events with $\mathrm{Z}+\text {jets}$ and Drell–Yan production is strongly suppressed by the requirement on $E_{\mathrm {T}}^{\text {miss}}$. The evaluation of these backgrounds is described in Sects. 3.1.1 and 3.1.2. Less important backgrounds from $\mathrm{Z}\mathrm{Z}$ production and from rare SM processes such as ${\mathrm{t}}\overline{{\mathrm{t}}}\mathrm{Z}$, ${\mathrm{t}}\overline{{\mathrm{t}}}\mathrm{W}$, ${\mathrm{t}}\overline{{\mathrm{t}}}\mathrm{H}$, and triboson production are estimated from simulation using leading-order (LO) generators and are normalized to the NLO production cross sections [50–52]. A 50 % systematic uncertainty is assigned to these backgrounds to account both for the theoretical uncertainty of the cross section calculation and for the differences of the ratio between the LO and NLO cross sections as a function of various physical observables [50].

The systematic uncertainty for backgrounds determined using data control samples is estimated from the difference between the predicted and genuine yields when the methods are applied to simulation.

#### Background due to $\mathrm{W}\mathrm{Z}$ production

The three-lepton analysis relies on the $E_{\mathrm {T}}^{\text {miss}}$ and $M_\mathrm {T}$ variables to discriminate between signal and background. The largest background is from $\mathrm{W}\mathrm{Z}$ production. For our previous study [18], based on the CMS data collected in 2011, we calibrated the hadronic recoil of the WZ system using a generalization of the Z-recoil method discussed in Reference [53]. This calibration led to corrections to the $E_{\mathrm {T}}^{\text {miss}}$ and $M_\mathrm {T}$ distributions in simulated $\mathrm{W}\mathrm{Z}$ events. For the data collected in 2012, the rate of pileup interactions increased. We therefore developed a second method, described below, designed to specifically account for jet activity and pileup. The two methods yield consistent results and have similar systematic uncertainties; hence we use the average prediction as our $\mathrm{W}\mathrm{Z}$ background estimate.

In the new method, we subdivide the $E_{\mathrm {T}}^{\text {miss}}$ distribution in a $\mathrm{Z}+\text {jets}$ sample as a function of $H_{\mathrm {T}}$ and of the number of reconstructed vertices in the event. A large number of vertices corresponds to large pileup, which causes extraneous reconstruction of energy, degrading the $E_{\mathrm {T}}^{\text {miss}}$ resolution. Larger $H_{\mathrm {T}}$ implies greater jet activity, which degrades the $E_{\mathrm {T}}^{\text {miss}}$ resolution as a consequence of the possible jet energy mismeasurement.

In a given two-dimensional bin of the number of reconstructed vertices and $H_{\mathrm {T}}$, the $x$ and $y$ components of $E_{\mathrm {T}}^{\text {miss}}$ are found to be approximately Gaussian. Therefore the $E_{\mathrm {T}}^{\text {miss}}$ distribution is expected to follow the Rayleigh distribution, given by:

2 $$\begin{aligned} p(E_{\mathrm {T}}^{\text {miss}})=\sum _{ij} W_{ij}\frac{E_{\mathrm {T}}^{\text {miss}}}{\sigma ^2_{ij}}\text {e}^{-(E_{\mathrm {T}}^{\text {miss}})^2/2\sigma ^2_{ij}}, \end{aligned}$$

where $i$ represents the number of vertices in the event, $j$ is the $H_{\mathrm {T}}$ bin number, $W_{ij}$ is the fraction of events in the bin, and $\sigma _{ij}$ characterizes the $E_{\mathrm {T}}^{\text {miss}}$ resolution. We then adjust the $\sigma _{ij}$ terms in simulation to match those found in data. The magnitude of the correction varies from a few percent to as high as 30 %. To evaluate a systematic uncertainty for this procedure, we vary the level of $E_{\mathrm {T}}^{\text {miss}}$ smearing and determine the migration between different $E_{\mathrm {T}}^{\text {miss}}$ and $M_\mathrm {T}$ bins in the simulated $\mathrm{W}\mathrm{Z}$ sample. We find the uncertainty of the $\mathrm{W}\mathrm{Z}$ background to be 20–35 %, depending on the search region. The final WZ estimate is obtained by normalizing the corrected $E_{\mathrm {T}}^{\text {miss}}$ and $M_\mathrm {T}$ shape to the theoretical cross section. The theoretical cross section is used to evaluate the SM background from WZ events because the contributions of signal events to WZ data control samples are expected to be significant.

#### Background due to non-prompt leptons

Non-prompt lepton backgrounds arise from $\mathrm{Z}+\text {jets}$, Drell–Yan, ${\mathrm{t}}\overline{{\mathrm{t}}}$, and $\mathrm{W}\mathrm{W}+\text {jets}$ events that have two genuine isolated prompt leptons. The third lepton can be a non-prompt lepton from a heavy-flavor decay that is classified as being prompt, or a hadron from a jet that is misidentified as a lepton. This background is estimated using auxiliary data samples. The probability for a non-prompt lepton to satisfy the isolation requirement ($I_\text {rel} < 0.15$) is measured in a data sample enriched with dijet events, and varies as a function of lepton $p_{\mathrm {T}}$. Alternatively, the isolation probability is studied using $\mathrm{Z}$-boson and ${\mathrm{t}}\overline{{\mathrm{t}}}$-enriched data samples. These probabilities, applied to the three-lepton events with the isolation requirement on one of the leptons inverted, are used to estimate background due to such non-prompt leptons. We average the results of the two methods taking into account the precision of each method and the correlations between the individual inputs.

#### Background due to internal conversions

Another background, estimated from data, is due to events with a $\mathrm{Z}$ boson and an initial- or final-state photon in which the photon undergoes an asymmetric internal conversion, leading to a reconstructed three-lepton state [13]. To address this background, we measure the rates of $\mathrm{Z}\rightarrow \ell ^{+} \ell ^{-} \gamma$ and $\mathrm{Z}\rightarrow \ell ^{+} \ell ^{-} \ell ^{\pm }$ events in an off-peak control region defined by $|M_{\ell \ell }- M_{\mathrm{Z}} | > 15$ GeV and $E_{\mathrm {T}}^{\text {miss}} < 50$ GeV. The background estimate is obtained by multiplying the ratio of these rates by the measured rate of events with two leptons and a photon in the search regions. Note that external conversions are strongly suppressed by our electron selection requirements.

### Three-lepton search results

**Fig. 4:**
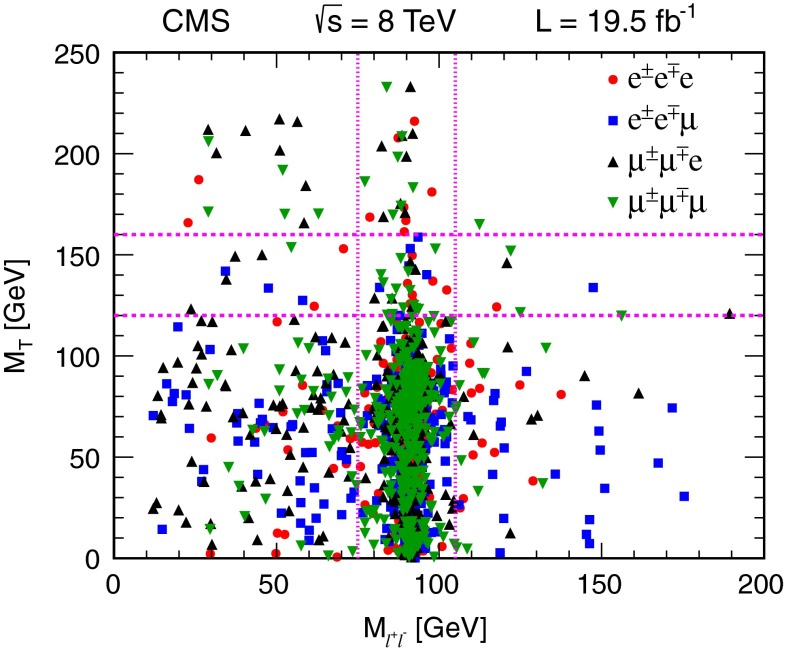
$M_\mathrm {T}$ versus $M_{\ell \ell }$ for three-lepton events in data with an $\mathrm {e}\mathrm {e}$ or $\mu \mu$ OSSF dilepton pair, where the third lepton is either an electron or a muon. Events outside of the plotted range are not indicated

**Fig. 5:**
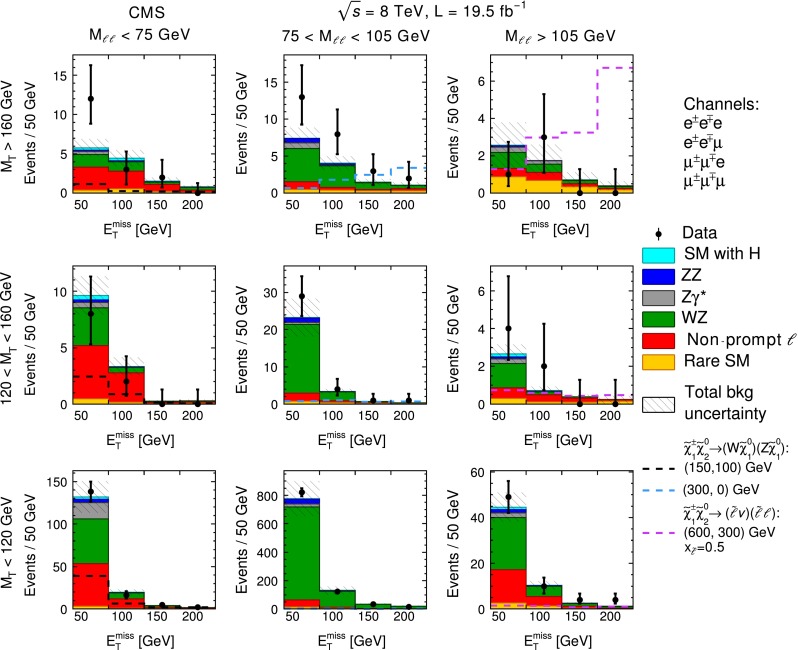
$E_{\mathrm {T}}^{\text {miss}}$ distributions, in bins of $M_\mathrm {T}$ and $M_{\ell \ell }$, for three-lepton events with an $\mathrm {e}\mathrm {e}$ or $\mu \mu$ OSSF dilepton pair, where the third lepton is either an electron or a muon. The SM expectations are also shown. The $E_{\mathrm {T}}^{\text {miss}}$ distributions for example signal scenarios are overlaid. The *first* (*second*) *number in parentheses* indicates the value of $m_{\widetilde{\chi }}$ ($m_{\widetilde{\chi }^{0} _{1}}$)

Figure 4 shows the distribution of $M_\mathrm {T}$ versus $M_{\ell \ell }$ for data events with an $\mathrm {e}\mathrm {e}$ or $\mu \mu$ OSSF pair, where the third lepton is either an electron or muon. The dashed lines delineate nine two-dimensional search regions in the $M_\mathrm {T}$–$M_{\ell \ell }$ plane. The corresponding $E_{\mathrm {T}}^{\text {miss}}$ distributions are shown in comparison to the SM expectations in Fig. 5. Table 1 lists the results as a function of $E_{\mathrm {T}}^{\text {miss}}$, $M_\mathrm {T}$, and $M_{\ell \ell }$. The data are broadly consistent with SM expectations. In the search regions with $M_\mathrm {T} >160$ GeV and an on-Z OSSF dilepton pair, and in the search region with $M_\mathrm {T} >160$ GeV, $50<E_{\mathrm {T}}^{\text {miss}} <100$ GeV, and a below-Z OSSF pair, the data exceed the expected background with a local significance at the level of approximately two standard deviations.

**Table 1:** Observed yields and SM expectations for three-lepton events with an $\mathrm {e}\mathrm {e}$ or $\mu \mu$ OSSF pair, where the third lepton is either an electron or muon. The uncertainties include both the statistical and systematic components

$M_\mathrm {T}$ ( GeVns)	$E_{\mathrm {T}}^{\text {miss}}$ (GeV)	$M_{\ell \ell } < 75$ GeV		$75 < M_{\ell \ell } < 105$ GeV		$M_{\ell \ell } > 105$ GeV	
		Total bkg	Observed	Total bkg	Observed	Total bkg	Observed
$>$160	50–100	5.8 $\pm$ 1.1	12	7.5 $\pm$ 1.4	13	2.6 $\pm$ 1.2	1
	100–150	4.5 $\pm$ 1.1	3	4.0 $\pm$ 1.0	8	1.8 $\pm$ 0.9	3
	150–200	1.5 $\pm$ 0.4	2	1.5 $\pm$ 0.5	3	0.7 $\pm$ 0.4	0
	$>$200	0.81 $\pm$ 0.21	0	1.1 $\pm$ 0.4	2	0.40 $\pm$ 0.24	0
120–160	50–100	9.6 $\pm$ 1.7	8	23 $\pm$ 5	29	2.7 $\pm$ 0.5	4
	100–150	3.3 $\pm$ 0.8	2	3.4 $\pm$ 0.7	4	0.71 $\pm$ 0.22	2
	150–200	0.26 $\pm$ 0.10	0	0.72 $\pm$ 0.19	1	0.38 $\pm$ 0.14	0
	$>$200	0.29 $\pm$ 0.11	0	0.36 $\pm$ 0.12	1	0.24 $\pm$ 0.20	0
0–120	50–100	132 $\pm$ 19	138	776 $\pm$ 125	821	45 $\pm$ 7	49
	100–150	20 $\pm$ 4	16	131 $\pm$ 30	123	10.0 $\pm$ 1.9	10
	150–200	4.0 $\pm$ 0.8	5	34 $\pm$ 8	34	2.5 $\pm$ 0.5	4
	$>$200	1.9 $\pm$ 0.4	2	21 $\pm$ 7	14	1.2 $\pm$ 0.3	4

The corresponding results for $\mathrm {e}\mathrm {e}\mu$ and $\mathrm {e}\mu \mu$ events without an OSSF pair, for events with a same-sign $\mathrm {e}\mathrm {e}$ , $\mathrm {e}\mu$, or $\mu \mu$ pair and one $\tau _\mathrm {h}$ candidate, and for events with an opposite-sign $\mathrm {e}\mu$ pair and one $\tau _\mathrm {h}$ candidate, are presented in Appendix A. The different leptonic content in these search channels provides sensitivity to various classes of SUSY models (Sect. 9).

## Search in the four-lepton final state

As mentioned in the introduction, we interpret our four-lepton final state results in the context of a GMSB model, in combination with results from a study with two leptons and at least two jets, which is presented in Sect. 6. This situation motivates the use of four-lepton channels with at least one OSSF pair that is consistent with a $\mathrm{Z}$ boson decay. The data are binned in intervals of $E_{\mathrm {T}}^{\text {miss}}$ in order to discriminate between signal and background.

We use the same object selection as for the three-lepton final state, requiring exactly four leptons (electrons, muons, and at most one $\tau _\mathrm {h}$ candidate). We require that there be an $\mathrm {e}\mathrm {e}$ or $\mu \mu$ OSSF pair with an invariant mass within $15$ GeV of the nominal $\mathrm{Z}$ boson mass. The background determination methods are also the same as described for the three-lepton final state. The main background, from $\mathrm{Z}\mathrm{Z}$ production, is thus estimated from simulation, with corrections applied to the predicted $E_{\mathrm {T}}^{\text {miss}}$ spectrum as described in Sect. 3.1.1. Backgrounds from hadrons that are misreconstructed as leptons or from non-prompt leptons are evaluated using control samples in the data as described in Sect. 3.1.2.

Table 2 summarizes the results. We consider events with exactly one OSSF pair and no $\tau _\mathrm {h}$ candidate, with exactly one OSSF pair and one $\tau _\mathrm {h}$ candidate, and with exactly two OSSF pairs and no $\tau _\mathrm {h}$ candidate. The distribution of $E_{\mathrm {T}}^{\text {miss}}$ versus $M_{\ell \ell }$ for events without a $\tau _\mathrm {h}$ candidate is presented in Fig. 26 of Appendix A.

**Table 2:** Observed yields and SM expectations for exclusive channels of four-lepton final states. All categories require four leptons including an OSSF ($\mathrm {e}\mathrm {e}$ or $\mu \mu$) pair consistent with a $\mathrm{Z}$ boson. The three sections refer, respectively, to events with one OSSF pair and no $\tau _\mathrm {h}$ candidate, one OSSF pair and one $\tau _\mathrm {h}$ candidate, and two OSSF pairs and no $\tau _\mathrm {h}$ candidate. The uncertainties include both the statistical and systematic components

$E_{\mathrm {T}}^{\text {miss}}$ ( GeVns)	Observed	Total background
1 OSSF pair, 0 $\tau _\mathrm {h}$		
0–30	1	2.3 $\pm$ 0.6
30–50	3	1.2 $\pm$ 0.3
50–100	2	1.5 $\pm$ 0.4
$>$100	2	0.8 $\pm$ 0.3
1 OSSF pair, 1 $\tau _\mathrm {h}$		
0–30	33	25 $\pm$ 12
30–50	11	11 $\pm$ 3.1
50–100	9	9.3 $\pm$ 1.9
$>$100	2	2.9 $\pm$ 0.6
2 OSSF pairs, 0 $\tau _\mathrm {h}$		
0–30	142	149 $\pm$ 46
30–50	25	28 $\pm$ 11
50–100	4	4.5 $\pm$ 2.7
$>$100	1	0.8 $\pm$ 0.3

## Search in the same-sign two-lepton final state

Three-lepton final states are not sensitive to the chargino–neutralino pair production processes of Fig. 1 if one of the leptons is unidentified, not isolated, or outside the acceptance of the analysis. For small mass differences between the SUSY particle states in Fig. 1, one of the leptons might be too soft to be included in the analysis. Some of these otherwise-rejected events can be recovered by requiring only two leptons. These leptons should have the same sign (SS) to suppress the overwhelming background from opposite-sign lepton pairs.

We therefore perform a search for events with an SS lepton pair, using the selection and methodology presented in Reference [17]. We require events to contain exactly one SS $\mathrm {e}\mathrm {e}$, $\mathrm {e}\mu$, or $\mu \mu$ pair, where the $\mathrm {e}$ and $\mu$ candidates must satisfy $p_{\mathrm {T}} >20$ GeV and $|\eta |<2.4$. To better reject background from fake leptons, we tighten the $\mathrm {e}$ ($\mu$) isolation requirement to $I_\text {rel} <0.09$ $(0.10)$ and the $d_0$ requirement to 0.1 (0.05) mm.

Background from processes such as WZ and ${\mathrm{t}}\overline{{\mathrm{t}}}$Z production is reduced by requiring $E_{\mathrm {T}}^{\text {miss}} > 120$ GeV. This background is further reduced by rejecting events that, after applying looser $\mathrm {e}$ and $\mu$ selection criteria, contain an OSSF pair within 15 GeV of the $\mathrm{Z}$ boson mass.

We evaluate the background from $\mathrm{W}\mathrm{Z}$ events using simulated events and assign a 15 % systematic uncertainty, which accounts for the difference between the observed and simulated yields in a $\mathrm{W}\mathrm{Z}$-event-enriched data control sample obtained by inverting the $\mathrm{Z}$-boson veto. A second background is from events containing a prompt lepton from a W boson decay and a genuine lepton of the same sign from heavy-flavor decay or a misidentified hadron (mainly from ${\mathrm{t}}\overline{{\mathrm{t}}}$events). We evaluate this background by determining the probability for a loosely identified electron or muon to satisfy the selection criteria in a background-enriched control region [17]. We assign a 50 % systematic uncertainty to this background based on the difference in sample composition between the control regions used to measure this probability and the signal regions. A third background is from events with two opposite-sign leptons, in which one of the leptons is an electron with an incorrect charge assignment caused by severe bremsstrahlung. To evaluate this background, we select opposite-sign events that satisfy the selection, weighted by the probability of electron-charge misassignment, determined using $\mathrm{Z}\rightarrow \mathrm {e}\mathrm {e}$ events. Finally, background from rare SM processes, such as those described in Sect. 3.1, is estimated from simulation and assigned an uncertainty of 50 %.

Two search regions are defined, one by $E_{\mathrm {T}}^{\text {miss}} > 200$ GeV, and the other by $120 < E_{\mathrm {T}}^{\text {miss}} < 200$ GeV and $N_{\text {jets}} =0$, where $N_{\text {jets}}$ for this purpose denotes the number of jets with $p_{\mathrm {T}} > 40$ GeV and $|\eta | < 2.5$. The jet veto enhances the sensitivity to the signal models targeted here by suppressing backgrounds with large hadronic activity, such as ${\mathrm{t}}\overline{{\mathrm{t}}}$events.

**Fig. 6:**
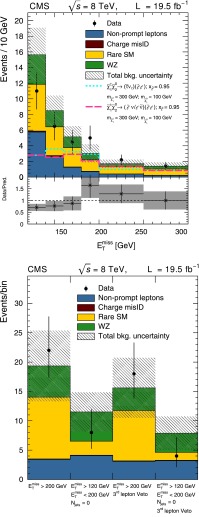
$E_{\mathrm {T}}^{\text {miss}}$ distribution for same-sign dilepton candidates in comparison with the SM expectations (*top*). The *bottom panel* shows the ratio and corresponding uncertainty of the observed and total SM expected distributions. The third lepton veto is not applied. The distributions of example signal scenarios are overlaid. Observed yields and expected backgrounds for the different search regions (*bottom*). In both plots, events with $E_{\mathrm {T}}^{\text {miss}} >120$ GeV are displayed, and the *hashed band* shows the combined statistical and systematic uncertainties of the total background

The observed yields and corresponding SM expectations are given in Table 3. Results are presented both with and without the veto of events with a third selected lepton. The distribution of $E_{\mathrm {T}}^{\text {miss}}$ in comparison with the SM expectation is shown in Fig. 6, along with the observations and expectations in each search region. The interpretation, presented in Sect. 9, is based on the two signal regions defined above, and includes the third lepton veto in order to simplify combination with the results of the three-lepton search.

**Table 3:** Observed yields and SM expectations for the same-sign dilepton search, with and without a veto on the presence of a third lepton. The uncertainties include both the statistical and systematic components. The $N_{\text {jets}}$ variable refers to the number of jets with $p_{\mathrm {T}} > 40$ GeV and $|\eta | < 2.5$.

Sample	$E_{\mathrm {T}}^{\text {miss}} >200$ GeV	$E_{\mathrm {T}}^{\text {miss}}$ 120–200 GeV, $N_{\text {jets}} = 0$	$E_{\mathrm {T}}^{\text {miss}} >200$ GeV, $3{\text {rd}}$ lepton veto	$E_{\mathrm {T}}^{\text {miss}}$ 120–200 GeV, $N_{\text {jets}} = 0$, $3{\text {rd}}$ lepton veto
Non-prompt leptons	3.4 $\pm$ 1.9	4.1 $\pm$ 2.2	3.1 $\pm$ 1.7	3.2 $\pm$ 1.7
Charge misidentification	0.09 $\pm$ 0.01	0.08 $\pm$ 0.01	0.09 $\pm$ 0.01	0.07 $\pm$ 0.01
Rare SM	10.5 $\pm$ 5.7	2.4 $\pm$ 2.4	8.6 $\pm$ 4.8	1.4 $\pm$ 2.1
WZ	5.3 $\pm$ 0.8	5.0 $\pm$ 0.8	3.9 $\pm$ 0.6	3.3 $\pm$ 0.5
Total background	19.4 $\pm$ 6.0	11.5 $\pm$ 3.3	15.6 $\pm$ 5.1	7.9 $\pm$ 2.8
Data	22	8	18	4

## Search in the $\mathrm{W}\mathrm{Z}/\mathrm{Z}\mathrm{Z}+ E_{\mathrm {T}}^{\text {miss}}$ final state with two leptons and two jets

The three- and four-lepton searches described above are sensitive not only to the processes of Fig. 1, but also to those of Fig. 2, with on-shell or off-shell vector bosons. In this section, we describe a search for events with two leptons consistent with a $\mathrm{Z}$ boson and at least two jets ($\mathrm{Z}+\mathrm{dijet}$), which extends the sensitivity to some of the processes of Fig. 2. Specifically, we select events in which an on-shell $\mathrm{Z}$ boson decays to either an $\mathrm {e}^+\mathrm {e}^-$ or $\mu ^+\mu ^-$ pair, while an on-shell $\mathrm{W}$ boson or another on-shell $\mathrm{Z}$ boson decays to two jets. The object selection and background determination procedures are based on those presented in Reference [9]: both leptons must have $p_{\mathrm {T}} >20$ GeV and the dilepton invariant mass must be consistent with the $\mathrm{Z}$ boson mass to within $10$ GeV. At least two jets with $p_{\mathrm {T}} >30$ GeV are required. Events with a third lepton are rejected in order to reduce the background from $\mathrm{W}\mathrm{Z}$ production.

Following the lepton and jet selection, the dominant background is from $\mathrm{Z}+\text {jets}$ events. This background is strongly suppressed by requiring large values of $E_{\mathrm {T}}^{\text {miss}}$, leaving ${\mathrm{t}}\overline{{\mathrm{t}}}$ production as the dominant background. The ${\mathrm{t}}\overline{{\mathrm{t}}}$ background is reduced by a factor of $\sim 10$ by applying the veto on events with $\mathrm{b}\ \text {jets}$ mentioned in Sect. 2. Background from ${\mathrm{t}}\overline{{\mathrm{t}}}$ and $\mathrm{Z}+\text {jets}$ events is reduced further by requiring the dijet mass $M_\text {jj}$ formed from the two highest $p_{\mathrm {T}}$ jets to be consistent with a $\mathrm{W}$ or $\mathrm{Z}$ boson, namely $70 < M_\text {jj} < 110$ GeV.

For the remaining background from $\mathrm{Z}+\text {jets}$ events, significant $E_{\mathrm {T}}^{\text {miss}}$ arises primarily because of the mismeasurement of jet $p_{\mathrm {T}}$. We evaluate this background using a sample of $\gamma +\text {jets}$ events as described in Reference [9], accounting for the different kinematic properties of the events in the control and signal samples.

The remaining background other than that from $\mathrm{Z}+\text {jets}$ events is dominated by ${\mathrm{t}}\overline{{\mathrm{t}}}$ production, but includes events with $\mathrm{W}\mathrm{W}$, single-top-quark, and $\tau \tau$ production. This background is characterized by equal rates of $\mathrm {e}\mathrm {e}+\mu \mu$ versus $\mathrm {e}\mu$ events and so is denoted “flavor symmetric” (FS). To evaluate the FS background, we use an $\mathrm {e}\mu$ control sample, and correct for the different electron vs. muon selection efficiencies. The SM backgrounds from events with $\mathrm{W}\mathrm{Z}$ and $\mathrm{Z}\mathrm{Z}$ production are estimated from simulation and assigned uncertainties based on comparisons with data in control samples with exactly three leptons ($\mathrm{W}\mathrm{Z}$ control sample) and exactly four leptons ($\mathrm{Z}\mathrm{Z}$ control sample), and at least two jets. Background from rare SM processes with ${{\mathrm{t}}\overline{{\mathrm{t}}}}\mathrm{Z}$, $\mathrm{Z}\mathrm{Z}\mathrm{Z}, \mathrm{Z}\mathrm{Z}\mathrm{W}$, and $Z\mathrm{W}\mathrm{W}$ production is determined from simulation with an assigned uncertainty of 50 %. The background estimation methodology is validated in a signal-depleted control region, defined by $M_\text {jj} > 110$ GeV, which is orthogonal to the search region. The observed yields are found to be consistent with the expected backgrounds in this control region.

**Fig. 7:**
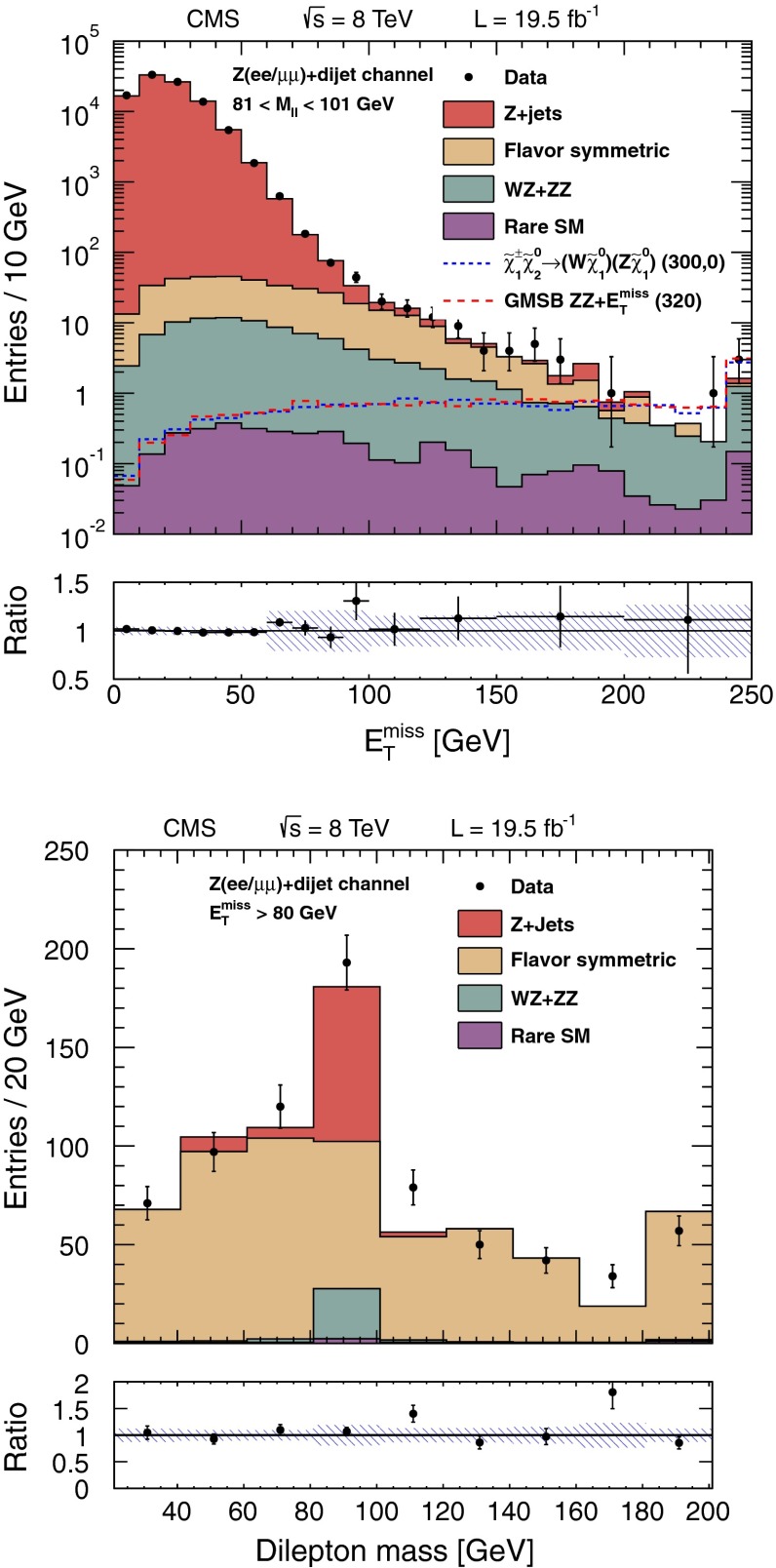
Distributions for $\mathrm{Z}+\text {dijet}$ events in comparison with SM expectations: (*top*) $E_{\mathrm {T}}^{\text {miss}}$ distribution for events with the dilepton invariant mass satisfying $81 < M_{\ell \ell } < 101$ GeV; expected results for two signal scenarios are overlaid, (*bottom*) $M_{\ell \ell }$ distribution for $E_{\mathrm {T}}^{\text {miss}} >80$ GeV. The ratio of the observed to predicted yields in each bin is shown in the *lower panels*. The *error bars* indicate the statistical uncertainties of the data and the *shaded band* the total background uncertainty

The results are presented in Table 4. The five exclusive intervals with $E_{\mathrm {T}}^{\text {miss}} >80$ GeV are treated as signal regions in the interpretations presented in Sect. 9. Figure 7 displays the observed $E_{\mathrm {T}}^{\text {miss}}$ and dilepton mass distributions compared with the sum of the expected backgrounds.

**Table 4:** Observed yields and SM expectations, in bins of $E_{\mathrm {T}}^{\text {miss}}$, for the $\mathrm{Z}+\text {dijet}$ analysis. The total background is the sum of the $\mathrm{Z}+\text {jets}$ background, the flavor-symmetric (FS) background, and the $\mathrm{W}\mathrm{Z}, \mathrm{Z}\mathrm{Z}$, and rare SM backgrounds. All uncertainties include both the statistical and systematic components. The expected yields for the $\mathrm{W}\mathrm{Z}+E_{\mathrm {T}}^{\text {miss}}$ model with $m_{\widetilde{\chi }}=300$ GeV and $m_{\widetilde{\chi }^{0} _{1}}=0$ GeV, and the GMSB $\mathrm{Z}\mathrm{Z}+E_{\mathrm {T}}^{\text {miss}}$ model with $\mu =320$ GeV (see Sect. 9.3) are also indicated

Sample	$E_{\mathrm {T}}^{\text {miss}}$ 0–30 GeV	$E_{\mathrm {T}}^{\text {miss}}$ 30–60 GeV	$E_{\mathrm {T}}^{\text {miss}}$ 60–80 GeV	$E_{\mathrm {T}}^{\text {miss}}$ 80–100 GeV
$\mathrm{Z}+\text {jets}$ bkg	75,839 $\pm$ 3,042	21,234 $\pm$ 859	690 $\pm$ 154	65 $\pm$ 22
FS bkg	70 $\pm$ 12	97 $\pm$ 16	48.3 $\pm$ 8.3	35.2 $\pm$ 6.2
WZ bkg	16.1 $\pm$ 8.1	27 $\pm$ 14	11.8 $\pm$ 5.9	6.8 $\pm$ 3.4
ZZ bkg	2.9 $\pm$ 1.4	6.0 $\pm$ 3.0	3.3 $\pm$ 1.7	2.8 $\pm$ 1.4
Rare SM bkg	0.5 $\pm$ 0.2	1.0 $\pm$ 0.5	0.6 $\pm$ 0.3	0.5 $\pm$ 0.2
Total bkg	75,929 $\pm$ 3,042	21,364 $\pm$ 859	754 $\pm$ 154	110 $\pm$ 23
Data	76,302	20,991	809	115
$\mathrm{W}\mathrm{Z}+E_{\mathrm {T}}^{\text {miss}}$ (300/0)	0.6 $\pm$ 0.1	1.4 $\pm$ 0.1	1.2 $\pm$ 0.1	1.3 $\pm$ 0.1
GMSB (320)	0.5 $\pm$ 0.0	1.5 $\pm$ 0.1	1.4 $\pm$ 0.1	1.4 $\pm$ 0.1

Sample	$E_{\mathrm {T}}^{\text {miss}}$ 100–120 GeV	$E_{\mathrm {T}}^{\text {miss}}$ 120–150 GeV	$E_{\mathrm {T}}^{\text {miss}}$ 150–200 GeV	$E_{\mathrm {T}}^{\text {miss}} > 200$ GeV
$\mathrm{Z}+\text {jets}$ bkg	7.8 $\pm$ 3.1	3.7 $\pm$ 1.6	2.0 $\pm$ 1.0	0.4 $\pm$ 0.3
FS bkg	21.9 $\pm$ 4.0	13.2 $\pm$ 2.5	5.7 $\pm$ 1.6	0.8 $\pm$ 0.4
WZ bkg	3.7 $\pm$ 1.9	2.9 $\pm$ 1.5	1.9 $\pm$ 0.9	0.9 $\pm$ 0.4
ZZ bkg	1.8 $\pm$ 0.9	1.9 $\pm$ 0.9	1.4 $\pm$ 0.7	1.3 $\pm$ 0.7
Rare SM bkg	0.2 $\pm$ 0.1	0.4 $\pm$ 0.2	0.4 $\pm$ 0.2	0.3 $\pm$ 0.1
Total bkg	35.4 $\pm$ 5.5	22.2 $\pm$ 3.5	11.3 $\pm$ 2.2	3.6 $\pm$ 1.0
Data	36	25	13	4
$\mathrm{W}\mathrm{Z}+E_{\mathrm {T}}^{\text {miss}}$ (300/0)	1.5 $\pm$ 0.1	2.3 $\pm$ 0.1	3.4 $\pm$ 0.1	5.2 $\pm$ 0.2
GMSB (320)	1.4 $\pm$ 0.1	2.2 $\pm$ 0.1	3.9 $\pm$ 0.1	5.7 $\pm$ 0.2

## Searches in the $\mathrm{W}\mathrm{H}+ E_{\mathrm {T}}^{\text {miss}}$ final state

The recent observation of a Higgs boson [54–56] offers the novel possibility to perform beyond-the-SM searches by exploiting the measured properties of this particle. In particular, the heavy neutralinos are expected to decay predominantly via a Higgs boson in large regions of SUSY parameter space, and in this section we report searches for such decays.

Three exclusive final states sensitive to the process of Fig. 2 (center) are considered here. In all searches, the $\mathrm{W}$ boson is required to decay leptonically. A search in the single-lepton final state provides sensitivity to events in which the Higgs boson decays to a $\mathrm{b}\overline{\text {b}}$ pair. A search in the same-sign dilepton final state targets events with the decay $\mathrm{H}\rightarrow \mathrm{W}^+\mathrm{W}^-$ in which one of the $\mathrm{W}$ bosons decays leptonically and the other hadronically. The results of the CMS inclusive multilepton search [57] are reinterpreted, covering final states with at least three leptons. It is used to target the decays $\mathrm{H}\rightarrow \mathrm {W^+}\mathrm {W^-}$, $\mathrm{H}\rightarrow \mathrm{Z}\mathrm{Z}$, and $\mathrm{H}\rightarrow \tau ^+\tau ^-$, where the $\mathrm{W}$ and $\mathrm{Z}$ bosons, and the $\tau$ lepton, decay leptonically. The results from these searches are combined to place limits on the production of the $\mathrm{W}\mathrm{H}+E_{\mathrm {T}}^{\text {miss}}$ final state.

### Search in the single-lepton final state

#### Overview of the search

In this section we report the results from a search for $\widetilde{\chi }^{\pm } _{1}\widetilde{\chi }^{0} _{2}\rightarrow (\mathrm{W}\widetilde{\chi }^{0} _{1})(\mathrm{H}\widetilde{\chi }^{0} _{1}) \rightarrow \ell \nu \mathrm{b}\overline{\text {b}} +E_{\mathrm {T}}^{\text {miss}}$ events. Previous searches involving the $\mathrm{H}\rightarrow \mathrm{b}\overline{\text {b}}$ decay mode, corresponding to the largest SM branching fraction (56 %) [58], have targeted the associated production with a leptonically decaying $\mathrm{W}$ boson [59]. In the present search, we impose additional kinematic requirements on $E_{\mathrm {T}}^{\text {miss}}$ and related quantities. These requirements strongly suppress both the SM backgrounds and the SM production of a Higgs boson while retaining efficiency for the SUSY signal. This search is an extension of a search for direct top-squark pair production [14], which targets events with a single lepton, at least four jets, and $E_{\mathrm {T}}^{\text {miss}}$, with similar object selection and analysis methodology. The final state considered here is similar, except that we expect only two jets.

Events are required to contain a single lepton, exactly two b jets, and $E_{\mathrm {T}}^{\text {miss}}$. The largest background arises from ${\mathrm{t}}\overline{{\mathrm{t}}}$ production, due both to semileptonic ${\mathrm{t}}\overline{{\mathrm{t}}}$ events and to events where both top quarks decay leptonically but one lepton is not identified. Events with $\mathrm{W}+\text {jets}$ production also constitute an important source of background. The SM backgrounds are suppressed using several kinematic requirements based on large values of $E_{\mathrm {T}}^{\text {miss}}$. Signal regions are defined by successively tighter requirements on $E_{\mathrm {T}}^{\text {miss}}$. The signal is expected to produce a peak in the dijet mass spectrum at $M_{\mathrm{b}\overline{\text {b}}}=m_{\mathrm{H}}$.

#### Event selection

Events are required to contain exactly one electron (muon) with $p_{\mathrm {T}} >30$ (25) GeV and $|\eta |<1.4442\,(2.1)$. Electrons are restricted to the central region of the detector for consistency with the search for top-squarks [14]. There must be exactly two jets with $|\eta |<2.4$ and no jets with $2.4 < |\eta | < 4.7$. This latter requirement substantially reduces the ${\mathrm{t}}\overline{{\mathrm{t}}}\rightarrow \ell +\mathrm {jets}$ background, which typically has four jets. The two selected jets must satisfy the CSVM b-tagging criteria and have $p_{\mathrm {T}} >30$ GeV. We require $M_{\mathrm {T}} > 100$ GeV, which primarily rejects backgrounds with a single $\mathrm{W}\rightarrow \ell \nu$ decay and no additional $E_{\mathrm {T}}^{\text {miss}}$, such as ${\mathrm{t}}\overline{{\mathrm{t}}}\rightarrow \ell +\mathrm {jets}$, $\mathrm{W}+\text {jets}$, and SM $\mathrm{W}\mathrm{H}\rightarrow \ell \nu \mathrm{b}\overline{\text {b}}$ events, and single-top-quark events in the $t$ and $s$ channels. To suppress the dilepton ${\mathrm{t}}\overline{{\mathrm{t}}}$ backgrounds, events with an isolated high-$p_{\mathrm {T}}$ track or $\tau _\mathrm {h}$ candidate are rejected.

Further suppression of the ${\mathrm{t}}\overline{{\mathrm{t}}}$ backgrounds is achieved by using the $M_{\mathrm {T2}}^{\mathrm {bl}}$ variable [60], which is defined as the minimum “mother” particle mass compatible with the four-momentum of the lepton, b-tagged jets, and $E_{\mathrm {T}}^{\text {miss}}$. It has an endpoint at the top-quark mass for ${\mathrm{t}}\overline{{\mathrm{t}}}$ events without mismeasurement effects, while signal events may have larger values. We require $M_{\mathrm {T2}}^{\mathrm {bl}}> 200$ GeV.

The dijet mass $M_{\mathrm{b}\overline{\text {b}}}$ formed from the two selected jets is required to satisfy $100< M_{\mathrm{b}\overline{\text {b}}} <150$ GeV. This requirement has an efficiency of about 80 % for signal events.

#### Backgrounds and their estimation methodology

Backgrounds are grouped into six categories. The largest background arises from ${\mathrm{t}}\overline{{\mathrm{t}}}$ events and from single-top-quark production in the tW channel, in which both $\mathrm{W}$ bosons decay leptonically (dilepton top-quark background). Backgrounds from ${\mathrm{t}}\overline{{\mathrm{t}}}$ and single-top-quark production with one leptonically decaying $\mathrm{W}$ boson are referred to as the single-lepton top-quark background. Backgrounds from $\mathrm{W}$ $\mathrm{Z}$ production, where the $\mathrm{W}$ boson decays leptonically and the $\mathrm{Z}$ boson decays to a $\mathrm{b}\overline{\text {b}}$ pair, are referred to as the $\mathrm{W}\mathrm{Z}\rightarrow \ell \nu \mathrm{b}\overline{\text {b}}$ background. Backgrounds from $\mathrm{W}$ bosons produced in associated production with a $\mathrm{b}\overline{\text {b}}$ pair are referred to as the $\mathrm{W}+\mathrm{b}\overline{\text {b}}$ background, while production of $\mathrm{W}$ bosons with other partons constitutes the $\mathrm{W}+\text {light-flavor jets}$ background. Finally, the “rare background” category consists of processes with two top quarks and a W, Z or Higgs boson, as well as diboson, triboson, $\mathrm{Z}+\text {jets}$, and SM $\mathrm{W}\mathrm{H}\rightarrow \ell \nu \mathrm{b}\overline{\text {b}}$ events. The $\mathrm{Z}+\text {jets}$ process has a large cross section but is included in the rare background category because its contribution is very small after the signal-region requirements are imposed. With the exception of the $\mathrm{W}+\text {light-flavor jets}$ background, the background estimation is based on simulation.

The simulation is validated in three data control regions (CR) that are enriched in different background components. A data sample enriched in $\mathrm{W}+\text {light-flavor jets}$ is obtained by vetoing events with b-tagged jets (CR-0b). A data sample enriched in the dilepton top-quark background is obtained by requiring either exactly two leptons satisfying the lepton selection criteria, or one such lepton and an isolated high-$p_{\mathrm {T}}$ track (CR-2$\ell$). Finally, the $M_{\mathrm{b}\overline{\text {b}}}$ requirement is inverted to obtain a data sample (CR-$M_{\mathrm{b}\overline{\text {b}}}$) consisting of a mixture of backgrounds with similar composition as the signal region.

The agreement between the data and the simulation in the three data control regions is used to determine scale factors and uncertainties for the background predictions. In CR-2$\ell$, the data are found to agree with the predictions from simulation, which are dominated by the dilepton top-quark background. A 40 % uncertainty is assessed on the dilepton top-quark background, based on the limited statistical precision of the event sample after applying all the kinematical requirements. Correction factors of $0.8\pm 0.3$, $1.2\pm 0.5$, and $1.0\pm 0.6$ are evaluated for the $\mathrm{W}\mathrm{Z}\rightarrow \ell \nu \mathrm{b}\overline{\text {b}}$, $\mathrm{W}+\mathrm{b}\overline{\text {b}}$, and single-lepton top-quark backgrounds, respectively, based on studies of the CR-$M_{\mathrm{b}\overline{\text {b}}}$ and CR-0b samples. The rare backgrounds are taken from simulation with a 50 % systematic uncertainty.

The $\mathrm{W}+\text {light-flavor jets}$ background prediction is evaluated using the CR-0b sample, using the b-tagging misidentification rate for light flavor jets predicted by simulation. This rate includes all flavors except b quarks. The uncertainty is 40 %, due to uncertainties in the b-tagging misidentification rate and its variation with jet $p_{\mathrm {T}}$.

#### Results

**Fig. 8:**
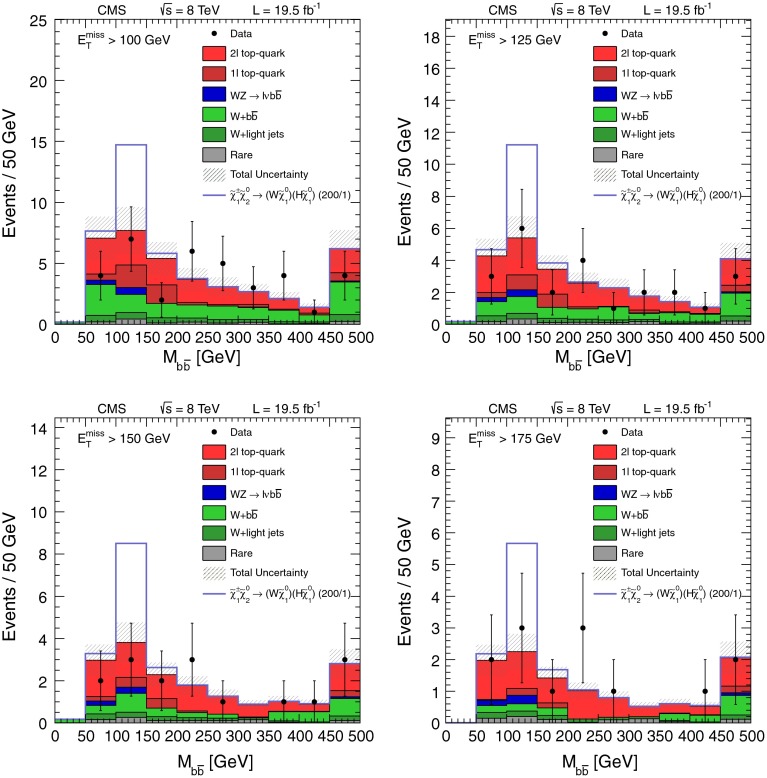
Distributions of $M_{\mathrm{b}\overline{\text {b}}}$ for the single-lepton $\mathrm{W}\mathrm{H}+E_{\mathrm {T}}^{\text {miss}}$ analysis for (*upper left*) $E_{\mathrm {T}}^{\text {miss}} >100$ GeV, (*upper right*) $E_{\mathrm {T}}^{\text {miss}} >125$ GeV, (*lower left*) $E_{\mathrm {T}}^{\text {miss}} >150$ GeV, and (*lower right*) $E_{\mathrm {T}}^{\text {miss}} >175$ GeV after all signal region requirements have been applied except for that on $M_{\mathrm{b}\overline{\text {b}}}$. The data are compared to the sum of the expected backgrounds. The labels “2$\ell$ top” and “1$\ell$ top” refer to the dilepton top-quark and single-lepton top-quark backgrounds, respectively. The *band indicates* the total uncertainty of the background prediction. Results from an example signal scenario are shown, stacked on top of the SM background

Four overlapping signal regions are defined by the requirements $E_{\mathrm {T}}^{\text {miss}} >100,125,150$, and 175 GeV. In general, signal regions with tighter $E_{\mathrm {T}}^{\text {miss}}$ requirements are more sensitive to signal scenarios with larger mass differences $m_{\widetilde{\chi }}- m_{\widetilde{\chi }^{0} _{1}}$. The results for these signal regions are summarized in Table 5. The data are seen to agree with the background predictions to within the uncertainties. The expected yields for several signal scenarios are indicated, including systematic uncertainties that are discussed in Sect.  9. The distributions of $M_{\mathrm{b}\overline{\text {b}}}$ are displayed in Fig. 8. No evidence for a peak at $M_{\mathrm{b}\overline{\text {b}}}=m_{\mathrm{H}}$ is observed.

**Table 5:** Observed yields and SM expectations, in several bins of $E_{\mathrm {T}}^{\text {miss}}$, for the single-lepton $\mathrm{W}\mathrm{H}+E_{\mathrm {T}}^{\text {miss}}$ analysis. The expectations from several signal scenarios are shown; the first number indicates $m_{\widetilde{\chi }}$ and the second $m_{\widetilde{\chi }^{0} _{1}}$( GeV). The uncertainties include both the statistical and systematic components

Sample	$E_{\mathrm {T}}^{\text {miss}} > 100$ GeV	$E_{\mathrm {T}}^{\text {miss}} > 125$ GeV	$E_{\mathrm {T}}^{\text {miss}} > 150$ GeV	$E_{\mathrm {T}}^{\text {miss}} > 175$ GeV
Dilepton top-quark	2.8 $\pm$ 1.2	2.3 $\pm$ 1.0	1.7 $\pm$ 0.7	1.2 $\pm$ 0.5
Single-lepton top-quark	1.8 $\pm$ 1.1	0.9 $\pm$ 0.6	0.5 $\pm$ 0.3	0.2 $\pm$ 0.2
$\mathrm{W}\mathrm{Z}\rightarrow \ell \nu \mathrm{b}\overline{\text {b}}$	0.6 $\pm$ 0.2	0.4 $\pm$ 0.2	0.3 $\pm$ 0.1	0.3 $\pm$ 0.1
$\mathrm{W}+\mathrm{b}\overline{\text {b}}$	1.5 $\pm$ 0.9	1.0 $\pm$ 0.7	0.9 $\pm$ 0.6	0.2 $\pm$ 0.3
$\mathrm{W}+\text {light-flavor jets}$	0.5 $\pm$ 0.2	0.3 $\pm$ 0.1	0.2 $\pm$ 0.1	0.2 $\pm$ 0.1
Rare	0.4 $\pm$ 0.2	0.3 $\pm$ 0.2	0.3 $\pm$ 0.2	0.2 $\pm$ 0.1
Total background	7.7 $\pm$ 1.9	5.4 $\pm$ 1.3	3.8 $\pm$ 1.0	2.3 $\pm$ 0.6
Data	7	6	3	3
$\widetilde{\chi }^{\pm } _{1}\widetilde{\chi }^{0} _{2}\rightarrow (\mathrm{W}\widetilde{\chi }^{0} _{1})(\mathrm{H}\widetilde{\chi }^{0} _{1})$ (130/1)	9.0 $\pm$ 1.2	7.5 $\pm$ 1.0	6.0 $\pm$ 0.8	4.5 $\pm$ 0.6
$\widetilde{\chi }^{\pm } _{1}\widetilde{\chi }^{0} _{2}\rightarrow (\mathrm{W}\widetilde{\chi }^{0} _{1})(\mathrm{H}\widetilde{\chi }^{0} _{1})$ (150/1)	7.2 $\pm$ 1.0	6.1 $\pm$ 0.9	5.0 $\pm$ 0.7	3.5 $\pm$ 0.5
$\widetilde{\chi }^{\pm } _{1}\widetilde{\chi }^{0} _{2}\rightarrow (\mathrm{W}\widetilde{\chi }^{0} _{1})(\mathrm{H}\widetilde{\chi }^{0} _{1})$ (200/1)	7.0 $\pm$ 0.9	5.8 $\pm$ 0.8	4.7 $\pm$ 0.7	3.4 $\pm$ 0.5
$\widetilde{\chi }^{\pm } _{1}\widetilde{\chi }^{0} _{2}\rightarrow (\mathrm{W}\widetilde{\chi }^{0} _{1})(\mathrm{H}\widetilde{\chi }^{0} _{1})$ (300/1)	5.2 $\pm$ 0.7	4.9 $\pm$ 0.7	4.4 $\pm$ 0.6	3.9 $\pm$ 0.5
$\widetilde{\chi }^{\pm } _{1}\widetilde{\chi }^{0} _{2}\rightarrow (\mathrm{W}\widetilde{\chi }^{0} _{1})(\mathrm{H}\widetilde{\chi }^{0} _{1})$ (400/1)	3.2 $\pm$ 0.4	3.0 $\pm$ 0.4	2.8 $\pm$ 0.4	2.5 $\pm$ 0.3

### Search in the same-sign dilepton final state

**Fig. 9:**
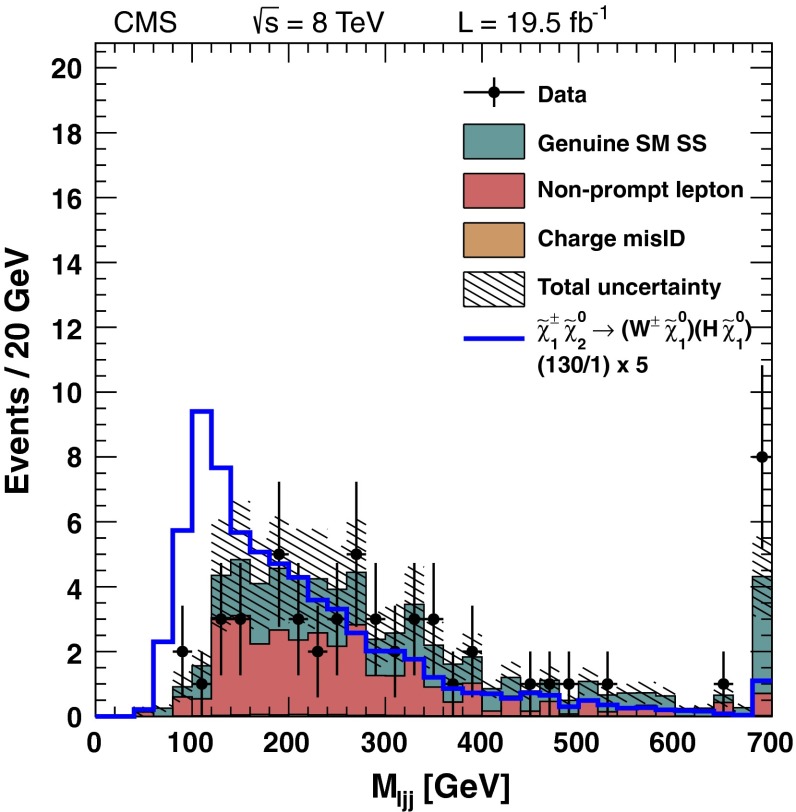
$M_{\ell \text {jj} }$ distribution for the same-sign dilepton $\mathrm{W}\mathrm{H}+E_{\mathrm {T}}^{\text {miss}}$ analysis, compared to the expected backgrounds, after all selection requirements have been applied except for that on $M_{\ell \text {jj} }$. An example signal scenario with $m_{\widetilde{\chi }}= 130$ GeV and $m_{\widetilde{\chi }^{0} _{1}}= 1$ GeV is overlaid. For better visibility, the signal normalization has been increased by a factor of five relative to the theory prediction

The object selection and background estimation methodology for the SS dilepton search follow those presented in Sect. 5. We define the quantity $M_{\ell \text {jj} }$ as the three-body invariant mass of the system obtained by combining the two highest $p_{\mathrm {T}}$ jets in an event with the lepton closest to the dijet axis. Signal events peak below $m_{\mathrm{H}}$, due to the undetected neutrino, as shown in Fig. 9. Background events generally have larger values of $M_{\ell \text {jj} }$. Events are required to satisfy $M_{\ell \text {jj} }< 120$ GeV.

We require the presence of exactly two SS leptons ($\mathrm {e}$ $\mathrm {e}$, $\mu \mu$, or $\mathrm {e}$ $\mu$), each with $p_{\mathrm {T}} > 20$ GeV, and of either exactly two or exactly three jets, each with $p_{\mathrm {T}} > 30$ GeV. The $E_{\mathrm {T}}^{\text {miss}}$ value must exceed 40 GeV. To suppress ${\mathrm{t}}\overline{{\mathrm{t}}}$background, events with a “tight” CSV b jet or with two or more “loose” CSV b jets are rejected, where the tight (loose) CSV working point corresponds to an efficiency of about 55 % (83 %) for b jets, and a misidentification probability for light-parton jets of about 0.1 % (10 %) [49]. Events with an additional electron or muon or with a $\tau _\mathrm {h}$ candidate are rejected in order to suppress background from SM processes with multiple electroweak bosons.

The transverse mass $M_{\mathrm {T}}$ is computed for each of the selected leptons, and at least one lepton must satisfy $M_{\mathrm {T}} > 110$ GeV. This requirement suppresses processes containing a single leptonically decaying W boson. We additionally require a separation $\Delta \eta (\ell _1,\ell _2)<1.6$ in order to reduce background with non-prompt leptons as well as SM events with two W bosons.

To suppress ${\mathrm{t}}\overline{{\mathrm{t}}}$ events in which the decays of a W boson and a b quark lead to an SS lepton pair, we calculate the quantity $M_{\mathrm {T2}}^{\mathrm {J}}$ [61], which is the minimum mass of a mother particle compatible with the four-momenta of the two leptons, jets, and $E_{\mathrm {T}}^{\text {miss}}$. For events with three jets, $M_{\mathrm {T2}}^{\mathrm {J}}$ is calculated with the two jets that minimize the result. We require $M_{\mathrm {T2}}^{\mathrm {J}}> 100$ GeV.

The background estimation methodology (Sect. 5) is validated using a signal-depleted data control region defined by inverting the $M_{\ell \text {jj} }$ requirement. We observe 51 events in this control region, consistent with the background estimate of $62 \pm 22$ events.

The results are summarized in Table 6. No evidence for a peak in the $M_{\ell \text {jj} }$ distribution is observed, as seen from Fig. 9. In the signal region $M_{\ell \text {jj} }< 120$ GeV, we observe three events whereas $2.9 \pm 1.2$ SM background events are expected.

**Table 6:** Observed yields and SM expectations for the same-sign dilepton $\mathrm{W}\mathrm{H}+E_{\mathrm {T}}^{\text {miss}}$ analysis. The expectations from several signal scenarios are shown; the first number indicates $m_{\widetilde{\chi }}$ and the second $m_{\widetilde{\chi }^{0} _{1}}$( GeV). The uncertainties include both the statistical and systematic components

Sample	$\mathrm {e}\mathrm {e}$	$\mu \mu$	$\mathrm {e}\mu$	Total
Non-prompt leptons	0.3 $\pm$ 0.3	0.2 $\pm$ 0.2	0.8 $\pm$ 0.5	1.3 $\pm$ 0.8
Charge misidentification	$<$0.01	$<$0.01	$<$0.03	$<$0.03
Genuine SM SS dileptons	0.4 $\pm$ 0.4	0.4 $\pm$ 0.4	0.8 $\pm$ 0.6	1.6 $\pm$ 0.9
Total background	0.7 $\pm$ 0.5	0.6 $\pm$ 0.5	1.6 $\pm$ 0.7	2.9 $\pm$ 1.2
Data	1	1	1	3
$\widetilde{\chi }^{\pm } _{1}\widetilde{\chi }^{0} _{2}\rightarrow (\mathrm{W}\widetilde{\chi }^{0} _{1})(\mathrm{H}\widetilde{\chi }^{0} _{1})$ (130/1)	0.7$\pm$0.1	0.9$\pm$0.1	1.8$\pm$0.2	3.4$\pm$0.5
$\widetilde{\chi }^{\pm } _{1}\widetilde{\chi }^{0} _{2}\rightarrow (\mathrm{W}\widetilde{\chi }^{0} _{1})(\mathrm{H}\widetilde{\chi }^{0} _{1})$ (150/1)	0.5$\pm$0.1	0.6$\pm$0.1	1.2$\pm$0.2	2.3$\pm$0.3
$\widetilde{\chi }^{\pm } _{1}\widetilde{\chi }^{0} _{2}\rightarrow (\mathrm{W}\widetilde{\chi }^{0} _{1})(\mathrm{H}\widetilde{\chi }^{0} _{1})$ (200/1)	0.19$\pm$0.03	0.35$\pm$0.05	0.52$\pm$0.07	1.1$\pm$0.1
$\widetilde{\chi }^{\pm } _{1}\widetilde{\chi }^{0} _{2}\rightarrow (\mathrm{W}\widetilde{\chi }^{0} _{1})(\mathrm{H}\widetilde{\chi }^{0} _{1})$ (300/1)	0.06$\pm$0.01	0.10$\pm$0.02	0.17$\pm$0.03	0.33$\pm$0.05
$\widetilde{\chi }^{\pm } _{1}\widetilde{\chi }^{0} _{2}\rightarrow (\mathrm{W}\widetilde{\chi }^{0} _{1})(\mathrm{H}\widetilde{\chi }^{0} _{1})$ (400/1)	0.02$\pm$0.00	0.03$\pm$0.00	0.05$\pm$0.01	0.10$\pm$0.01

### Search in the multilepton final state

For the multilepton search presented in Reference [57], events with at least three leptons are selected, including up to one $\tau _\mathrm {h}$ candidate. These events are categorized into multiple exclusive signal regions based on the number and flavor of the leptons, the presence or absence of an OSSF pair, the invariant mass of the OSSF pair (if present), the presence or absence of a tagged b jet, and the $E_{\mathrm {T}}^{\text {miss}}$ and $H_{\mathrm {T}}$ values. The most sensitive signal regions for this search are those with exactly three leptons, no tagged b jets (using the CSVM criteria), and a low $H_{\mathrm {T}}$ value.

Backgrounds from dilepton ${\mathrm{t}}\overline{{\mathrm{t}}}$ events with non-prompt leptons are evaluated from simulation, while other backgrounds with non-prompt leptons are determined using data control samples. Backgrounds from $\mathrm{W}$ $\mathrm{Z}$and $\mathrm{Z}\mathrm{Z}$ diboson processes are estimated from simulation, with a correction to the $E_{\mathrm {T}}^{\text {miss}}$ resolution based on comparisons to data in control regions.

The data yields in the signal regions are found to be consistent with the expected SM backgrounds. The observed data yields, expected SM backgrounds, and expected signal yields for the five most sensitive signal regions for the $m_{\widetilde{\chi }}=130$ GeV, $m_{\widetilde{\chi }^{0} _{1}}=1$ GeV scenario, where the multilepton analysis has the best sensitivity, are shown in Table 7. Additional signal-depleted regions are used to constrain the backgrounds and associated uncertainties. Similar tables for other scenarios are presented in Appendix B.

**Table 7:** Observed yields and SM expectations for the multilepton $\mathrm{W}\mathrm{H}+E_{\mathrm {T}}^{\text {miss}}$ search for the five signal regions with best sensitivity for the $m_{\widetilde{\chi }}=130$ GeV, $m_{\widetilde{\chi }^{0} _{1}}=1$ GeV scenario. All five signal regions require exactly three leptons, no $\tau _\mathrm {h}$ candidate, no tagged b jet, and $H_{\mathrm {T}} < 200$ GeV. The “Below Z” entries indicate the requirement of an OSSF lepton pair with $M_{\ell \ell }< 75$ GeV

OSSF pair	$E_{\mathrm {T}}^{\text {miss}}$ [$\text {GeV}$ ]	Data	Total SM	Signal
Below $\mathrm{Z}$	50–100	142	125 $\pm$ 28	24.4 $\pm$ 4.4
Below $\mathrm{Z}$	100–150	16	21.3 $\pm$ 8.0	6.8 $\pm$ 1.2
None	0–50	53	52 $\pm$ 12	8.7 $\pm$ 1.7
None	50–100	35	38 $\pm$ 15	10.8 $\pm$ 2.0
None	100–150	7	9.3 $\pm$ 4.3	3.37 $\pm$ 0.54

## Searches in the final state with a non-resonant opposite-sign dilepton pair

Finally, we present a search for events with an oppositely charged $\mathrm {e}$ $\mathrm {e}$, $\mathrm {e}$ $\mu$, or $\mu \mu$ pair in which the lepton pair is inconsistent with $\mathrm{Z}$ boson decay. The search is sensitive to the processes shown in Fig. 3.

Both leptons are required to have $p_{\mathrm {T}} >20$ GeV. The $\mathrm {e}\mathrm {e}$ or $\mu \mu$ invariant mass must differ from the $\mathrm{Z}$ boson mass by at least 15 GeV. Events must have $E_{\mathrm {T}}^{\text {miss}} >60$ GeV and no tagged b jet defined with the CSVM criteria. The remaining background is mostly composed of events with ${\mathrm{t}}\overline{{\mathrm{t}}}$ and $\mathrm{W}\mathrm{W}$ production and is reduced using the $M_{\mathrm {CT}\perp }$ variable, which is defined in Reference [62].

The $M_{\mathrm {CT}\perp }$ variable is designed to identify events with two boosted massive particles that each decay into a visible particle and an invisible one. For events with two W bosons that each decay leptonically, and for perfect event reconstruction, $M_{\mathrm {CT}\perp }$ has an endpoint at the W boson mass. In practice, because of imperfect event reconstruction, background events can appear at larger values of $M_{\mathrm {CT}\perp }$. However, for SM events, the distribution of $M_{\mathrm {CT}\perp }$ falls rapidly for $M_{\mathrm {CT}\perp }>m_\mathrm{W}$. In contrast, for the signal scenario, the $M_{\mathrm {CT}\perp }$ distribution can extend to much higher values.

The background evaluation for this search is based on templates that describe the shape of the $M_{\mathrm {CT}\perp }$ distribution for each of the major background categories. The templates are obtained either from data control samples or simulation. The template shapes are fit to data to determine their respective normalizations. Because backgrounds from Z and ZZ processes contribute predominantly to the ee and $\mu \mu$ final states, separate templates are derived for same-flavor and opposite-flavor events.

A top-quark control sample is selected by inverting the b-jet veto. The corresponding template accounts for backgrounds with ${\mathrm{t}}\overline{{\mathrm{t}}}$ events (with or without accompanying vector bosons) and single-top-quark events produced with W bosons. We verify with simulation that the corresponding $M_{\mathrm {CT}\perp }$ template accurately models the shape of the targeted event sample in the signal region.

A template derived from simulation accounts for events with diboson production and for rare events, where by ’rare’ we in this case mean events from Higgs and triboson production. The simulation is validated using control regions. A first control region is selected by requiring the dilepton mass to be consistent with the Z boson mass. A second control region is selected by requiring a third isolated electron or muon. The two control regions are dominated by events with ZZ and WZ production, respectively. The $M_{\mathrm {CT}\perp }$ distribution is found to be well described by the simulation for both control regions.

The simulation of events with WW production is validated using the three-lepton WZ-dominated control sample. One lepton is removed from the event, and its four-momentum is added to the $E_{\mathrm {T}}^{\text {miss}}$ vector. Rescaling the $M_{\mathrm {CT}\perp }$ value of each event by $m_\mathrm{W}/m_\mathrm{Z}$ yields a distribution with very similar properties to events with WW production, as verified with simulation. The number of events in the control sample is small, and we assign a systematic uncertainty to each $M_{\mathrm {CT}\perp }$ bin defined by the difference between the yield in the data control sample and the WW event simulation, or else the statistical uncertainty of the data control sample, whichever is larger.

Similarly, a template distribution for backgrounds with two leptons from an off-shell Z boson, with $E_{\mathrm {T}}^{\text {miss}}$ from misreconstructed jets, is obtained from simulation. We weight the simulated events such that the $E_{\mathrm {T}}^{\text {miss}}$ distribution agrees with data in the on-Z ($|M_{\ell \ell } - M_\mathrm{Z} | < 15$ GeV) control region. We then examine the $M_{\mathrm {CT}\perp }$ distribution in the $M_{\mathrm {CT}\perp }< 100$ GeV, on-Z control region, where this background is expected to dominate, to validate the simulation after all corrections have been applied. We assign a bin-by-bin systematic uncertainty given by the fractional difference between the data and template in this control region (around 25 % for each bin).

We construct a template describing backgrounds with a leptonically decaying W boson and a non-prompt lepton from a data control sample, obtained by selecting events with two same-charge leptons, one of which has a relative isolation in a sideband defined by $0.2<I_\text {rel} <0.3$. All other selection requirements are the same as for the nominal analysis. Due to the small number of events in the control sample, we assign a 30 % systematic uncertainty to each bin.

**Fig. 10:**
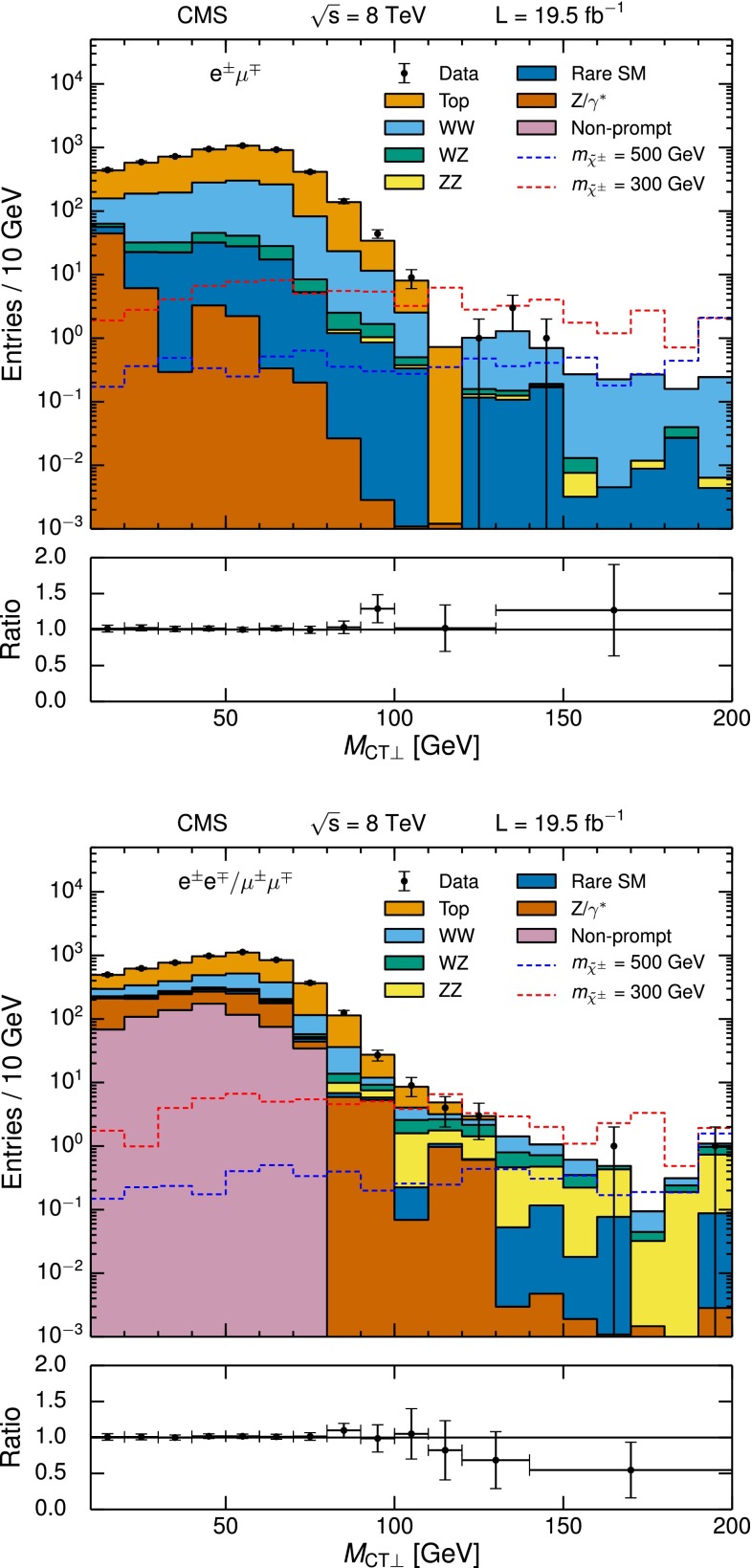
$M_{\mathrm {CT}\perp }$ distribution for the non-resonant opposite-sign dilepton analysis compared to the background prediction for the (*top*) opposite-flavor and (*bottom*) same-flavor channels. The background prediction is based on a fit of templates derived from control samples or simulation. The signal distributions with two different chargino mass values for the SUSY scenario shown in Fig. 1 (*top*) are also shown, with the LSP mass set to zero. The ratio of the data to the fitted distribution is shown in the lower panels

A binned maximum likelihood fit of the $M_{\mathrm {CT}\perp }$ distribution is performed for $M_{\mathrm {CT}\perp }>10$ GeV in order to determine the normalizations of the templates. The fit assumes the SM-only hypothesis. The fitting procedure is validated using simulation to verify that it behaves as expected both with and without injected signal. The results of the fit are presented in Table 8 and Fig. 10. We use a binned Anderson–Darling test [63] to verify that the fit results are consistent with the SM, finding a $p$ value of 0.41 with respect to SM-only pseudo-experiments.

**Table 8:** Results from a maximum likelihood fit of the background-only hypothesis to the $M_{\mathrm {CT}\perp }$ distribution in data for $M_{\mathrm {CT}\perp }> 10$ GeV for the non-resonant opposite-sign dilepton analysis. The corresponding results from simulation are also shown

Sample	Opposite flavor		Same flavor	
	Fit	Simulation	Fit	Simulation
Top quark	$3,750\pm 750$	3,360	$2,780\pm 420$	2,472
Diboson and rare SM	$1,460\pm 210$	1,433	$1,170\pm 180$	1,211
$\mathrm{Z}$/$\mathrm {\gamma }^*$	$57\pm 50$	106	$710\pm 420$	917
Non-prompt	$<$96	477	$710\pm 520$	156

We can recast the analysis as a comparison of event counts in a high-$M_{\mathrm {CT}\perp }$ signal region. To do this, we use the same templates, but fit the background normalizations in the $10 < M_{\mathrm {CT}\perp }< 120$ GeV region, where signal contributions are expected to be negligible. We then use these fitted normalizations to extrapolate to the $M_{\mathrm {CT}\perp }> 120$ GeV region. Since the ${\mathrm{t}}\overline{{\mathrm{t}}}$ and diboson background shapes are similar in the low-$M_{\mathrm {CT}\perp }$ region, we constrain the ratio of the ${\mathrm{t}}\overline{{\mathrm{t}}}$ to diboson yields to the value obtained from simulation, assigning a 10 % uncertainty.

The results are given in Table 9. The sum of the yields from the low- and extrapolated high-$M_{\mathrm {CT}\perp }$ regions agree with the yields in Table 8 to within the uncertainties. Note that the extra constraint on the ratio of the ${\mathrm{t}}\overline{{\mathrm{t}}}$ to diboson yields leads to smaller uncertainties than those in Table 8. The numbers of observed events in the high-$M_{\mathrm {CT}\perp }$ regions are found to be consistent with the background estimates, for both the opposite- and same-flavor channels.

**Table 9:** Results from a maximum likelihood fit of the background-only hypothesis to the $M_{\mathrm {CT}\perp }$ distribution in data, performed for events with $10<M_{\mathrm {CT}\perp }<120$ GeV and extrapolated to the $M_{\mathrm {CT}\perp }>120$ GeV region, for the non-resonant opposite-sign dilepton analysis. Where the predicted value is zero, the one standard deviation upper limit is given

Sample	Opposite flavor		Same flavor	
	$M_{\mathrm {CT}\perp }$ 10–120 GeV	$M_{\mathrm {CT}\perp }> 120$ GeV	$M_{\mathrm {CT}\perp }$ 10–120 GeV	$M_{\mathrm {CT}\perp }> 120$ GeV
Top quark	$3,770\pm 90$	$< 0.4$	$2,770\pm 110$	$0.35\pm 0.10$
Diboson and rare SM	$1,430\pm 110$	$4\pm 3$	$1,240\pm 90$	$9\pm 3$
Z/$\mathrm {\gamma }^*$	$57\pm 25$	$< 0.01$	$700\pm 240$	$0.6\pm 0.3$
Non-prompt	$<81$	$< 0.01$	$659\pm 77$	$< 0.5$
Total	$5,260\pm 130$	$4\pm 3$	$5,370\pm 100$	$10\pm 3$
Data	5,309	5	5,388	5

For slepton pair production [Fig. 3 (bottom)], in which only same-flavor lepton pairs are produced, we also consider a more focused approach in which events with opposite-flavor dilepton pairs provide a data control sample. We use the $M_{\mathrm {CT}\perp }$ distribution of the opposite-flavor dilepton events to define a template for the flavor-symmetric background. The flavor-symmetric background includes top-quark and WW events, as well as WZ events in which one selected lepton comes from the W boson and the other from the Z boson. By using a single template to account for several different processes, we reduce the number of free parameters, thereby increasing the statistical precision of the search. To accommodate the new template, the diboson template is modified slightly so that it accounts only for non-flavor-symmetric diboson processes: WZ events where both selected leptons come from a Z boson, and ZZ events. The Z$/\gamma ^*$ and non-prompt templates remain unchanged.

**Fig. 11:**
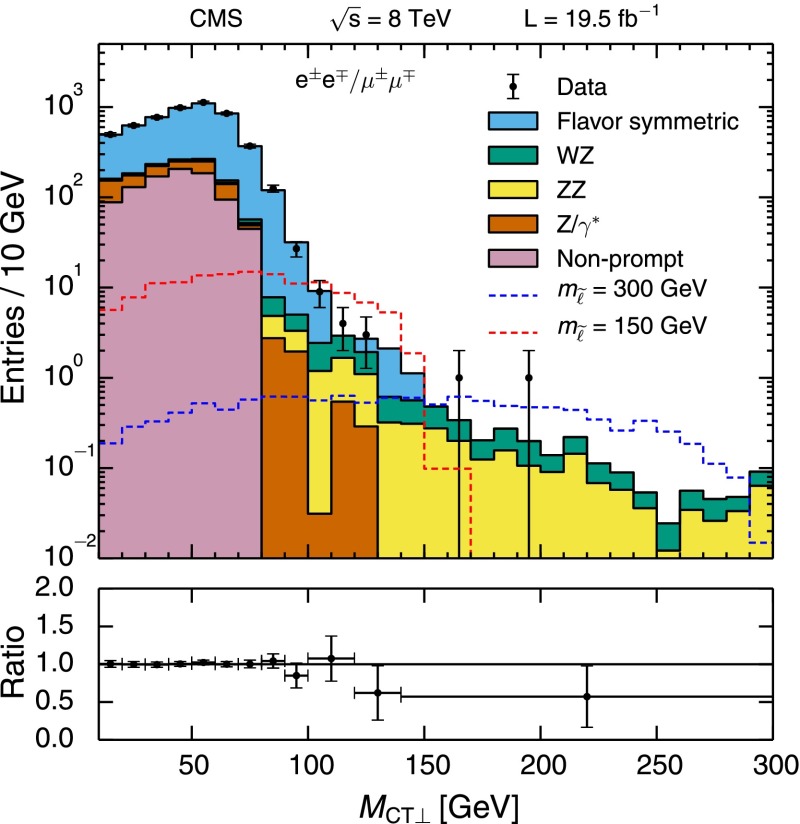
$M_{\mathrm {CT}\perp }$ distribution compared to the background prediction for the same-flavor channel of the non-resonant opposite-sign dilepton analysis, where the background prediction is derived from an alternative template method that uses opposite-flavor dilepton events as a control sample (see text). The signal distributions with two different slepton mass values for the SUSY scenario shown in Fig. 3 (top) are also shown, with the LSP mass set to zero. The ratio of the data to the fitted distribution is shown in the *lower panel*

We perform a maximum likelihood fit of these templates to the measured same-flavor $M_{\mathrm {CT}\perp }$ distribution under the SM-only hypothesis. The results are presented in Fig. 11 and Table 10. The resulting Anderson-Darling $p$ value is 0.22, implying consistency of the data with the SM.

**Table 10:** Results from a maximum likelihood fit of the background-only hypothesis to the $M_{\mathrm {CT}\perp }$ distribution of the same-flavor channel with $M_{\mathrm {CT}\perp }> 10$ GeV, for the non-resonant opposite-sign dilepton analysis, where the background prediction is derived from an alternative template method that uses opposite-flavor dilepton events as a control sample (see text). For comparison, the SM expected yields based on simulation are also indicated

Sample	Same flavor	
	Fit	Simulation
Flavor symmetric	$4,040\pm 490$	3,620
Non-FS diboson	$98\pm 50$	60
$\mathrm{Z}/\mathrm {\gamma }^*$	$330^{+560}_{-330}$	917
Non-prompt	$920\pm 840$	156

## Interpretations of the searches

We now present the interpretation of our results in the context of models for the direct electroweak pair production of charginos, neutralinos, and sleptons. We compute 95 % confidence level (CL) upper limits on the new-physics cross sections using the CL$_\text {s}$ method [64–66], incorporating the uncertainties in the signal efficiency and acceptance described below and the uncertainties of the expected background ($\sigma _{\text {experiment}}$). For each point in the signal parameter space we arrange the search regions according to their expected sensitivity, and compute limits using the results from simultaneous counting experiments in the most sensitive search regions. For the $\mathrm{W}\mathrm{H}$ search we use the search regions that contribute to 90 % of the total signal acceptance. For the other searches, we use the ten most sensitive search regions. The NLO+NLL cross sections from References [34–36] are used to place constraints on the masses of the charginos, neutralinos, and sleptons.

In setting limits, we account for the following sources of systematic uncertainty associated with the signal event acceptance and efficiency. The uncertainty of the integrated luminosity determination is 2.6 % [67]. Samples of $\mathrm{Z}\rightarrow \ell \ell$ events are used to measure the lepton efficiencies, and the corresponding uncertainties (3 % per lepton) are propagated to the signal event acceptance and efficiency. The uncertainty of the trigger efficiency is 5 % for the dilepton and single-lepton triggers used. The uncertainty of the b-jet tagging efficiency results in an uncertainty for the acceptance that depends on the model details but is typically less than 5 %. The energy scale of hadronic jets is known to 1–4 %, depending on $\eta$ and $p_{\mathrm {T}}$, yielding an uncertainty of 1–5 % for the signal event selection efficiency. The larger uncertainties correspond to models for which the difference $\Delta$M between the masses $m_{\widetilde{\chi }}$ and $m_{\widetilde{\chi }^{0} _{1}}$ is small. The experimental acceptance for signal events depends on the level of initial-state radiation activity, especially in the small $\Delta$M region where an initial-state boost may be required for an event to satisfy the selection requirements, including those on $E_{\mathrm {T}}^{\text {miss}}$ and $M_\mathrm {T}$. We use the method of Reference [14] to correct for an observed overestimation in simulation (of up to 20 %) of the fraction of events with a large initial-state boost, and to assign corresponding systematic uncertainties. The signal cross sections are varied by their uncertainties [68] of approximately 5 % to determine the $\pm 1$ standard deviation ($\sigma _{\mathrm {theory}}$) excluded regions.

### Limits on chargino–neutralino production with slepton-mediated decays

We first place limits on the production of chargino–neutralino pairs in models with light sleptons, depicted in Fig. 1, using the results of the three-lepton (Sect. 3) and same-sign dilepton (Sect. 5) searches. Three different scenarios are considered, with different assumptions about the nature of the sleptons, which affect the number of $\tau$ leptons in the final state. These interpretations depend on whether the sleptons are the SUSY partners $\widetilde{\ell } _L$ or $\widetilde{\ell } _R$ of left-handed or right-handed leptons. We consider two limiting cases. In one case, $\widetilde{\ell } _R$ does not participate while $\widetilde{\ell } _L$ and $\widetilde{\nu }$ do: then both diagrams of Fig. 1 exist, and the chargino and neutralino decay to all three lepton flavors with equal probability. Furthermore, two additional diagrams in which the decay $\widetilde{\chi }^{0} _2\rightarrow \ell \,\widetilde{\ell } \rightarrow \ell \,\ell \,\widetilde{\chi }^{0} _1$ is replaced by $\widetilde{\chi }^{0} _2\rightarrow \widetilde{\nu } \,\nu \rightarrow \nu \,\nu \,\widetilde{\chi }^{0} _1$ reduce the fraction of three-lepton final states by 50 %. In the second case, in which $\widetilde{\ell } _R$ participates while $\widetilde{\ell } _L$ and $\widetilde{\nu }$ do not, only the diagram of Fig. 1 (bottom) exists, and there is no reduction in the three-lepton final states. Because the $\widetilde{\ell } _R$ couples to the chargino via its higgsino component, chargino decays to $\widetilde{\ell } _R$ strongly favor production of a $\tau$ lepton. We thus consider three flavor scenarios:

- the “flavor-democratic” scenario: the chargino ($\widetilde{\chi }^{\pm } _1$) and neutralino ($\widetilde{\chi }^{0} _2$) both decay with equal probability into all three lepton flavors, as expected for $\widetilde{\ell } _L$;
- the “$\tau$-enriched” scenario: the chargino decays exclusively to a $\tau$ lepton as expected for $\widetilde{\ell } _R$, while the neutralino decays democratically;
- the “$\tau$-dominated” scenario: the chargino and neutralino both decay only to $\tau$ leptons.

**Fig. 12:**
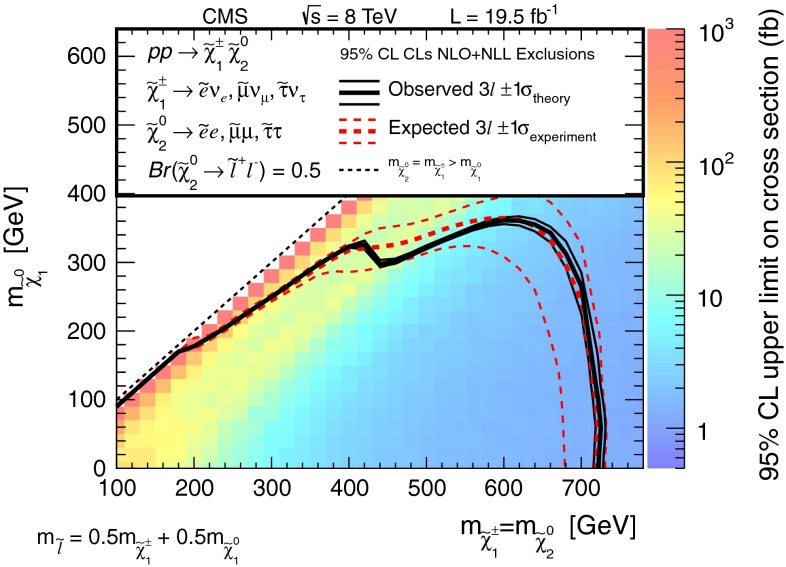
Interpretation of the results of the three-lepton search in the flavor-democratic signal model with slepton mass parameter $x_{\widetilde{\ell }} =0.5$. The *shading* in the $m_{\widetilde{\chi }^{0} _1}$ versus $m_{\widetilde{\chi }^{0} _2}$ ($=m_{\widetilde{\chi }^{\pm } _1}$) *plane* indicates the 95 % CL upper limit on the chargino–neutralino production cross section times branching fraction. The contours bound the mass regions excluded at 95 % CL assuming the NLO+NLL cross sections for a branching fraction of 50 %, as appropriate for the visible decay products in this scenario. The observed, ${\pm }1\sigma _{\text {theory}}$ observed, median expected, and $\pm 1\sigma _{\text {experiment}}$ expected bounds are shown

Figure 12 displays the results from the three-lepton search, interpreted in the flavor-democratic scenario. The figure depicts the 95 % CL upper limit on the cross section times branching fraction in the $m_{\widetilde{\chi }^{0} _1}$ versus $m_{\widetilde{\chi }^{0} _2}$ (${=}m_{\widetilde{\chi }^{\pm } _1}$) plane. The 50 % branching fraction to three leptons is taken into account. The upper limit on the cross section times branching fraction generally becomes more stringent with the increasing mass difference between the chargino or heavy neutralino and the LSP. A drop in sensitivity is observed in the region where this mass difference leads to dilepton pairs with invariant masses close to that of the $\mathrm{Z}$ boson, and is caused by a higher rate for the WZ background.

**Fig. 13:**
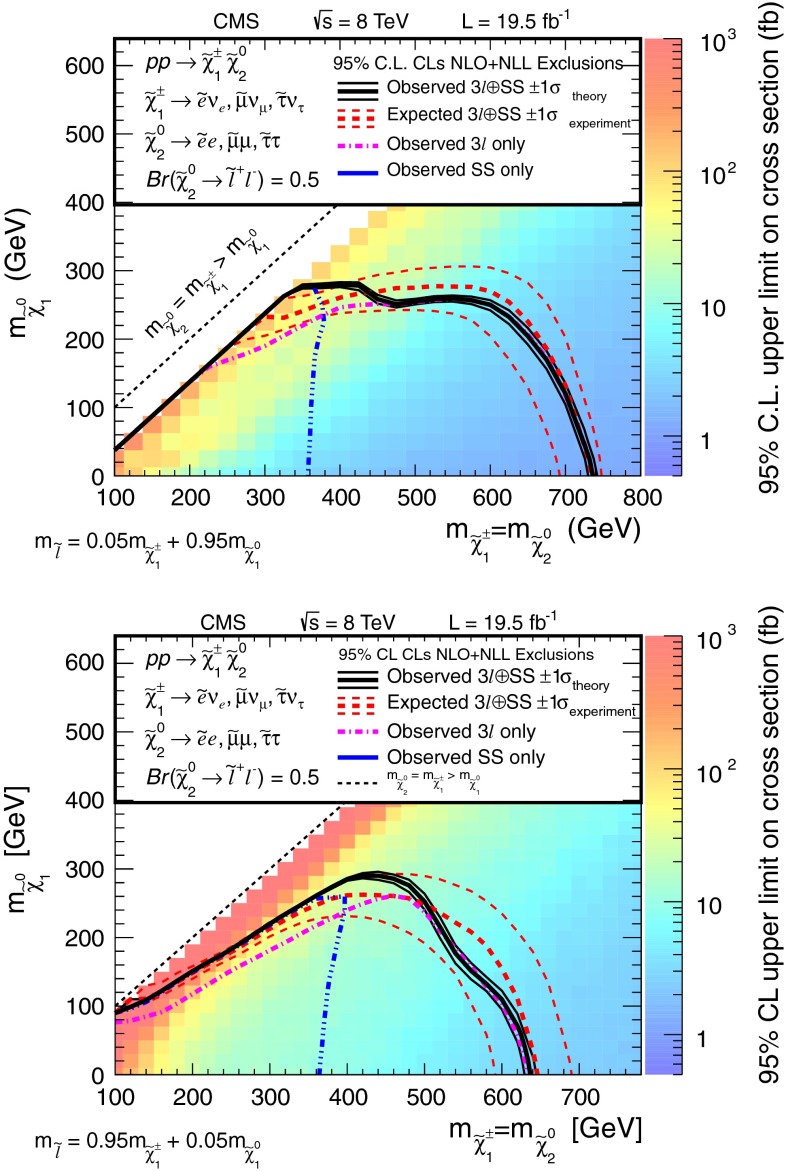
Interpretation of the results of the three-lepton search, the same-sign dilepton search, and their combination, in the flavor-democratic signal model with two different values of the slepton mass parameter: (*top*) $x_{\widetilde{\ell }} =0.05$, (*bottom*) $x_{\widetilde{\ell }} =0.95$. The *shading* indicates the 95 % CL upper limits on the cross section times branching fraction, and the contours the excluded regions assuming the NLO+NLL signal cross sections

The corresponding results for the combination of the SS dilepton and three-lepton searches are shown in Fig. 13 for two values of $x_{\widetilde{\ell }}$ (0.05 and 0.95).

**Fig. 14:**
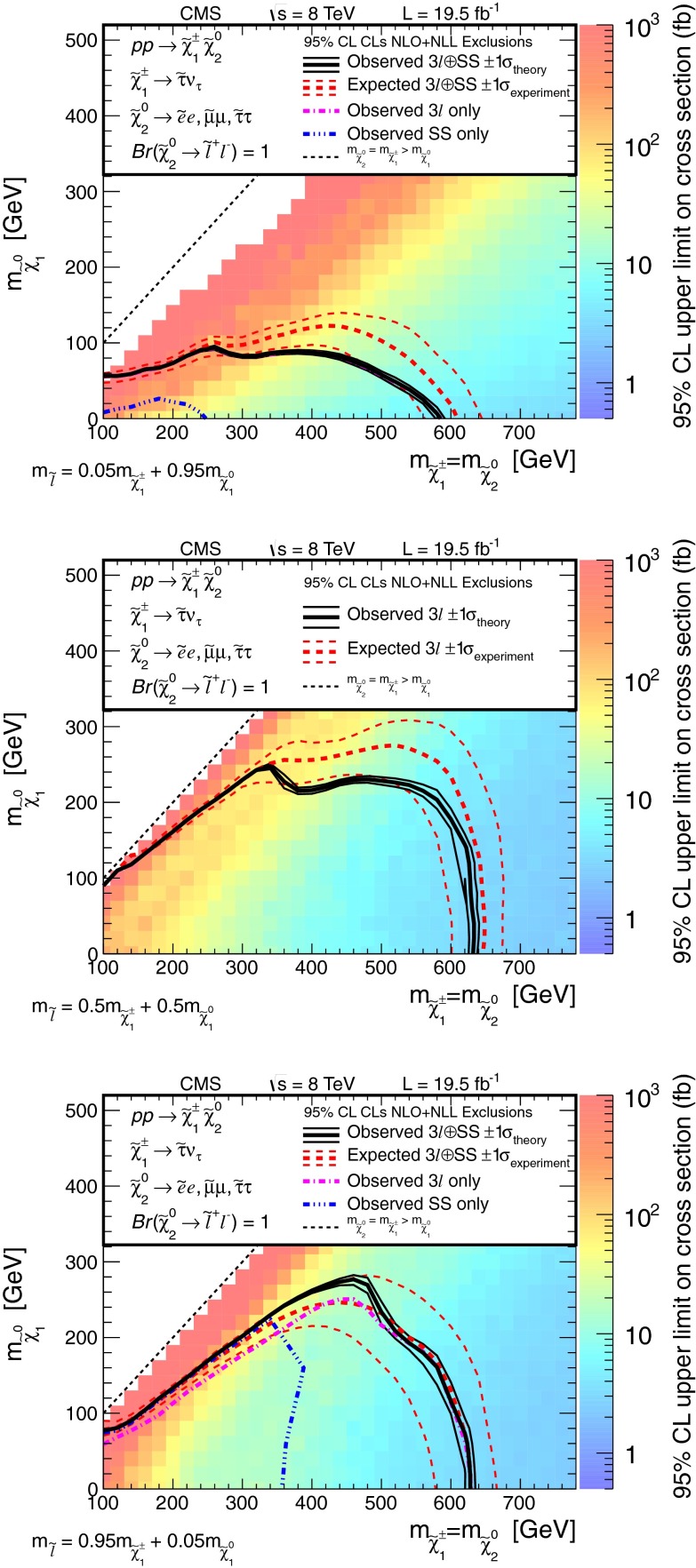
Interpretation of the results of the three-lepton search, the same-sign dilepton search, and their combination, for the $\tau$-enriched signal model with (*top*) $x_{\widetilde{\ell }} =0.05$ and (*bottom*) $x_{\widetilde{\ell }} =0.95$; (*bottom*) interpretation of the three-lepton search for the $\tau$-enriched signal model with $x_{\widetilde{\ell }} =0.5$. The *shading* indicates the 95 % CL upper limits on the cross section times branching fraction, and the contours the excluded regions assuming the NLO+NLL signal cross sections

**Fig. 15:**
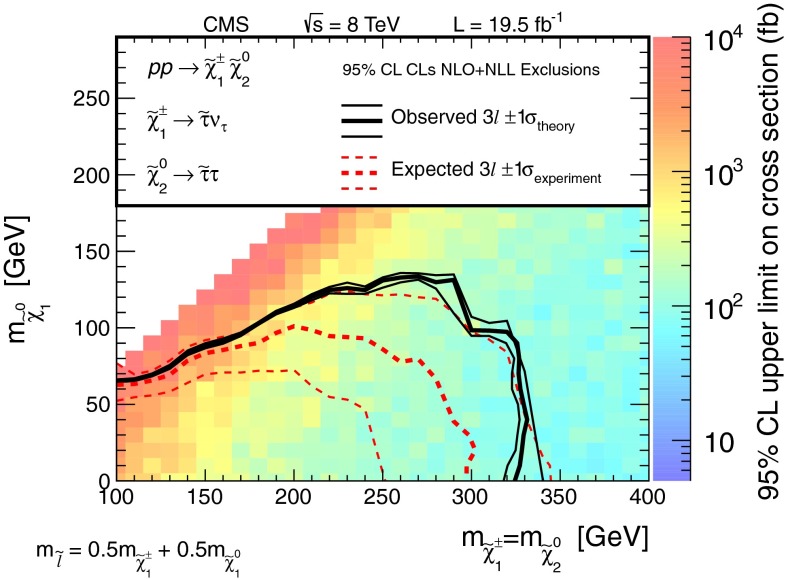
Interpretation of the results of the three-lepton search in the $\tau$-dominated signal model. The *shading* indicates the 95 % CL upper limits on the cross section times branching fraction, and the contours the excluded regions assuming the NLO+NLL signal cross sections

Figure 14 presents the corresponding limits for the $\tau$-enriched scenario and Fig. 15 for the $\tau$-dominated scenario. For the $x_{\widetilde{\ell }} =0.50$ scenario, all three leptons are produced with significant values of $p_{\mathrm {T}}$. As a consequence, the trilepton analysis is more sensitive than the SS dilepton search, for which the limit contours are omitted in Figs. 12, 14 (center), and 15. For the other limit curves in Figs. 13, 14 and 15, the increase in the combined mass limit due to incorporation of the SS dilepton search occurs in the experimentally challenging region where the two neutralinos have similar masses.

For the models with $x_{\widetilde{\ell }} = 0.05$ [Figs. 13 (top), 14(top)], the decay $\widetilde{\tau } \rightarrow \tau \widetilde{\chi }^{0} _{1}$ is not kinematically allowed for signal scenarios with $m_{\widetilde{\chi }^{\pm } _{1}}-m_{\widetilde{\chi }^{0} _{1}} < 20 m_\tau$. Therefore, in this region, the decay $\widetilde{\chi }^{\pm } _{1}\rightarrow \widetilde{\tau } \nu _\tau$ is suppressed. Similarly, in the models with $x_{\widetilde{\ell }} = 0.95$ [Figs. 13 (bottom), 14 (bottom)], the decay $\widetilde{\chi }^{0} _{2}\rightarrow \widetilde{\tau } \tau$ is not kinematically allowed in the region with $m_{\widetilde{\chi }^{0} _{2}}-m_{\widetilde{\chi }^{0} _{1}} < 20 m_\tau$.

### Limits on chargino–neutralino production without light sleptons

We next place limits on chargino–neutralino production under the assumption that the sleptons are too heavy to participate, as depicted in Fig. 2. The chargino is assumed to always decay to a $\mathrm{W}$ boson and the $\widetilde{\chi }^{0} _1$ LSP. The $\widetilde{\chi }^{0} _2$ is expected to decay to a $\widetilde{\chi }^{0} _1$ LSP and either a $\mathrm{Z}$ boson or the Higgs boson. The relative branching fraction ($\mathcal {B}$) for these two decays is in general model-dependent [69]. We thus consider two limiting cases, in which either $\mathcal {B}(\widetilde{\chi }^{0} _2\rightarrow \mathrm{Z}\widetilde{\chi }^{0} _1)=1$ (Sect. 9.2.1), or $\mathcal {B}(\widetilde{\chi }^{0} _2\rightarrow \mathrm{H}\widetilde{\chi }^{0} _1)=1$ (Sect. 9.2.2). The sensitivity in a generic model lies between these two extremes.

#### Limits on chargino–neutralino production in the $\mathrm{W}\mathrm{Z}+E_{\mathrm {T}}^{\text {miss}}$ final state

**Fig. 16:**
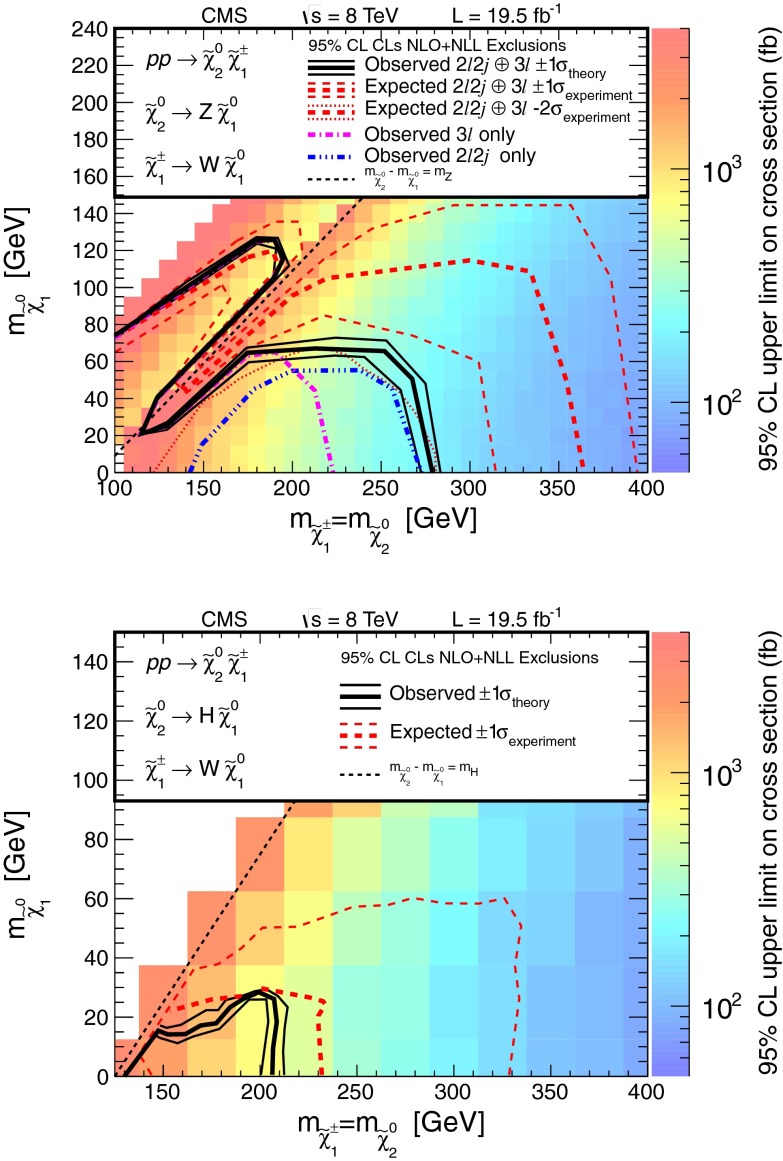
Interpretation of the results of the $\mathrm{Z}+\mathrm {dijet}$ search, the three-lepton search, and their combination, in the $\mathrm{W}\mathrm{Z}+E_{\mathrm {T}}^{\text {miss}}$ model (*top*). Interpretation of the combined results of the single-lepton, same-sign dilepton, and multilepton search regions, in the $\mathrm{W}\mathrm{H}+E_{\mathrm {T}}^{\text {miss}}$ model (*bottom*). The *shading* indicates the 95 % CL upper limits on the cross section times branching fraction, and the contours the excluded regions assuming the NLO+NLL signal cross sections

To evaluate upper limits on the process of Fig. 2 (top), we use the results of the $\mathrm{W}\mathrm{Z}/\mathrm{Z}\mathrm{Z}+E_{\mathrm {T}}^{\text {miss}}$ analysis (Sect. 6) together with the three-lepton analysis (Sect. 3). Figure 16 (top) displays the observed limits for the individual studies and their combination. The sensitivities of the three-lepton and $\mathrm{W}\mathrm{Z}/\mathrm{Z}\mathrm{Z}+E_{\mathrm {T}}^{\text {miss}}$ analyses are complementary, with the three-lepton results dominating the sensitivity in the region where the difference between the neutralino masses is small, and the $\mathrm{W}\mathrm{Z}/\mathrm{Z}\mathrm{Z}+E_{\mathrm {T}}^{\text {miss}}$ results dominating the sensitivity in the region where $m_{\widetilde{\chi }}$ is large. A significant degradation in sensitivity is present in the region of parameter space in which $\Delta M\approx M_Z$, causing the chargino and neutralino decay products to be produced with low momentum in the rest frame of their mother particles. The observed limits are less stringent than the expected limits because the data lie above the expected background in the three-lepton ee and $\mu \mu$ OSSF search regions with $M_\mathrm {T} >160\,\text {GeV}$ and $75<Mll<105\,\text {GeV}$ (see Fig. 5; Table 1).

#### Limits on chargino–neutralino production in the $\mathrm{W}\mathrm{H}+E_{\mathrm {T}}^{\text {miss}}$ final state

To evaluate upper limits for the process of Fig. 2 (center), we combine the results of the single-lepton, SS dilepton, and multilepton searches described in Sect. 7. Figure 16 (bottom) displays the observed limits for the combination of these analyses. The multilepton search provides the best sensitivity at low $m_{\widetilde{\chi }}$, while the single-lepton search dominates at high $m_{\widetilde{\chi }}$. The same-sign dilepton search contributes to the combination at low $m_{\widetilde{\chi }}$. In Appendix C the observed and expected results for the $\mathrm{W}\mathrm{H}+E_{\mathrm {T}}^{\text {miss}}$ final state are presented as a function of $m_{\widetilde{\chi }}$, for a fixed mass $m_{\widetilde{\chi }^{0} _{1}}=1$ GeV, for each of the three search regions and their combination.

### Limits on a $\mathrm{Z}$-boson enriched GMSB model

**Fig. 17:**
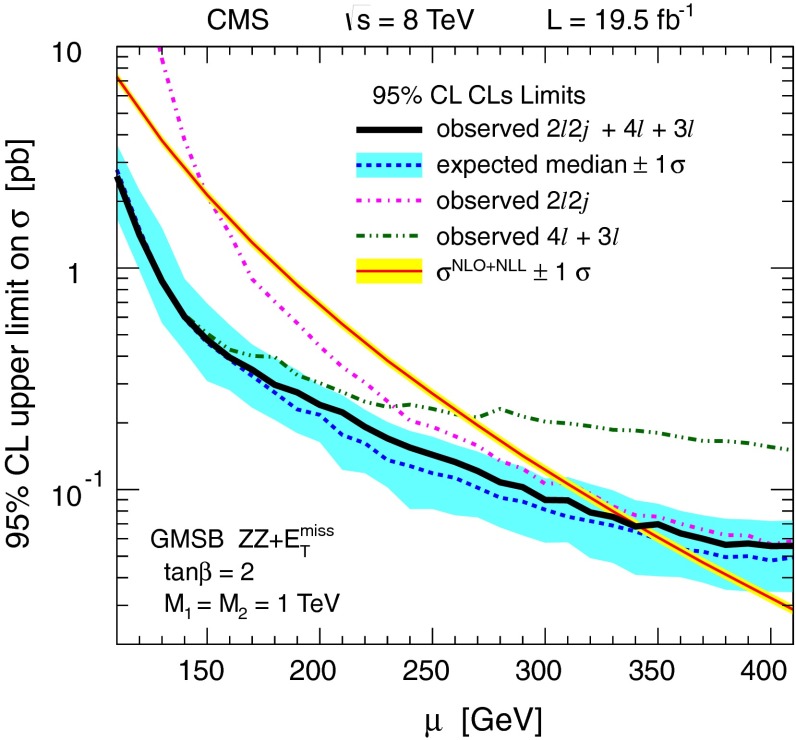
Interpretation of the results of the $\mathrm{Z}+\text {dijet}$ search, the three- and four-lepton searches, and their combination, in the GMSB scenario discussed in the text. The observed and expected 95 % CL upper limits on the cross section are indicated as a function of the higgsino mass parameter $\mu$, and are compared to the theoretical cross section

We also consider a gauge-mediated symmetry breaking (GMSB) $\mathrm{Z}$-boson enriched higgsino model which predicts an enhanced branching fraction to the $\mathrm{Z}\mathrm{Z}+E_{\mathrm {T}}^{\text {miss}}$ final state. The LSP in this model is an almost massless gravitino (${\widetilde{\mathrm{G}}}$), the next-to-lightest SUSY particle is a higgsino $\widetilde{\chi }^{0} _1$, and the $\widetilde{\chi }^{\pm } _1$ and $\widetilde{\chi }^{0} _2$ particles are nearly mass degenerate with the $\widetilde{\chi }^{0} _{1}$. We set the gaugino mass parameters to $M_1=M_2=1$ TeV and the ratio of Higgs bosons vacuum expectation values to $\tan \beta =2$. The results are presented as a function of the higgsino mass parameter $\mu$, where $m_{\widetilde{\chi }^{0} _1} \approx m_{\widetilde{\chi }^{0} _2} \approx m_{\widetilde{\chi }^{\pm } _1} \approx \mu$ to within typical mass differences of a few GeV. The branching fraction to the $\mathrm{Z}\mathrm{Z}+E_{\mathrm {T}}^{\text {miss}}$ final state varies from 100 % at $\mu =130$ GeV to 85 % at $\mu =420$ GeV. We use the results of the three-lepton (Sect. 3), four-lepton (Sect. 4), and $\mathrm{W}\mathrm{Z}/\mathrm{Z}\mathrm{Z}+E_{\mathrm {T}}^{\text {miss}}$ (Sect. 6) searches to constrain the GMSB scenario. The results are presented in Fig. 17.

### Limits on chargino and slepton pair production

**Fig. 18:**
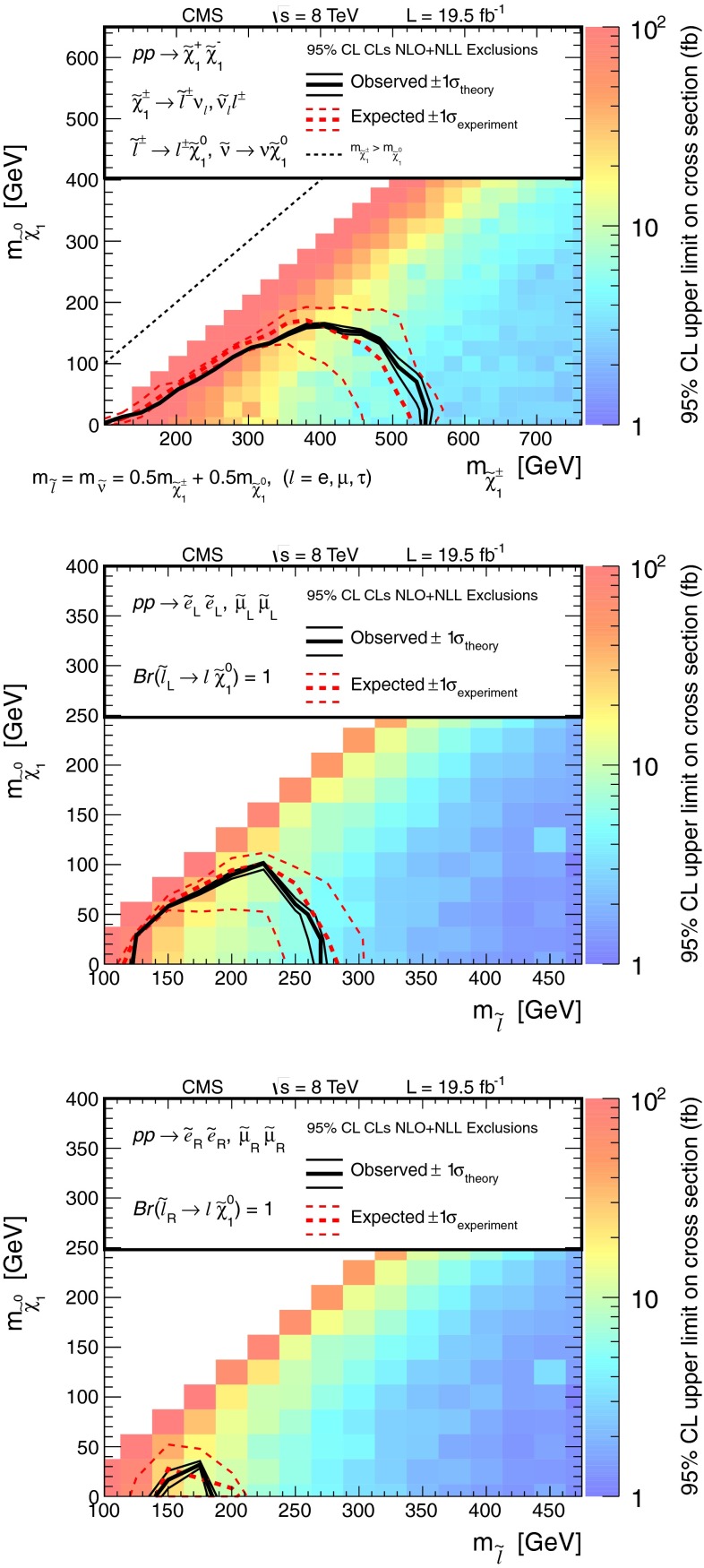
Interpretation of the results of the opposite-sign non-resonant dilepton search, in the models with (*top*) chargino pair production ($\widetilde{\chi }^{\pm } _1\widetilde{\chi } ^\mp _1$), (*center*) left-handed slepton pair production ($\widetilde{\ell } _L \widetilde{\ell } _L$), and (*bottom*) right-handed slepton pair production ($\widetilde{\ell } _R \widetilde{\ell } _R$). The shading indicates the 95 % CL upper limits on the cross section times branching fraction, and the contours the excluded regions assuming the NLO+NLL signal cross sections

Figure 18 shows limits on the chargino and slepton pair-production cross section times branching fraction for the processes of Fig. 3. The limits for chargino pair production are determined using both the opposite- and same-flavor dilepton search regions discussed in Sect. 8, while the limits for slepton pair production are set using only the same-flavor dilepton search region. The production cross sections for left-handed sleptons are larger than those for right-handed sleptons, enhancing the sensitivity.

## Summary

This paper presents searches for the direct electroweak pair production of supersymmetric charginos, neutralinos, and sleptons in a wide variety of signatures with leptons, and $\mathrm{W}$, $\mathrm{Z}$, and Higgs bosons. Results are based on a sample of proton-proton collision data collected at center-of-mass energy $\sqrt{s} = 8$ TeV with the CMS detector in 2012, corresponding to an integrated luminosity of 19.5 $\,\text {fb}^\text {-1}$.

The direct electroweak production of SUSY particles may result in several different signal topologies with one or more leptons and missing transverse energy ($E_{\mathrm {T}}^{\text {miss}}$). The relative sensitivity of each signature depends on unknown parameters, including the SUSY particle masses. This situation, along with the relatively small cross sections typical of electroweak SUSY production, motivates a strategy based on multiple dedicated search regions that target each possible signal topology. In each of these search regions, the data are found to be in agreement with the standard model background expectations. No significant evidence for a signal-like excess is observed.

**Fig. 19:**
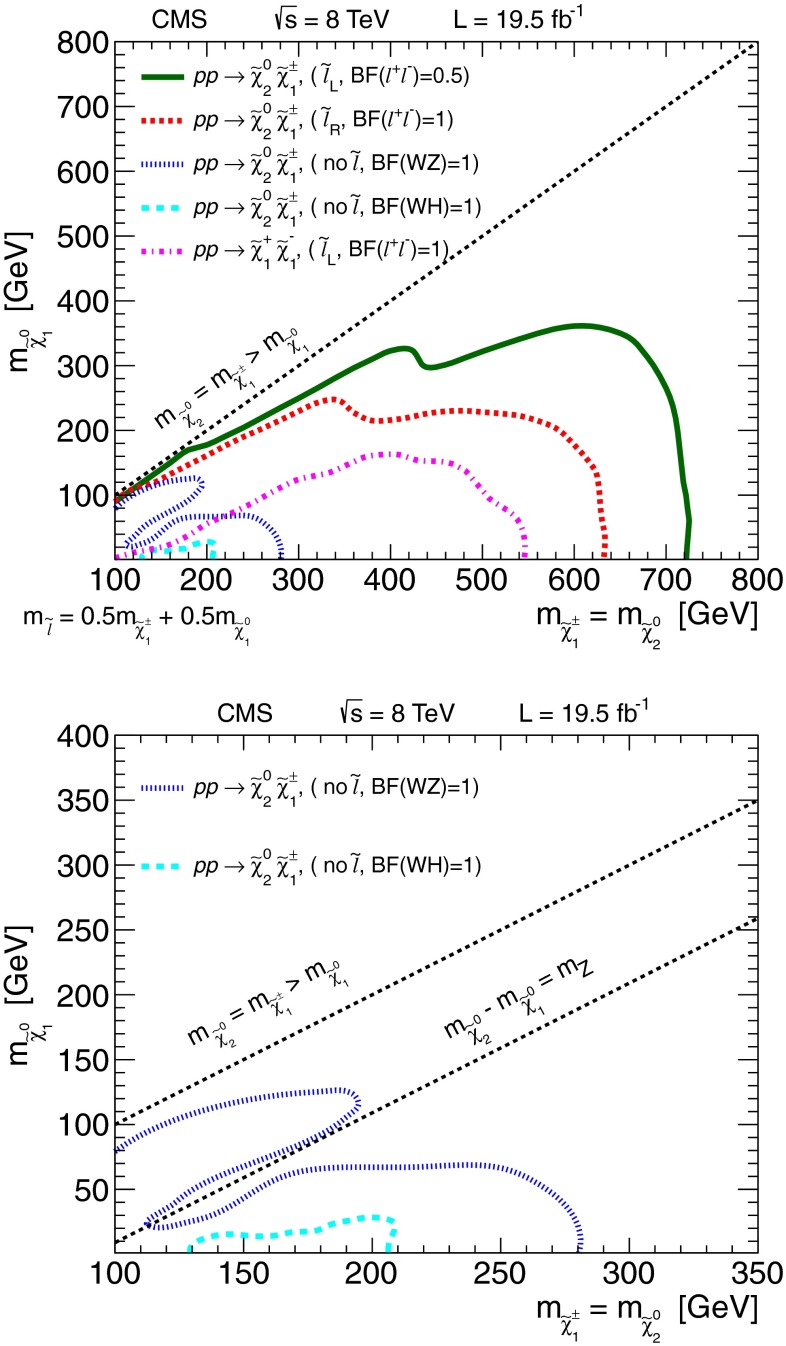
Contours bounding the mass regions excluded at 95 % CL for chargino–neutralino production with decays to left-handed sleptons, right-handed sleptons, or direct decays to Higgs and vector bosons, and for chargino-pair production, based on NLO+NLL signal cross sections (*top*). Where applicable, the $x_{\widetilde{\ell }}$ value used to calculate the slepton mass is 0.5. Expanded view for chargino–neutralino production with decays to Higgs and vector bosons (*bottom*)

**Fig. 26:**
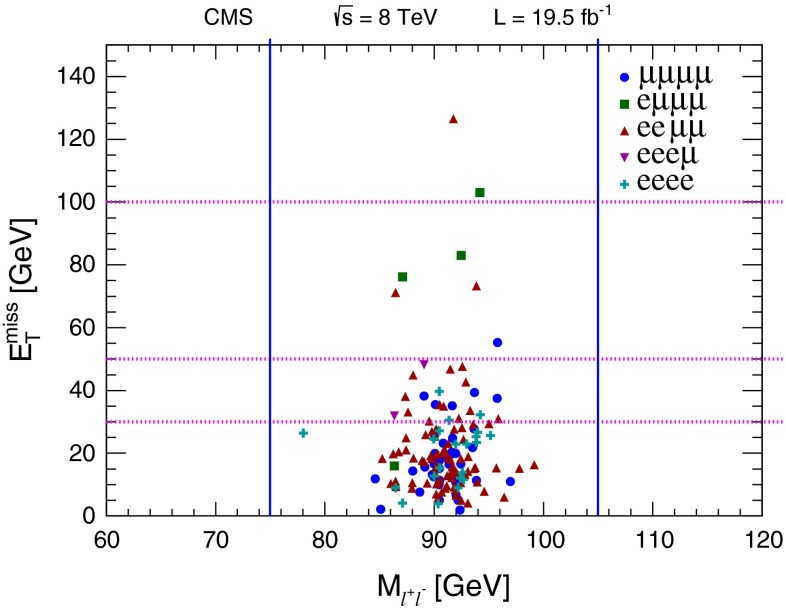
$E_{\mathrm {T}}^{\text {miss}}$ versus $M_{\ell \ell }$ for four-lepton events with an on-Z OSSF pair and no $\tau _\mathrm {h}$. The *legend* indicates the flavor breakdown of events. For events with two OSSF pairs, we choose the pair with mass closest to the $\mathrm{Z}$ boson mass

The results are interpreted in the context of models dominated by direct electroweak SUSY production. Several of the interpretation results are summarized in Fig. 19. We consider models with a wino-like chargino and neutralino pair with degenerate mass $m_{\widetilde{\chi }}$, and a bino-like lightest SUSY particle with mass $m_{\mathrm {LSP}}$. We also consider the presence of light sleptons, either produced in the decays of charginos or neutralinos, or produced directly in pairs. The limits on the signal production cross sections are most stringent in the region of parameter space with large $\Delta M \equiv m_{\widetilde{\chi }}-m_{\mathrm {LSP}}$ (or, for direct slepton production, $\Delta M \equiv m_{\widetilde{\ell }}-m_{\mathrm {LSP}}$), and less stringent in the region of small $\Delta M$, where the final-state objects are less energetic.

The electroweak SUSY process with the largest cross section is chargino–neutralino pair production. The resulting signal topologies depend on the properties of the sleptons. Models with light sleptons enhance the branching fraction to final states with three leptons. Depending on the left/right mixing and flavor of these sleptons, our results probe charginos and neutralinos with masses up to 320, 620, and 720 GeV, for the $\tau$-dominated, $\tau$-enriched, and flavor-democratic scenarios, respectively. In such models, searches in the same-sign dilepton final state enhance the sensitivity in the experimentally challenging region with small $\Delta M$.

Models without light sleptons lead to $\mathrm{W}\mathrm{Z}+E_{\mathrm {T}}^{\text {miss}}$ or $\mathrm{W}\mathrm{H}+E_{\mathrm {T}}^{\text {miss}}$ signatures, with model-dependent branching fractions. To probe the $\mathrm{W}\mathrm{Z}+E_{\mathrm {T}}^{\text {miss}}$ signature, searches in the three-lepton and $\mathrm{Z}$ boson plus jets (with leptonic $\mathrm{Z}$ decay) final states are performed. To probe the $\mathrm{W}\mathrm{H}+E_{\mathrm {T}}^{\text {miss}}$ signature, searches are performed in the single-lepton final state with $\mathrm{H}\rightarrow \mathrm{b}\overline{\text {b}}$, in the same-sign dilepton final state with $\mathrm{H}\rightarrow \mathrm{W}(\ell \nu )\mathrm{W}(\text {jj})$, where j denotes a jet, and in final states with three or more leptons with $\mathrm{H}\rightarrow \mathrm {W^+}\mathrm {W^-}$, $\mathrm{Z}$ $\mathrm{Z}$, or $\mathrm {\tau }^{+}\mathrm {\tau }^{-}$. If the $\mathrm{W}\mathrm{Z}+E_{\mathrm {T}}^{\text {miss}}$ ($\mathrm{W}\mathrm{H}+E_{\mathrm {T}}^{\text {miss}}$) branching fraction is assumed to be 100 %, our results probe charginos and neutralinos with masses up to 270 GeV (200 GeV). The $\mathrm{W}\mathrm{Z}+E_{\mathrm {T}}^{\text {miss}}$ search is particularly important in the region with small $\Delta M$, where we probe charginos and neutralinos with masses up to 200 GeV. We also consider a specific model based on gauge-mediated SUSY breaking that predicts an enhancement in the $\mathrm{Z}\mathrm{Z}+E_{\mathrm {T}}^{\text {miss}}$ production rate. Our results probe higgsinos with masses up to 330 GeV in this scenario.

Following chargino–neutralino pair production, the electroweak SUSY process with the largest cross section is chargino pair production, which leads to a final state consisting of an opposite-sign lepton pair and $E_{\mathrm {T}}^{\text {miss}}$. Our results probe chargino masses up to 540 GeV in a scenario with light sleptons. The direct pair production of sleptons leads to a similar signature, with a lower cross section. For left-handed (right-handed) sleptons, our results probe sleptons with masses up to 260 (180) GeV.

## Figures and Tables

**Fig. 20:**
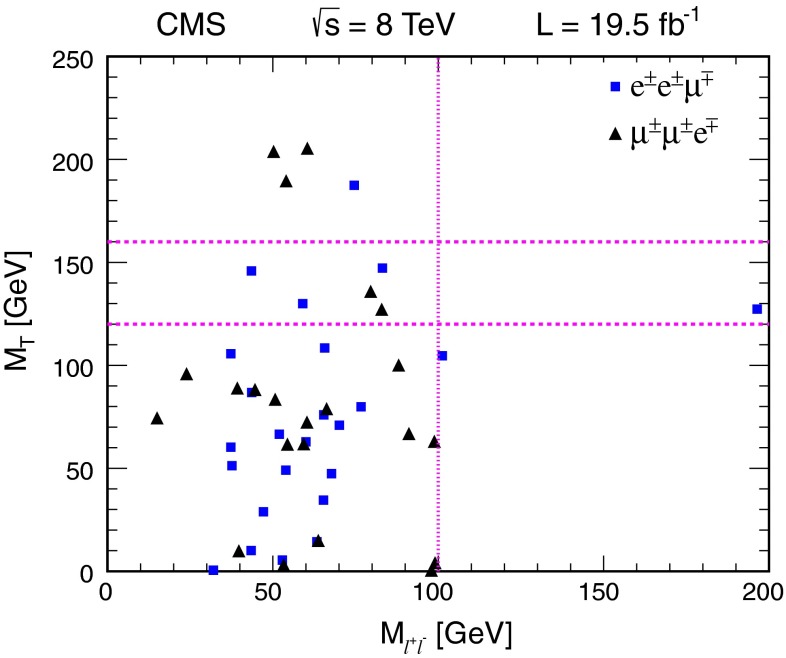
Distribution of $M_\mathrm {T}$ versus $M_{\ell \ell }$ for three-lepton $\mathrm {e}\mathrm {e}\mu$ and $\mathrm {e}\mu \mu$ events without an OSSF pair. $M_{\ell \ell }$ is calculated by combining opposite-sign leptons and choosing the pair closest to the corresponding dilepton mass determined from $\mathrm{Z}\rightarrow \tau \tau$ simulation. $M_\mathrm {T}$ is calculated using the remaining lepton

**Fig. 21:**
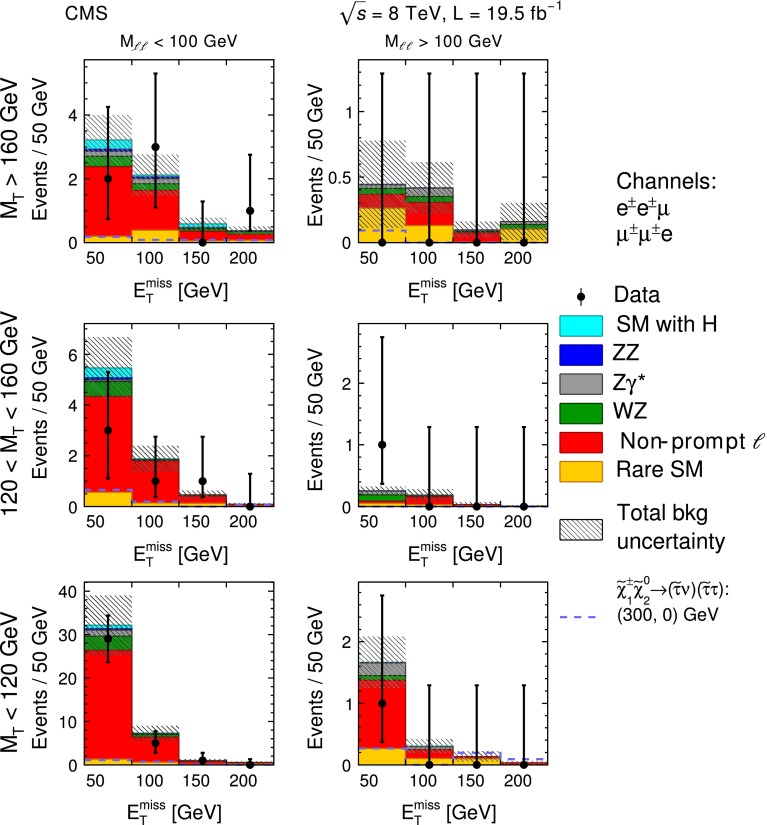
The $E_{\mathrm {T}}^{\text {miss}}$ distributions for three-lepton $\mathrm {e}\mathrm {e}\mu$ and $\mathrm {e}\mu \mu$ events without an OSSF pair. The SM expectations are also shown. The $E_{\mathrm {T}}^{\text {miss}}$ distributions for an example signal scenario is overlaid. The *first* (*second*) *number in parentheses* indicates the value of $m_{\widetilde{\chi }}$ ($m_{\widetilde{\chi }^{0} _{1}}$)

**Fig. 22:**
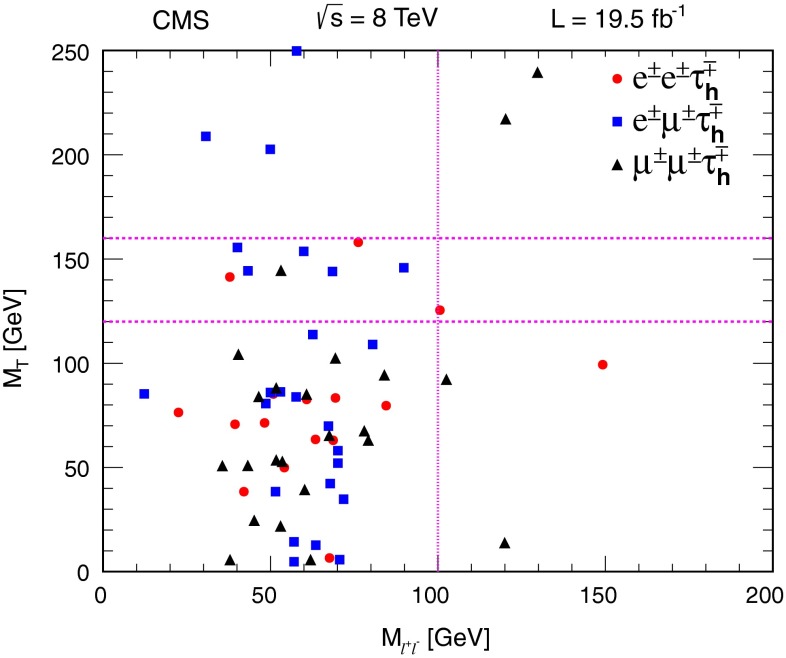
Distribution of $M_\mathrm {T}$ versus $M_{\ell \ell }$ for three-lepton events with a same-sign $\mathrm {e}\mathrm {e}$, $\mathrm {e}\mu$, or $\mu \mu$ pair and one $\tau _\mathrm {h}$. $M_{\ell \ell }$ is calculated by combining opposite-sign leptons and choosing the pair closest to the corresponding dilepton mass determined from $\mathrm{Z}\rightarrow \tau \tau$ simulation. $M_\mathrm {T}$ is calculated using the remaining lepton

**Fig. 23:**
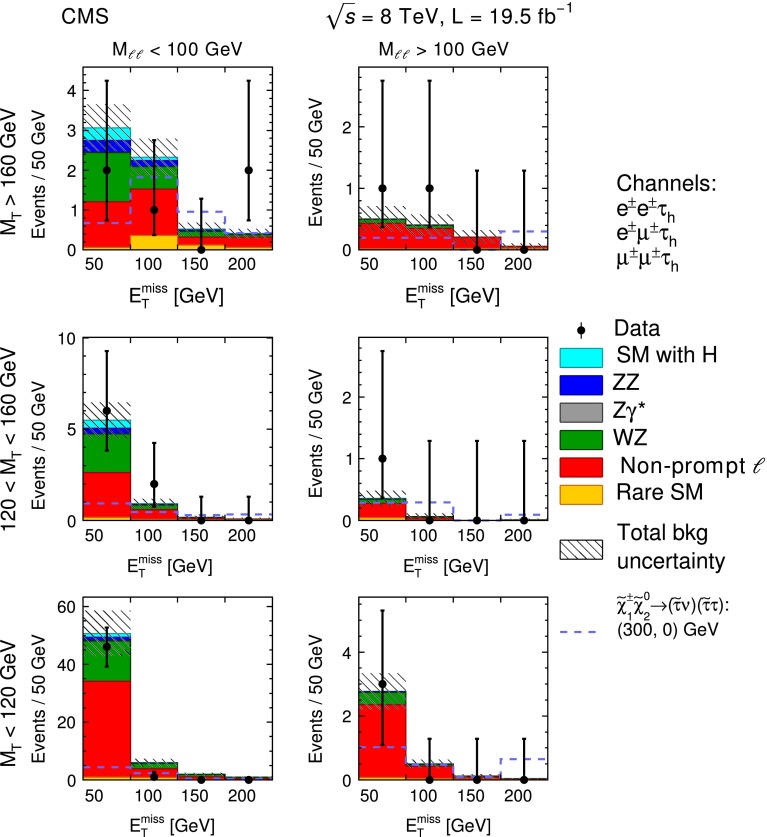
The $E_{\mathrm {T}}^{\text {miss}}$ distributions for three-lepton events with a same-sign $\mathrm {e}\mathrm {e}$, $\mathrm {e}\mu$, or $\mu \mu$ pair and one $\tau _\mathrm {h}$. The SM expectations are also shown. The $E_{\mathrm {T}}^{\text {miss}}$ distributions for an example signal scenario is overlaid. The *first* (*second*) *number in parentheses* indicates the value of $m_{\widetilde{\chi }}$ ($m_{\widetilde{\chi }^{0} _{1}}$)

**Fig. 24:**
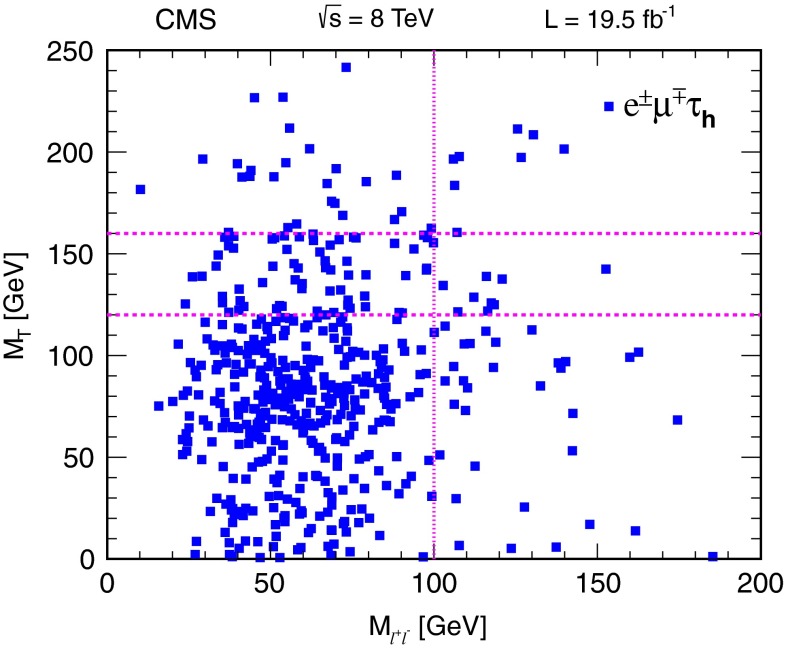
Distribution of $M_\mathrm {T}$ versus $M_{\ell \ell }$ for three-lepton events with an opposite-sign $\mathrm {e}\mu$ pair and one $\tau _\mathrm {h}$. $M_{\ell \ell }$ is calculated by combining opposite-sign leptons and choosing the pair closest to the corresponding dilepton mass determined from $\mathrm{Z}\rightarrow \tau \tau$ simulation. $M_\mathrm {T}$ is calculated using the remaining lepton

**Fig. 25:**
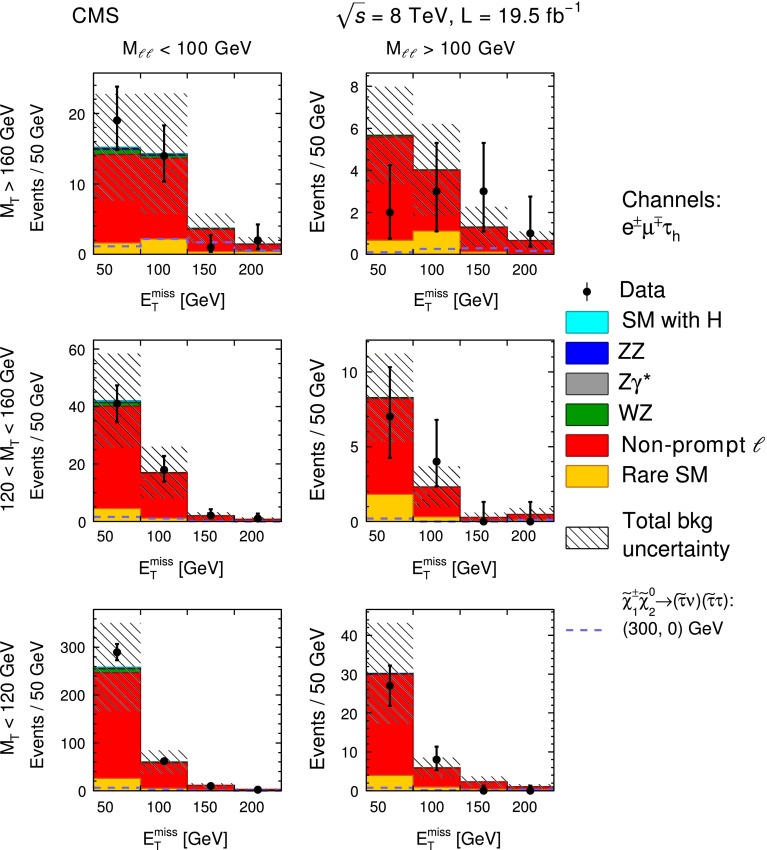
The $E_{\mathrm {T}}^{\text {miss}}$ distributions for three-lepton events with an opposite-sign $\mathrm {e}\mu$ pair and one $\tau _\mathrm {h}$. The SM expectations are also shown. The $E_{\mathrm {T}}^{\text {miss}}$ distributions for an example signal scenario is overlaid. The *first* (*second*) *number in parentheses* indicates the value of $m_{\widetilde{\chi }}$ ($m_{\widetilde{\chi }^{0} _{1}}$)

**Fig. 27:**
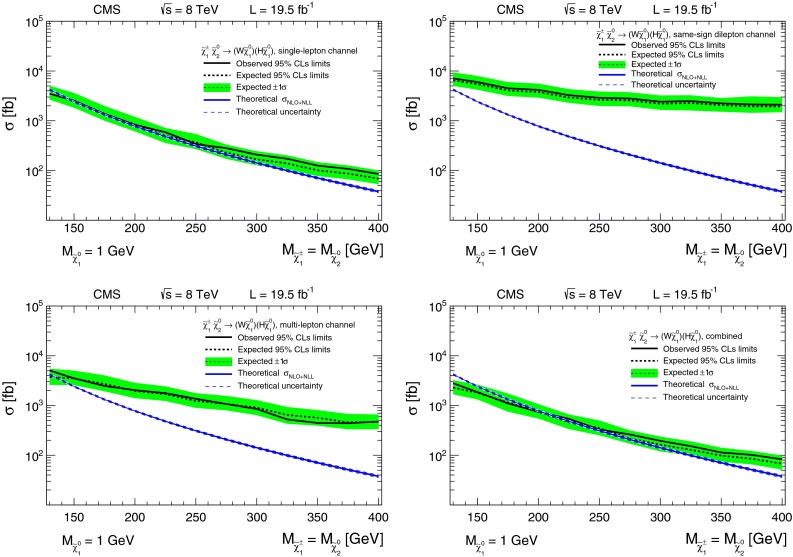
The interpretations of the results from (*upper left*) the single-lepton search, (*upper right*) the same-sign dilepton search, (*lower left*) the multilepton search, and (*lower right*) the combination of the three searches. The*black curves* show the expected (*dashed*) and observed (*solid*) limits on the $\widetilde{\chi }^{\pm } _{1}$ $\widetilde{\chi }^{0} _{2}$ cross section times $\mathcal {B}(\widetilde{\chi }^{\pm } _{1}\widetilde{\chi }^{0} _{2}\rightarrow \mathrm{W}\mathrm{H}+E_{\mathrm {T}}^{\text {miss}})$. The *green band* shows the one-standard-deviation variation of the expected limit due to experimental uncertainties. The *solid blue curve* shows the theoretical prediction for the cross section, with the *dashed blue bands* indicating the uncertainty of the cross section calculation

## Appendix A: Additional plots for the three-lepton and four-lepton searches

This appendix presents additional results from the three-lepton and four-lepton searches. The distributions of $M_\mathrm {T}$ versus $M_{\ell \ell }$ for three-lepton events are presented in Figs. 20, 21, 22, 23, 24 and 25. The corresponding numerical results are presented in Tables  11, 12 and 13. The distribution of $E_{\mathrm {T}}^{\text {miss}}$ versus $M_{\ell \ell }$ for four-lepton events is presented in Fig. 26.

**Table 11:** Observed yields and SM expectations for three-lepton $\mathrm {e}\mathrm {e}\mu$ and $\mathrm {e}\mu \mu$ events without an OSSF pair. The uncertainties include both the statistical and systematic components

$M_\mathrm {T}$ ($\text {GeV}$)	$E_{\mathrm {T}}^{\text {miss}}$ ($\text {GeV}$)	$M_{\ell \ell } < 100$ GeV		$M_{\ell \ell } > 100$ GeV	
		Total bkg	Observed	Total bkg	Observed
$>$160	50–100	3.2 $\pm$ 0.8	2	0.44 $\pm$ 0.33	0
	100–150	2.1 $\pm$ 0.7	3	0.42 $\pm$ 0.19	0
	150–200	0.59 $\pm$ 0.18	0	0.10 $\pm$ 0.06	0
	$>$200	0.37 $\pm$ 0.13	1	0.16 $\pm$ 0.14	0
120–160	50–100	5.5 $\pm$ 1.2	3	0.25 $\pm$ 0.07	1
	100–150	1.9 $\pm$ 0.5	1	0.19 $\pm$ 0.10	0
	150–200	0.46 $\pm$ 0.18	1	0.03 $\pm$ 0.03	0
	$>$200	0.10 $\pm$ 0.05	0	0.008 $\pm$ 0.010	0
0–120	50–100	32 $\pm$ 7	29	1.7 $\pm$ 0.4	1
	100–150	7.3 $\pm$ 1.7	5	0.30 $\pm$ 0.11	0
	150–200	1.0 $\pm$ 0.3	1	0.14 $\pm$ 0.09	0
	$>$200	0.53 $\pm$ 0.24	0	0.03 $\pm$ 0.03	0

**Table 12:** Observed yields and SM expectations for events with a same-sign $\mathrm {e}\mathrm {e}$ , $\mathrm {e}\mu$, or $\mu \mu$ pair and one $\tau _\mathrm {h}$. The uncertainties include both the statistical and systematic components

$M_\mathrm {T}$ ($\text {GeV}$ )	$E_{\mathrm {T}}^{\text {miss}}$ ($\text {GeV}$ )	$M_{\ell \ell } < 100$ GeV		$M_{\ell \ell } > 100$ GeV	
		Total bkg	Observed	Total bkg	Observed
$>$160	50–100	3.1 $\pm$ 0.6	2	0.5 $\pm$ 0.2	1
	100–150	2.3 $\pm$ 0.5	1	0.4 $\pm$ 0.2	1
	150–200	0.5 $\pm$ 0.2	0	0.2 $\pm$ 0.1	0
	$>$200	0.4 $\pm$ 0.1	2	0.06 $\pm$ 0.05	0
120–160	50–100	6 $\pm$ 1	6	0.4 $\pm$ 0.1	1
	100–150	0.9 $\pm$ 0.3	2	0.06 $\pm$ 0.05	0
	150–200	0.3 $\pm$ 0.1	0	0.00 $\pm$ 0.01	0
	$>$200	0.06 $\pm$ 0.08	0	0.01 $\pm$ 0.01	0
0–120	50–100	51 $\pm$ 8	46	2.8 $\pm$ 0.6	3
	100–150	6 $\pm$ 1	1	0.5 $\pm$ 0.1	0
	150–200	2.0 $\pm$ 0.4	0	0.11 $\pm$ 0.07	0
	$>$200	0.9 $\pm$ 0.2	0	0.04 $\pm$ 0.02	0

**Table 13:** Observed yields and SM expectations for events with an opposite-sign $\mathrm {e}\mu$ pair and $\tau _\mathrm {h}$. The uncertainties include both the statistical and systematic components

$M_\mathrm {T}$ ($\text {GeV}$)	$E_{\mathrm {T}}^{\text {miss}}$ ($\text {GeV}$)	$M_{\ell \ell } < 100$ GeV		$M_{\ell \ell } > 100$ GeV	
		Total bkg	Observed	Total bkg	Observed
$>$160	50–100	15 $\pm$ 8	19	5.7 $\pm$ 2.3	2
	100–150	14 $\pm$ 9	14	4.0 $\pm$ 2.2	3
	150–200	3.7 $\pm$ 2.1	1	1.3 $\pm$ 1.0	3
	$>$200	1.5 $\pm$ 1.0	2	0.7 $\pm$ 0.4	1
120–160	50–100	42 $\pm$ 16	41	8.3 $\pm$ 2.9	7
	100–150	17 $\pm$ 9	18	2.3 $\pm$ 1.3	4
	150–200	2.0 $\pm$ 1.2	2	0.27 $\pm$ 0.32	0
	$>$200	0.8 $\pm$ 0.5	1	0.5 $\pm$ 0.4	0
0–120	50–100	259 $\pm$ 93	290	30 $\pm$ 13	27
	100–150	60 $\pm$ 25	62	5.9 $\pm$ 2.6	8
	150–200	11 $\pm$ 5	10	2.3 $\pm$ 1.4	0
	$>$200	2.9 $\pm$ 1.4	2	1.1 $\pm$ 0.6	0

## Appendix B: Additional results for the multilepton analysis

In this appendix, we present similar results as those presented in Table 7 for the multilepton analysis of Sect. 7.3 but for different values of $m_{\widetilde{\chi }}$ (Tables 14, 15, 16, 17).

**Table 14:** Multilepton results for the $m_{\widetilde{\chi }}=150$ GeV, $m_{\widetilde{\chi }^{0} _{1}}=1$ GeV scenario. See Table 7 for details

$N_{\tau _\mathrm {h}}$	OSSF pair	$E_{\mathrm {T}}^{\text {miss}}$ ($\text {GeV}$)	Data	Total SM	Signal
0	Below $\mathrm{Z}$	50–100	142	125 $\pm$ 28	14.9 $\pm$ 2.8
0	Below $\mathrm{Z}$	100–150	16	21.3 $\pm$ 8.0	5.06 $\pm$ 0.86
0	None	0–50	53	52 $\pm$ 12	4.61 $\pm$ 0.99
0	None	50–100	35	38 $\pm$ 15	6.5 $\pm$ 1.1
0	None	100–150	7	9.3 $\pm$ 4.3	2.32 $\pm$ 0.43

**Table 15:** Multilepton results for the $m_{\widetilde{\chi }}=200$ GeV, $m_{\widetilde{\chi }^{0} _{1}}=1$ GeV scenario. See Table 7 for details

$N_{\tau _\mathrm {h}}$	OSSF pair	$E_{\mathrm {T}}^{\text {miss}}$ ($\text {GeV}$)	Data	Total SM	Signal
0	Below $\mathrm{Z}$	50–100	142	125 $\pm$ 28	4.90 $\pm$ 0.91
0	Below $\mathrm{Z}$	100–150	16	21.3 $\pm$ 8.0	2.63 $\pm$ 0.43
0	Below $\mathrm{Z}$	150–200	5	2.9 $\pm$ 1.0	0.61 $\pm$ 0.16
0	None	50–100	35	38 $\pm$ 15	2.31 $\pm$ 0.43
0	None	100–150	7	9.3 $\pm$ 4.3	1.31 $\pm$ 0.26

**Table 16:** Multilepton results for the $m_{\widetilde{\chi }}=300$ GeV, $m_{\widetilde{\chi }^{0} _{1}}=1$ GeV scenario. See Table 7 for details

$N_{\tau _\mathrm {h}}$	OSSF pair	$E_{\mathrm {T}}^{\text {miss}}$ ($\text {GeV}$)	Data	Total SM	Signal
0	Below $\mathrm{Z}$	100–150	16	21.3 $\pm$ 8.0	0.70 $\pm$ 0.13
0	Below $\mathrm{Z}$	150–200	5	2.9 $\pm$ 1.0	0.348 $\pm$ 0.067
0	Below $\mathrm{Z}$	$>$200	0	0.88 $\pm$ 0.31	0.218 $\pm$ 0.041
0	Above $\mathrm{Z}$	150–200	1	2.48 $\pm$ 0.68	0.180 $\pm$ 0.045
1	None	150–200	8	15.1 $\pm$ 7.4	0.44 $\pm$ 0.12

**Table 17:** Multilepton results for the $m_{\widetilde{\chi }}=400$ GeV, $m_{\widetilde{\chi }^{0} _{1}}=1$ GeV scenario. See Table 7 for details

$N_{\tau _\mathrm {h}}$	OSSF pair	$E_{\mathrm {T}}^{\text {miss}}$ ($\text {GeV}$)	Data	Total SM	Signal
0	Below $\mathrm{Z}$	100–150	16	21.3 $\pm$ 8.0	0.167 $\pm$ 0.028
0	Below $\mathrm{Z}$	150–200	5	2.9 $\pm$ 1.0	0.138 $\pm$ 0.025
0	Below $\mathrm{Z}$	$>$200	0	0.88 $\pm$ 0.31	0.137 $\pm$ 0.025
0	None	$>$200	0	0.42 $\pm$ 0.22	0.057 $\pm$ 0.011
1	None	$>$200	3	2.4 $\pm$ 1.1	0.152 $\pm$ 0.038

## Appendix C: One-dimensional exclusion plots in the $\mathrm{W}\mathrm{H}+ E_{\mathrm {T}}^{\text {miss}}$ final state

In Fig. 27, the cross section upper limits for the $\mathrm{W}\mathrm{H}+E_{\mathrm {T}}^{\text {miss}}$ signal model are presented as a function of $m_{\widetilde{\chi }}$, for a fixed mass $m_{\widetilde{\chi }^{0} _{1}}=1$ GeV, both individually from the three search regions and their combination.
